# The Plasticizer Bisphenol A Perturbs the Hepatic Epigenome: A Systems Level Analysis of the miRNome

**DOI:** 10.3390/genes8100269

**Published:** 2017-10-13

**Authors:** Ludivine Renaud, Willian A. da Silveira, E. Starr Hazard, Jonathan Simpson, Silvia Falcinelli, Dongjun Chung, Oliana Carnevali, Gary Hardiman

**Affiliations:** 1 Division of Nephrology, Department of Medicine, Medical University of South Carolina (MUSC),Charleston, SC 29425, USA; renaudl@musc.edu; 2 Laboratory for Marine Systems Biology, Hollings Marine Laboratory, Charleston, SC 29412, USA; 3 Center for Genomic Medicine, Bioinformatics, Medical University of South Carolina (MUSC), Charleston, SC 29425, USA; silveira@musc.edu (W.A.S.); hazards@musc.edu (E.S.H.); jondsimp@gmail.com (J.S.); 4 Library Science and Informatics, Medical University of South Carolina (MUSC), Charleston, SC 29425, USA; 5 Dipartimento di Scienze della Vita e dell’Ambiente, Università Politecnica delle Marche, 60131 Ancona, Italy; falcinelli.silvia@gmail.com (S.F.); o.carnevali@univpm.it (O.C.); 6 Department of Public Health Sciences, Medical University of South Carolina (MUSC), Charleston, SC 29425, USA; chungd@musc.edu; 7 Department of Medicine, University of California, La Jolla, CA 92093, USA

**Keywords:** bisphenol A, epigenome, microRNAs, zebrafish, toxicology, bioinformatics

## Abstract

Ubiquitous exposure to bisphenol A (BPA), an endocrine disruptor (ED), has raised concerns for both human and ecosystem health. Epigenetic factors, including microRNAs (miRNAs), are key regulators of gene expression during cancer. The effect of BPA exposure on the zebrafish epigenome remains poorly characterized. Zebrafish represents an excellent model to study cancer as the organism develops a disease that resembles human cancer. Using zebrafish as a systems toxicology model, we hypothesized that chronic BPA-exposure impacts the miRNome in adult zebrafish and establishes an epigenome more susceptible to cancer development. After a 3 week exposure to 100 nM BPA, RNA from the liver was extracted to perform high throughput mRNA and miRNA sequencing. Differential expression (DE) analyses comparing BPA-exposed to control specimens were performed using established bioinformatics pipelines. In the BPA-exposed liver, 6188 mRNAs and 15 miRNAs were differently expressed (*q* ≤ 0.1). By analyzing human orthologs of the DE zebrafish genes, signatures associated with non-alcoholic fatty liver disease (NAFLD), oxidative phosphorylation, mitochondrial dysfunction and cell cycle were uncovered. Chronic exposure to BPA has a significant impact on the liver miRNome and transcriptome in adult zebrafish with the potential to cause adverse health outcomes including cancer.

## 1. Introduction

Since bisphenol A (BPA) was first synthesized in 1891 by the Russian chemist Alexander Dianin and used in plastic bottles starting in 1957, it has changed our society and has had unforeseen impacts on the health of the ecosystem and mankind due to its hormone-like properties [1]. BPA is an inexpensive and convenient plasticizer commonly used to make polycarbonate plastics and epoxy resins that can be found in daily products such as water and infant plastic bottles, lacquers used to coat food cans and bottle tops, medical devices, thermal paper (i.e., receipts and airplane tickets), flame retardants and water supply pipes to name only a few [1,2]. BPA is one of the highest volume chemicals produced worldwide with approximately 8 billion pounds produced each year [3]. BPA has been detected in surface waters at concentrations as high as 92 nM [4]. The Center for Disease Control and Prevention (CDC) analyzed urine samples from 2500 participants and detected BPA in 92.6% of the participants [5]: 11 nM in adults (over 20 years of age), 13 nM in adolescents (12–19 years of age), and 20 nM in children (6–11 years of age). In maternal and fetal plasma, the highest levels of BPA detected were 82.8 and 40 nM respectively [6]. Data from multiple sources indicate that its ubiquitous presence and continuous exposure to humans may cause adverse health effects including metabolic disorders, sexual dysfunction, obesity and heart diseases [7,8,9,10,11,12,13,14,15]; this has raised concerns and discordance among regulatory agencies worldwide.

BPA acts as an ED because its chemical structure resembles the one of the estrogens Estradiol (E2), a female sex hormone that plays a crucial role during puberty by promoting breast development, female fat distribution and skeletal growth. BPA has been shown to bind and activate the two estrogen receptors ERα and ERβ [16] as well as other nuclear hormone receptors including the androgen receptor (AR) [17], thyroid hormone receptors [18] (TR), G-protein-coupled receptors (GPR) [19], glucocorticoid receptors (GR) [20], pregnane X receptor (PXR) [21], endocannabinoid receptor (CNR1) [22], and estrogen-related receptors γ (ERRγ) [23]. This affinity for multiple receptors confers to BPA a broad range of action and far reaching effects on a variety of physiological pathways in humans and wildlife. BPA has been shown to advance puberty [24], reduce fertility [25,26], change male/female sex ratios in tadpoles and fish [27,28], induce obesity [29], metabolic diseases [13] including hepatosteatosis [30], and cancers [31,32]. 

In the past 20 years, the zebrafish (*Danio rerio*) model has come forward as a valuable tool to study system toxicology and human diseases, including cancer [33,34], even though the murine model remains the most commonly used animal system. Despite numerous discoveries made using murine experimental models, the long gestation time (18–20 days), sexual maturation rate (6–8 weeks), high cost of housing and breeding represent significant limitations, and this model is not particularly well suited for high-throughput screening [34]. These limitations inherent to the murine model have incited the development other model organisms. The zebrafish model offers unique advantages as a system toxicology and cancer model. Its high fecundity, relatively low cost of colony maintenance, and ease of genome manipulation make it an attractive model [35,36,37]. Embryos are transparent through 7 days post fertilization (dpf), and this characteristic can be extended to up to 9–14 dpf with the addition of the melanocyte inhibitor phenylthiourea or with generation of Casper transparent adult zebrafish [38]. Zebrafish transparency, and fluorescent technology to mark signaling proteins or cellular entities, facilitates in vivo visualization of cancer growth, and has already provided key insights into the molecular mechanisms of metastasis [39,40,41]. Additionally, zebrafish offers many mammalian features including an innate immune system functional by 48 hours post fertilization (hpf) [42,43] and an adaptive immune system fully functional at 4–6 weeks post fertilization [44]. Reverse and forward genetic approaches are commonly used to manipulate and characterize zebrafish gene function [45]. The zebrafish genome has been fully mapped [46], and according to Howe et al. [47], 70% of protein-coding human genes are related to genes found in the zebrafish (as compared to 82% in the mouse), and 84% of genes known to be associated with human disease have a zebrafish counterpart. Additionally, zebrafish cancers are histologically and genetically similar to human cancers [48], and the zebrafish model contributed to the rapid translation time (∼2-years) from the initial reports of the role of H_2_O_2_ in neutrophil chemotaxis during wound healing in zebrafish [49] to the first utilizations of such knowledge in human patients [50], highlighting that the zebrafish model is a powerful complement to traditional models to study the cancer epigenome. 

However, like every experimental model used in research, the zebrafish has several limitations and, as Goldsmith et al. [34] stated it, “remains a relatively under-developed model organism with large amounts of untapped potential.” Very few validated zebrafish reagents, such as antibodies and cell lines, are available to the research community. A confounder of using zebrafish to study human disease pathways compared to mice is the teleost-specific whole genome duplication event 350 million years ago [51]. As a consequence, zebrafish have duplicate genes [52], which significantly complicate reverse or forward-genetic approaches. Moreover, even though the zebrafish genome has been fully sequenced, genomic annotation in zebrafish remain limited (Figure S1), and it is often beneficial to project zebrafish genes onto their human orthologs, when available, in order to exploit the richer annotation associated with the human genome. However, this translation can results in the loss of several genes that have no human orthologs [53]. Finally, the administration of drugs and carrier solvents directly to the fish media, bathing the entire fish in these compounds, can result in non-desired toxic side effects. To circumvent this important limitation, the oral gavage approach can be used [54]. Despite these limitations, and with more development of the zebrafish model organism, the relevance and utility of this vertebrate model will continue to grow, and provide a powerful complement to the murine system.

Epigenetics is the study of heritable changes in gene expression caused by mechanisms other than changes in the underlying DNA sequences such as DNA methylation and histone modifications, which might affect various cellular phenomena like cell signaling, proliferation, apoptosis [55,56]. An important aspect of epigenetic regulation is cross-talk with other epigenetic mechanisms [57]. Several cancer cells studies have demonstrated that DNA methylation, histone modifications, and chromatin remodeling are linked to miRNA-mediated mechanisms [58,59]. Interestingly, many microRNAs (miRNAs) control the expression of various epigenetic-modifying enzymes which are involved in carcinogenic processes including DNA methyltransferases (DNMTs), histone deacetylases (HDACs), histone acetylases (HAT) and histone demethylases (HDMs) [60,61,62,63,64,65]. These studies reveal that epigenetics is a complex network of mechanisms that work together in creating an “epigenetic landscape” for the regulation of gene expression at transcriptional and translational levels [57]. miRNAs are emerging as a new class of molecules contributing to cancer formation, and have also been identified as master regulators of key genes implicated in mechanisms of epigenetic-induced chemoresistance [66]. Numerous studies have demonstrated significant epigenetic alterations in drug-resistant cancer cells [67,68] and alteration of miRNAs expression [68,69].

Early exposure to BPA has been shown to induce epigenetic modifications and cause prostate and breast cancer later during adulthood in mice and rats [31,32]. More alarming is the fact that BPA not only affects the specimen directly exposed but also its progeny. Manikkan et al. [29] examined “epigenetic transgenerational inheritance of adult onset disease” in subsequent generations (F3) of outbred Harlan Sprague Dawley rats after gestating females (F0 generation) were exposed to BPA; they concluded that germline epimutations and phenotypic alterations induced by BPA-exposure were transmitted to future generations making the descendants more susceptible to cancer development and progression even though they never were in direct contact with BPA. However, the effects of BPA on the epigenome, including miRNAs, and how this may lead to cancer, have not yet been examined in zebrafish. Santangeli et al. suggested that BPA negatively effects genes related to reproduction in female zebrafish [70] due to changes in histone modifications [71] and DNA methylation status [72]. However this study did not specifically address the role of BPA in the development of cancer in zebrafish and did not examine miRNA signatures.

Our group uses zebrafish as systems toxicology model to gain insights that have the potential to protect and improve human and environmental health. Several studies have exposed zebrafish embryos to BPA in the micro molar range [73,74,75], and more recently to nano molar ranges [76,77,78]. In Martella et al. [22], exposure to 438.6 nM of BPA for 48 h was shown to induce hepatosteatosis in adult female zebrafish. Santangeli et al. [70] exposed females to 22–87.6 nM BPA for 21 days and reported reduced fertility. In this study, guided by these previous studies, we exposed adult male zebrafish to 100 nM BPA for 3 weeks, simulating a long term chronic exposure. The liver was dissected, total RNA were extracted, mRNA and miRNA libraries constructed and subjected to high throughput sequencing (HTS) using standard approaches. We carried out differential expression (DE) analysis and compared BPA exposed and control fish using established bioinformatics pipelines [79,80,81]. We hypothesized that chronic BPA-exposure affects epigenetic factors, including miRNAs, in adult zebrafish and establishes an epigenome that is more susceptible to cancer development.

## 2. Materials and Methods

### 2.1. Zebrafish Care and Bisphenol A Administration

Male zebrafish were housed in aquaria that were individually heated using a 100 W aquarium heater to maintain a temperature of 26–29 °C, and the light–dark cycle was 14:10 h. The pH ranged from 7.0 to 7.6 throughout the duration of the experiment. Aeration and filtration were provided using sponge filters. Fish were fed two times a day with commercial flaked fish food (Tetra, Melle, Germany). Fish were acclimated for one week prior to commencing the experiments. Procedures were performed in accordance with The University of California San Diego, IACUC guidelines. All the animals were treated humanely and with regard for alleviation of suffering Four tanks (80 L/tank) with 20 fish each were prepared for the different experimental groups, two containing 100 nM BPA, and two containing only water (as a negative control). BPA was dissolved in ethanol, and diluted in water to make a stock solution. Working experimental concentrations were prepared starting from this stock working solution. The nominal exposures utilized a continuous flow-through system. After three-weeks exposure fish were sampled and liver samples were then immediately frozen in liquid nitrogen and stored at −70 °C prior to analysis. 

### 2.2. RNA Extraction

Isolation of total RNA from zebrafish liver samples was performed using TRIzol reagent (Invitrogen/Thermo Fisher Scientific, Waltham, MA, USA) and the extracted RNA were further purified using the RNeasy Mini kit (Qiagen, Valencia, CA, USA). All RNA was subjected to on-column digestion of DNA during RNA purification from cells, to ensure highly pure RNA free from DNA contamination. The concentrations were determined by absorbance readings (OD) at 260 nm using an ND-1000 (Nanodrop, Wilmington, DE, USA). RNA was further assessed for integrity with the 6000 Nano LabChip assay from Agilent, (Santa Clara, CA, USA). Only RNA samples with a RIN score of > 7.0 were used for genomic analyses. 

### 2.3. High Throughput Sequencing (HTS)

For the zebrafish RNA-Seq experiments, 10 samples were pooled from control and BPA treated groups respectively. This resulted in 2 pooled control groups and 2 pooled BPA groups. To prepare mRNA-Seq libraries the TruSeq RNA Sample Prep Kit (Illumina, San Diego, CA, USA) was utilized; 100–200 ng of total input RNA was used in accordance with the manufacturer’s protocol. The miRNA-Seq libraries were prepared using with Illumina TruSeq Small RNA Prep kit and 1 μg input RNA. HTS was performed using an Illumina HiSeq2000 with each mRNA library sequenced to a minimum depth of ~50 million reads and each miRNA library sequenced to a minimum depth of ~5 million reads. A single end 50 cycle sequencing strategy was employed. Data were subjected to Illumina quality control (QC) procedures (>80% of the data yielded a Phred score of 30). The miRNA-Seq and RNA-Seq data sets have been submitted to the NCBI Gene Expression Omnibus, with accession number designations GSE102059 and GSE102060 respectively. For the species *Danio rerio*, miRNAs are labeled “dre-miRNAs”, i.e., dre-miR-133a. 

### 2.4. Bioinformatics Analyses

**Figure 1:**
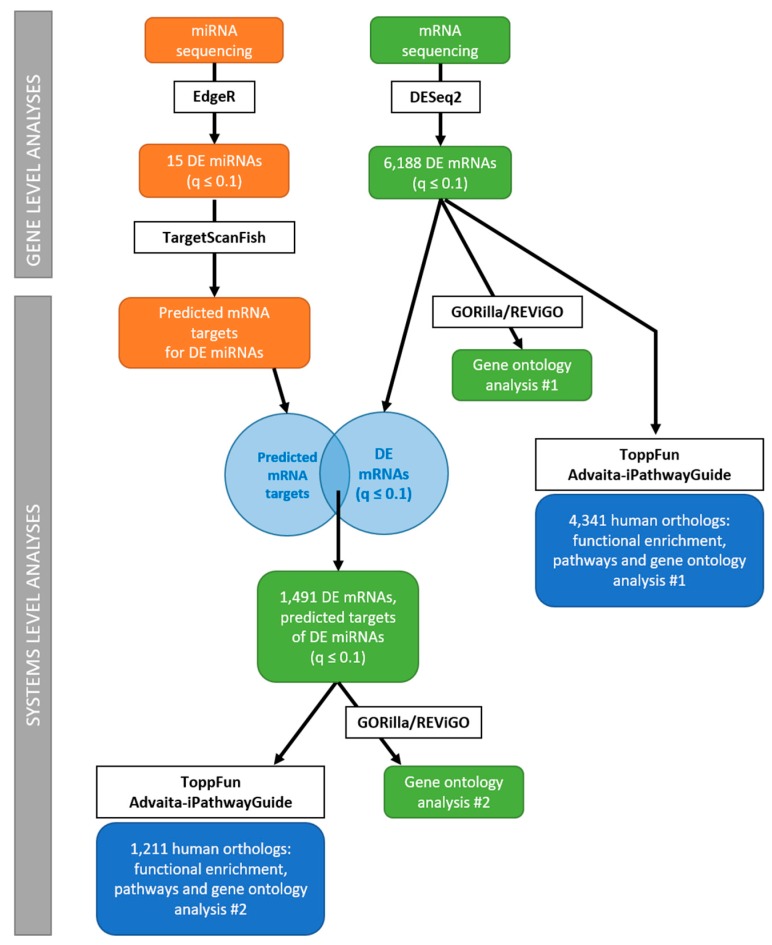
Workflow for analysis of mRNA-Seq and miRNA-Seq data at the gene and systems level.

To take advantage of the fact that we had access to both mRNA-Seq and miRNA-Seq datasets from the BPA exposed liver, we developed a novel workflow (Figure 1) that we describe in greater detail below. 

#### 2.4.1. Gene Level Analyses

Secondary analyses of the mRNA and miRNA-Seq data were carried out on an OnRamp Bioinformatics Genomics Research Platform as previously described by us (OnRamp Bioinformatics, San Diego, CA, USA) [79]. OnRamp’s Advanced Genomics Analysis Engine utilizes an automated RNA-Seq workflow to process data, including (1) FastQC to perform data validation and quality control [79]; (2) CutAdapt [82] to trim and filter adapter sequences, primers, poly-A tails and other unwanted sequences; (3) TopHat2 [83] to align mRNA-Seq reads to GRCz10 zebrafish genome using the ultra-high-throughput short read aligner Bowtie2 [84]; (4) HTSeq [85] to establish counts which represent the number of reads for each transcript; and (5) DESeq2 [86] to perform DE analysis, which enabled the inference of differential signals with robust statistical power. Transcript count data from DESeq2 analysis of the samples were sorted according to their adjusted *p*-value (or *q*-value), which is the smallest false discovery rate (FDR) at which a transcript is called significant. FDR is the expected fraction of false positive tests among significant tests and was calculated using the Benjamini-Hochberg multiple testing adjustment procedure. We set the FDR value (*q* ≤ 0.1).

The Comprehensive Analysis Pipeline for microRNA sequencing data (CAP-miRSeq) was used for read pre-processing, alignment, mature/precursor/novel miRNA detection and quantification, and data visualization [87]. The mRNA-Seq data was aligned to GRCz10 zebrafish genome using miRDeep [87,88], a tool for miRNA identification from RNA sequencing data, and Bowtie. DE analysis was performed with EdgeR [89,90] with the FDR value set at *q* ≤ 0.1. 

Heatmaps of DE transcripts were generated using the heatmap.2 from gplots R-package [91] using the R-log transformation for normalization, Euclidean distance and Ward clustering settings. Venn diagrams were generated using VENNY 2.1 online tool [92].

#### 2.4.2. System Level Analyses

DE zebrafish transcripts were further analyzed with (1) Gene Ontology enRIchment anaLysis and visuaLizAtion (GOrilla) tool to identify and visualize the enriched Gene Ontology (GO) terms [93,94] and (2) REduce & VIsualize Gene Ontology (REViGO) tool to summarize key GO terms by combining redundant terms into a single, representative term based on a simple clustering algorithm relying on semantic similarity measures. [95].

We also exploited Ensembl orthology to append a human gene ID to a given zebrafish gene ID [96]. This “humanized” dataset was analyzed using (1) iPathwayGuide by Advaita Bioinformatics [97], a workflow that analyzes data in the context of pathways obtained from the Kyoto Encyclopedia of Genes and Genomes (KEGG) database [98], GO terms from the Gene Ontology Consortium database [99], miRNAs from both the miRBase [100] and TARGETSCAN databases [101], and diseases from the KEGG database; and (2) ToppFun offered by ToppGene Suite [102], a tool that detects functional enrichment of gene list based on Transcriptome, Proteome, Regulome (TFBS and miRNA), Ontologies, Phenotype (human disease and mouse phenotype), Pharmacome (Drug-Gene associations), literature co-citation, and other features. One of the underlying databases we used in our analyses is the KEGG database, a well-established resource for deciphering the high-level functions and utilities of a biological system from molecular-level information such as RNA-seq data [98]. The most unique data object in KEGG is the molecular networks—i.e., molecular interaction, reaction and relation networks representing systemic functions of the cell and the organism. Experimental knowledge of such systemic functions is captured from literature and organized in the following forms: [i] Pathway map—in KEGG PATHWAY; [ii] Brite hierarchy and table—in KEGG BRITE; [iii] Membership (logical expression)—in KEGG MODULE; and [iv] Membership (simple list)—in KEGG DISEASE.

#### 2.4.3. Network Construction

**Figure 2:**
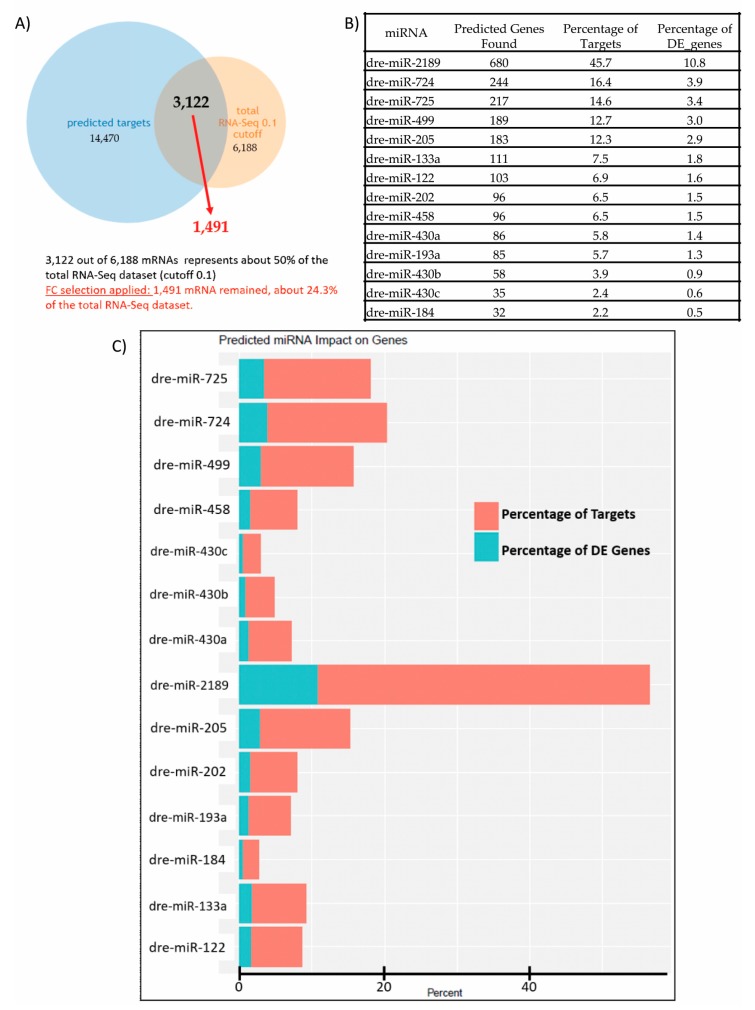
Comparison between predicted mRNA targets and differential expression (DE) mRNAs (*q* ≤ 0.1) obtained from mRNA-Seq analysis. (**A**) Venn diagram comparing the 14,470 predicted mRNA targets obtained from TargetScanFish for all 15 miRNAs identified in Table 1 to the 6188 DE mRNAs in the mRNA-Seq dataset (*q* ≤ 0.1). We found that 3122 mRNAs are common to both lists suggesting that together the 15 miRNAs deregulated by BPA target approximately 50% of the mRNAs DE in the mRNA-Seq data. After applying the FC criterion, 1491 mRNAs remained, suggesting that together the 15 miRNAs deregulated by BPA target approximately 24.3% of the mRNAs significantly DE in the mRNA-Seq dataset (*q* ≤ 0.1); (**B**) For each of the 15 miRNAs identified in Table 1, the sum of the predicted genes found within the mRNA-Seq dataset (*q* ≤ 0.1) was calculated as well the percentage of targets (relative to the 1491 target genes identified) and the percentage of DE genes (relative to the significant DE transcripts in the mRNA-Seq dataset (*q* ≤ 0.1)) that this sum represents; (**C**) Bar plot representing the percentage of mRNA targets and DE genes.

Given that a single miRNA can have far reaching effects by targeting many transcripts for silencing and that one transcript can be targeted by more than one miRNA, our goal was to build a “network” to fully understand the impact that the identified DE miRNAs have on the DE genes identified in our mRNA-Seq dataset and the perturbed pathways and processes they affect. First, for each DE miRNA, predicted targets were identified using TargetScanFish 6.2 [103,104,105]. Next, we generated a “matrix” of DE miRNAs and DE target genes (Table S1) and a table with the sum of the predicted genes found within the DE RNA-Seq dataset (*q* ≤ 0.1) that also includes the percentage of targets (relative to the 1491 target genes identified) and the percentage of DE genes (relative to the DE transcripts in the DE RNA-Seq dataset (*q* ≤ 0.1)) that this sum represents (Figure 2B,C). Based on this matrix, we also generated a dendrogram that represents “miRNA clustering” based on the target mRNAs impacted.

The subsequent step was to create several modules and determine the impact that the identified DE miRNAs have onto these specific modules. Based on gene lists obtained from iPathwayGuide analysis and/or literature review, several modules were defined (Cell cycle, Autophagy and Apoptosis, Oxidative phosphorylation, Epigenetics, Receptors and NAFLD) and heatmaps were generated for each module (Figures 8 and 9). Next, we determined whether or not each gene present in the modules was a predicted target of one or more of the DE miRNAs identified in this study, and a “network” of miRNAs, their predicted targets and the modules they belong to was generated using CytoScape (Table S2 and Figure 10) [106].

## 3. Results

### 3.1. Bisphenol A Exposure Deregulates 6188 mRNAs and 15 dre-miRNAs in the Zebrafish Liver

The RNAseq workflow identified a total of 32,761 zebrafish genes. Following a 3 weeks exposure to 100 nM BPA, 6188 mRNAs were significantly deregulated (*q* ≤ 0.1) in the mRNA-Seq dataset obtained from RNA extracted in liver tissue. Additionally, 15 zebrafish specific miRNAs (*Danio rerio* dre-miRNAs) were significantly deregulated (*q* ≤ 0.1) (Table 1): 14 were upregulated, and 1 was downregulated. We noted that dre-miR-499 was represented twice as -3p and -5p species. Using TargetScanFish, we obtained a list of predicted mRNA targets for each miRNA and merged this list with the DE genes (*q* ≤ 0.1) in the mRNA-Seq dataset (Figure 2A): we found that out of the 14,470 predicted mRNA targets and 6188 deregulated mRNAs (mRNA-Seq dataset, (*q* ≤ 0.1)), 3122 mRNAs were common to both lists, suggesting that collectively these 15 miRNAs deregulated by BPA could target approximately 50% of the mRNAs differently expressed in the mRNA-Seq dataset (*q* ≤ 0.1). Next, for all the upregulated miRNAs, we selected only those with negative fold change (FC) values and for miR-2189 we selected only those with positive FC values. This was based on the rationale that if a particular miRNA is upregulated, its targets for silencing would be downregulated, and vice versa. Once this FC selection criterion was applied, 1491 mRNAs remained, suggesting that together the 15 miRNAs deregulated by BPA target approximately 24.3% of the mRNAs differently expressed in the mRNA-Seq dataset (*q* ≤ 0.1). Subsequently, for each of the DE miRNAs identified in Table 1, the sum of the predicted gene targets found within the mRNA-Seq dataset was calculated as well the percentage of targets (relative to the 1491 target genes identified) and the percentage of DE transcripts (relative to the sum of all transcripts called as significant in the RNA-Seq dataset with *q* ≤ 0.1) (Figure 2B,C): we noted that miR-2189, the only miRNA that was downregulated in the dataset, targets approximately 680 mRNAs present in the mRNA-Seq data (*q* ≤ 0.1), which represents ~45.7% of the 1,491 mRNAs identified after the FC selection criterion was applied and 10.8% of all the significant DE genes in the dataset (*q* ≤ 0.1). 

### 3.2. Gene Ontology Enrichment Analysis

**Figure 3:**
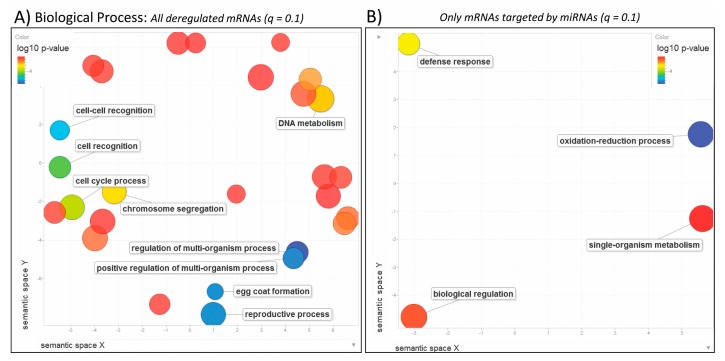
Biological Process. REduce & VIsualize Gene Ontology (REViGO) plots showing categories in 2D space for (**A**) all deregulated mRNAs obtained from DESeq2 (*q* ≤ 0.1) and (**B**) mRNAs that are predicted targets of the miRNAs identified by miRNA-Seq (*q* ≤ 0.1). Blue and green bubbles represent Gene Ontology (GO) terms with more significant *p*-values than the orange and red bubbles. Only the most significant terms are labeled, i.e., regulation of multi-organism and reproductive processes, and oxidation-reduction process. Closeness on the plot reflects semantic similarities between the GO terms. Bubbles of more general terms are larger. Only the most significant terms are labelled when space is an issue.

**Figure 4:**
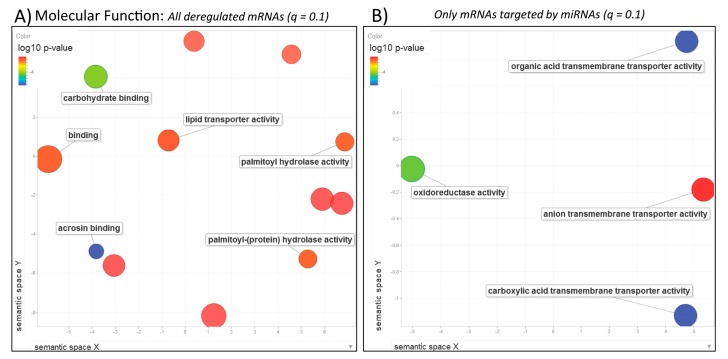
Molecular Function. REViGO scatterplots showing categories in 2D space for (**A**) all deregulated mRNAs obtained from DESeq2 (*q* ≤ 0.1) and (**B**) mRNAs that are predicted targets of the miRNAs identified by miRNA-Seq (*q* ≤ 0.1). Blue and green bubbles are GO terms with more significant *p*-values than the orange and red bubbles. Only the most significant terms are labeled, i.e., acrosin binding and carboxylic acid transmembrane transporter activity. Closeness on the plot reflects semantic similarities between GO terms. Bubbles of more general terms are larger.

**Figure 5:**
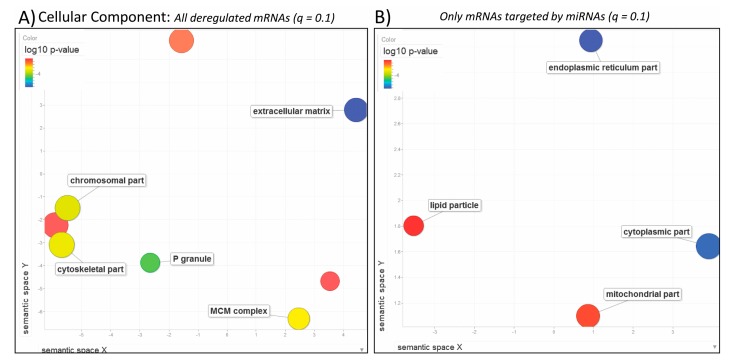
Cellular Component. REViGO plots showing categories in 2D space for (**A**) all deregulated mRNAs obtained from DESeq2 (*q* ≤ 0.1) and (**B**) mRNAs that are predicted targets of the miRNAs identified by miRNA-Seq (*q* ≤ 0.1). Blue and green bubbles are GO terms with more significant *p*-values than the orange and red bubbles, i.e., extracellular matrix and endoplasmic reticulum part. Closeness on the plot reflects semantic similarities between GO terms. Bubbles of more general terms are larger. Only the most significant terms are labelled when space is an issue.

Next we performed GO enrichment analysis of the 6189 transcripts that were deregulated after exposure to BPA. The most significant GO terms were related to reproductive (Figure 3A: regulation of multi-organism/reproductive processes, egg coat formation, cell-cell recognition; Figure 4A: acrosin binding; Figure 5A: extracellular matrix) and cell cycle processes (Figure 3A: cell cycle process, chromosome segregation, DNA metabolism; Figure 4A: mRNA 3’-UTR and microtubule binding; Figure 5A: P-granule, MCM complex). Additionally, we found that several plasma proteins called vitellogenins and zona pellucida glycoproteins, precursor proteins of egg yolk produced in the liver and normally expressed only in the blood or hemolymph of females, were all significantly upregulated in the liver of BPA-exposed male fish (Table S3). This analysis suggests that BPA impacts cell cycle in the liver of exposed specimen and also has an impact on the reproductive system.

When we analyzed only the pool of mRNAs (1491 total) that are potential targets of the miRNAs of interest, other significant GO terms were identified related to oxidative processes (Figure 3B: oxidation-reduction process; Figure 4B: oxidoreductase activity), defense response (Figure 3B), transmembrane transportation (Figure 4B: anion and organic/carboxylic acid transmembrane transporter activity) and cellular parts (Figure 5B: cytoplasmic/endoplasmic reticulum parts). It is interesting to compare GO enrichment analyses of the entire mRNA-Seq dataset to the pool of predicted target mRNAs of the identified miRNAs. This provides a clearer view of the effects of BPA exposure on the miRNome and its role in regulating the transcriptome.

### 3.3. Comparison of Human and Zebrafish Annotations

Based on the number of non-inferred electronic, functional and gene products annotations possible in human and zebrafish, the ratio HUMAN:ZEBRAFISH annotations was determined for each of these categories (Figure S1). In human, non-inferred electronic (grey bar) and functional annotations (orange bar) are 5 times and 2 times better defined respectively than they are in zebrafish. In zebrafish, gene products annotated are slightly better defined than they are in human (blue bar).

### 3.4. ToppFun Functional Enrichment Analysis of the Human Homologs Reveals Several Perturbed Pathways after Bisphenol A Exposure, Including Cell Cycle, Mitochondrial Function, Transcription/Translation and Cancer

We exploited Ensembl homology to append a human gene ID to a given zebrafish gene ID, in order to permit systems analysis using the ‘Transcriptome, ontology, phenotype, proteome, and pharmacome annotations based gene list functional enrichment analysis’ (Toppfun) tool and the richer GO content available for human compared to zebrafish. This revealed cell cycle and transcription-translation-elongation as some of the highest ranked Biological Pathways (Table 2), along with viral infection, mitochondrial energy production and oxidative phosphorylation. Cell cycle, mitosis, chromosome organization, nuclear and cell division represented the highest ranked enriched Biological Process (BP) (Table 3). In terms of Molecular Function (MF), several functions associated with NADH dehydrogenase activity were highly ranked, including quinone and ubiquinone activity, which are enzymes involved in the respiratory chain in the mitochondrial membrane (Table 4). Chromosome and respiratory chain complex were listed in the top 20 highest ranked enriched Cellular Component (CC) terms (Table 5). The ToppFun functional enrichment analysis suggests that exposure to BPA impaires processes such as transcription/translation, cell cycle progression and mitochondrial function, all of which are involved during the development and progression of cancers [107,108,109].

### 3.5. Advaita-iPathwayGuide Analysis of the Human Homologs Reveals Several Perturbed Pathways after Bisphenol A Exposure, Including Cell Cycle, Non-Alcoholic Fatty Liver Disease, Oxidative Phosphorylation and Fanconi Anemia

**Figure 6:**
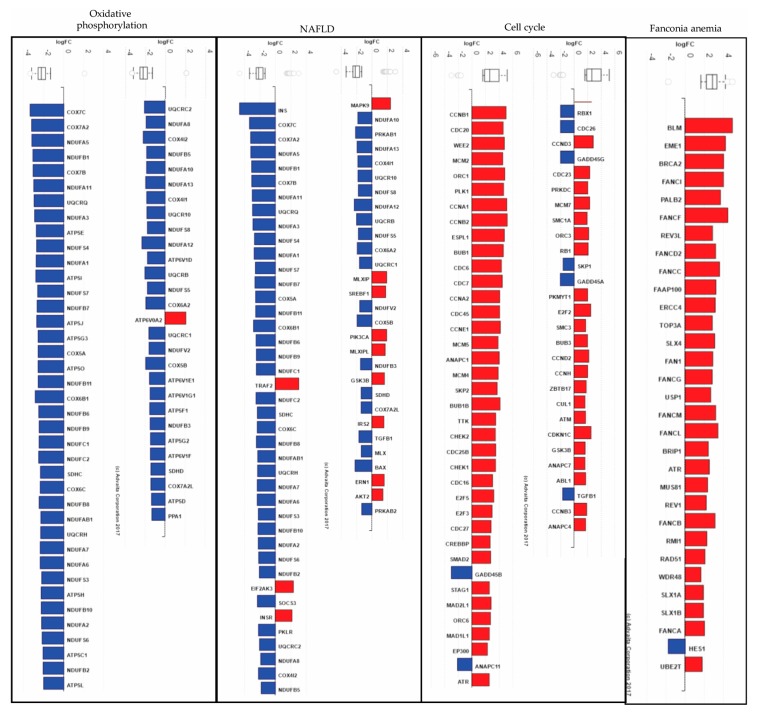
Advaita-iPathwayGuide bar plots. All the genes in Oxidative phosphorylation (KEGG: 00190), NAFLD (KEGG: 04932), Cell cycle (KEGG: 04110) and Fanconia anemia (KEGG: 03460) are ranked based on their log 2 fold change (FC). For each gene, the FC is represented with negative values in blue and positive values in red. The box and whisker plot at the top summarizes the distribution of all gene expression for the specified pathway. The box represents the 1st quartile, the median and the 3rd quartile, while the outliers are represented by circles.

From the 6188 DE zebrafish genes (*q* ≤ 0.1), 4341 have human orthologs that we used as input for system level analysis in Advaita-iPathwayGuide (Figure S2). In summary 13 pathways, 296 GO BP terms, 38 GO MF terms and 123 GO CC terms were found to be significantly enriched (Table S4–7). Oxidative phosphorylation (*q* = 2.66×10^−8^, Figure S3), Non-alcoholic fatty liver disease (NAFLD, *q* = 1.32×10^−4^, Figure S4), Cell cycle (*q* = 1.54×10^−3^, Figure S5) and Fanconi anemia (*q* = 1.83×10^−3^, Figure S6) were in the top 10 highest ranked enriched pathways (Table S4). Most of the genes associated with oxidation phosphorylation and NAFLD pathways were downregulated in the BPA-exposed liver while most of the genes belonging to the Cell cycle and Fanconia anemia pathways were upregulated (Figure 6).

Based on the GO analysis, some of the highest ranked enriched biological processes were related to cell cycle (Cell cycle: *q* = 5.33×10^−20^, Cell cycle process: *q* = 1.28×10^−19^, Mitotic process: *q* = 2.31 × 10^−14^, Nuclear division: *q* = 3.46 × 10^−14^, Mitotic cell cycle: *q* = 4.26 × 10^−14^ and Chromosome organization: *q* = 1.03 × 10^−11^) and to respiratory electron chain (Respiratory electron transport chain: *q* = 2.89 × 10^−7^ and Electron transport chain: *q* = 5.46 × 10^−7^) (Table S5). In terms of molecular function, several enriched MF terms associated with NADH dehydrogenase activity were highly significant (NADH dehydrogenase activity: *q* = 2.45 × 10^−5^, Ubiquinone: *q* = 2.45 × 10^−5^, Quinone: *q* = 2.45 × 10^−5^ and Oxidoreductase activity: *q* = 3.10 × 10^−4^) (Table S6). Terms related to chromosome part (Chromosome: *q* = 4.35 × 10^−14^, Chromosomal part: *q* = 6.46 × 10^−13^, Centromeric region: *q* = 2.93 × 10^−7^) and respiratory chain complex (*q* = 5.65 × 10^−9^) were listed in the top 30 highest ranked enriched cellular components (Table S7). The Advaita-iPathwayGuide analysis suggests that exposure to BPA (1) affects oxidative phosphorylation and cell cycle pathways; (2) perturbs mitochondrial respiratory electron transport chain biological processes and cell components; and (3) is associated with the development of liver disease NAFLD and genetic disease Fanconia anemia.

### 3.6. ToppFun and Advaita-iPathwayGuide Functional Enrichment Analysis of the miRNAs’ Targets

After identifying 1491 mRNAs from the mRNA-Seq dataset that are predicted targets of the 15 deregulated miRNAs obtained from the miRNA-Seq analysis, we performed functional enrichment of the corresponding human homologs with ToppFun. The top 3 most significant pathways were NAFLD (*q* = 1.17 × 10^−2^, Figure S4), Oxidative phosphorylation (*q* = 1.21 × 10^−2^, Figure S3) and Metabolic pathways (*q* = 1.21 × 10^−2^, Figure S7) (Table S8). Pathways related to mitochondrial respiratory electron transport (The citric acid cycle: *q* = 1.58 × 10^−2^, Respiratory electron transport: *q* = 4.24 × 10^−2^) and Insulin signaling pathway (*q* = 4.7 × 10^−2^) were also highly enriched. Out of the top 20 most enriched biological process terms, 17 were related to metabolic processes (Table S9). Only 2 molecular function terms were listed in this analysis: Glutathione peroxidase (*q* = 1.95 × 10^−2^) and Oxidoreductase (*q* = 1.95 × 10^−2^) activities (Table S10). Several cellular component terms associated with mitochondria were highly enriched, including Mitochondrial respiratory chain (*q* = 2.82 × 10^−4^), Oxidoreductase complex (*q* = 1.05 × 10^−3^), and Inner mitochondrial membrane protein complex (*q* = 1.85 × 10^−3^) (Table S11). 

From the 1491 DE zebrafish genes, 1211 have human orthologs that we used as input for system level analysis in Advaita-iPathwayGuide (Figure S8-A). In summary, 2 pathways and 2 cellular component GO terms were found to be significantly enriched (Tables S12 and S13). Adherens junction (*q* = 0.07, Figure S9) and Oxidative phosphorylation (*q* = 0.07, Figure S3) were in the 2 highest ranked enriched pathways (Table S12), followed by Chemical carcinogenesis (*q* = 0.38, Figures S8-B and S10), Ribosome (*q* = 0.44, Figure S11) and NAFLD (*q* = 0.74, Figure S4). Based on the GO analysis, Respiratory chain (*q* = 0.07) and Mitochondrial respiratory chain (*q* = 0.07) were the top 2 most enriched cellular component terms (Table S13), followed by other terms related to Cytosolic large ribosomal subunit (*q* = 0.15) and nuclear lumen (*q* = 0.15). All these pathways and GO terms are also present in the top 20 pathways identified in the previous Advaita analysis of all DE genes (Tables S4 and S7). This analysis using two different approaches of the subset of genes that are predicted targets of the deregulated miRNAs identified in this study suggests that many of the pathways and GO terms that are dysregulated after exposure to BPA are indeed associated with miRNA networks. 

### 3.7. Matrix of miRNAs and Target Genes

**Figure 7:**
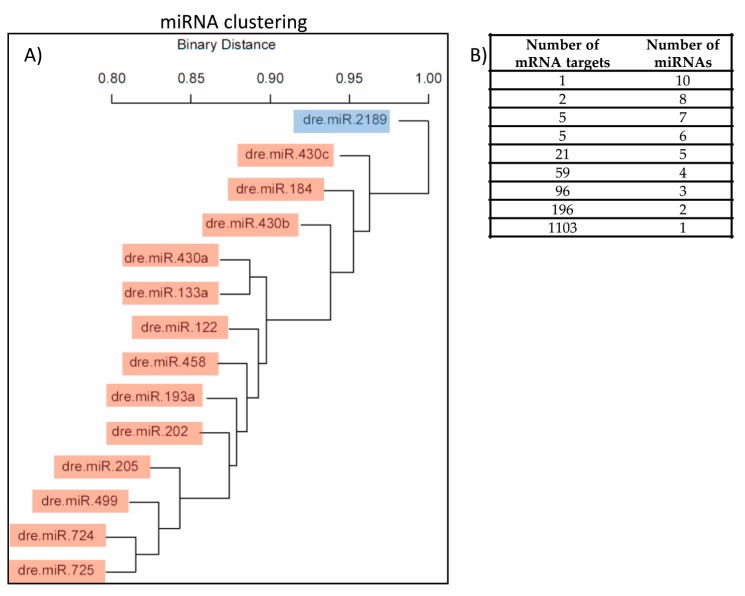
miRNA clustering & matrix. (**A**) miRNAs with similar target mRNAs cluster together. The miRNAs that are upregulated after BPA exposure are highlighted in RED, and dre-miR-2189, the only downregulated miRNA identified in this study, is highlighted in BLUE. For upregulated miRNAs, only predicted gene targets with a negative fold change (FC) present in the mRNA-Seq dataset (*q* ≤ 0.1) were considered. For dre-miR-2189, only predicted gene targets with a positive FC were taken into account. Because dre-miR-2189 is the only miRNA targeting genes with positive FC, its score is equal to 1, showing that it is very different from all other miRNAs shown here. In contrast, dre-miR-725 and dre-miR-724 (left/bottom corner) have low scores and cluster together, meaning that they have several common target genes; in fact 72 mRNAs are predicted targets of these two miRNAs; (**B**) Table recapitulating the number of mRNAs that are predicted to be targeted by 1 or more miRNAs.

Since 15 miRNAs were significantly deregulated after BPA exposure (*q* ≤ 0.1) and many of the predicted target genes of these miRNA of interest were significantly differently expressed in the mRNA-Seq dataset (*q* ≤ 0.1), we aimed to identify whether 1 gene is targeted by more than one miRNA and if certain miRNAs possessed similar signatures and target the same genes. We generated a matrix of miRNAs and target genes (Table S1). This analysis revealed that 385 genes are targeted by more than one miRNA; 1 gene is targeted by 10 different miRNAs, 2 genes by 8 miRNAs, 5 genes by 7 miRNAs, 5 genes by 6 miRNAs, 21 genes by 5 miRNAs, 59 genes by 4 miRNAs, 96 genes by 3 miRNAs and 196 genes by 2 miRNAs (Figure 7B). Based on this matrix we also generated a dendrogram (Figure 7A) that represents miRNA clustering based on the target mRNAs impacted. Because dre-miR-2189 is the only downregulated miRNA and only mRNAs with positive FC were selected as its predicted targets, its score is equal to 1, showing that it behaves very differently from all other miRNAs. For the other 13 upregulated miRNAs, only genes with a negative FC were selected from the list of predicted targets present in the mRNA-Seq dataset. Figure 7 shows that dre-miR-725 and dre-miR-724 (left/bottom corner) have low scores and cluster together, meaning that they have several common target genes; in fact 72 genes are predicted targets of these two miRNAs (Table S1).

### 3.8. Heatmaps for Bisphenol A-Perturbed Functional Modules

**Figure 8:**
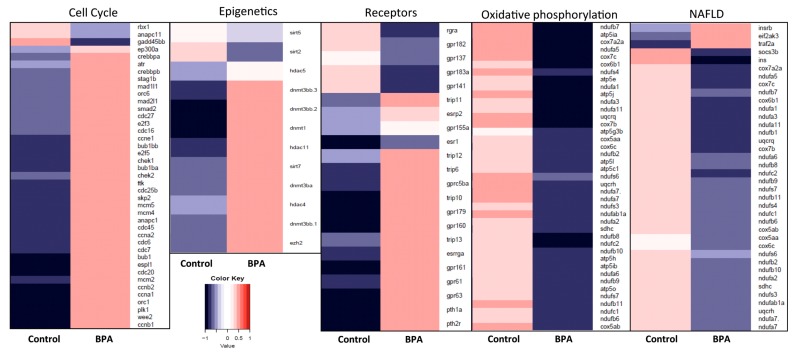
Heatmaps of gene expression changes between BPA exposed and control liver for selected modules of interest. Red and blue boxes indicate relative over- and under-expression with respect to a mean level between the two groups. Only significant DE genes have been included (*q* ≤ 0.1). In the BPA-exposed liver, many transcripts related to Epigenetics and Cell cycle pathways were upregulated compared to control. Many transcripts belonging to the Non-alcoholic fatty liver disease (NAFLD) and Oxidative phosphorylation modules were downregulated in BPA-exposed zebrafish.

**Figure 9:**
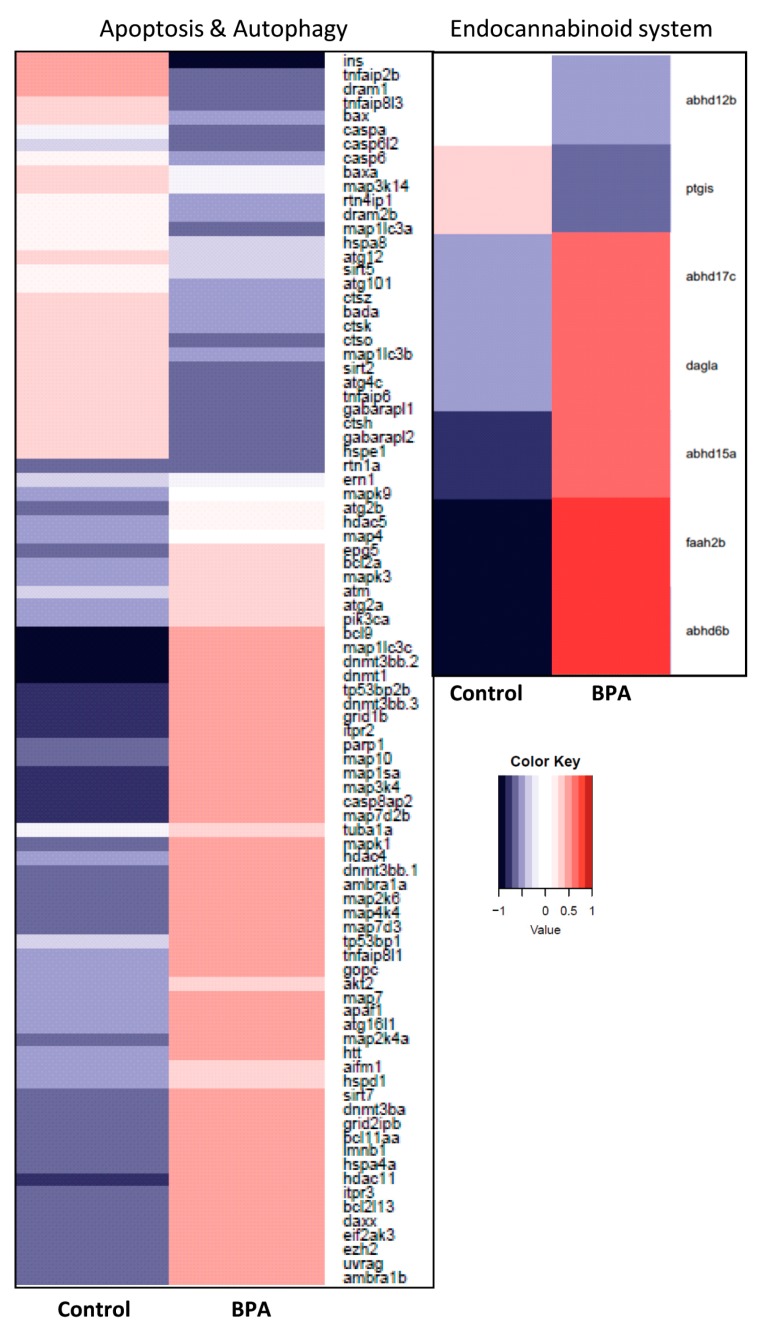
Heatmap of gene expression changes between BPA exposed and control liver for the Apoptosis and Autophagy and Endocannabinoid system modules. Red and blue boxes indicate relative over- and under-expression with respect to a mean level between the two groups. Only significant DE genes have been included (*q* ≤ 0.1).

Based on gene lists obtained from iPathwayGuide analysis and literature review, several modules that include relevant genes were defined: i.e., Cell cycle, Autophagy & Apoptosis, Oxidative phosphorylation, Epigenetics, Receptors, Endocannabinoid system and NAFLD. Heatmaps were generated to visualize the DE genes present in each module based on the mRNA-Seq data (Figure 8 and Figure 9). In BPA-exposed liver, the majority of the genes in the Cell cycle, Apoptosis & Authophagy, Epigenetics, Endocannabinoid system and Receptors modules were upregulated compared to control. All the genes belonging to the NAFLD and Oxidative phosphorylation modules were downregulated in BPA-exposed zebrafish to the exception of three genes (*insrb, eif2ak3* and *traf2a*). 

**Figure 10:**
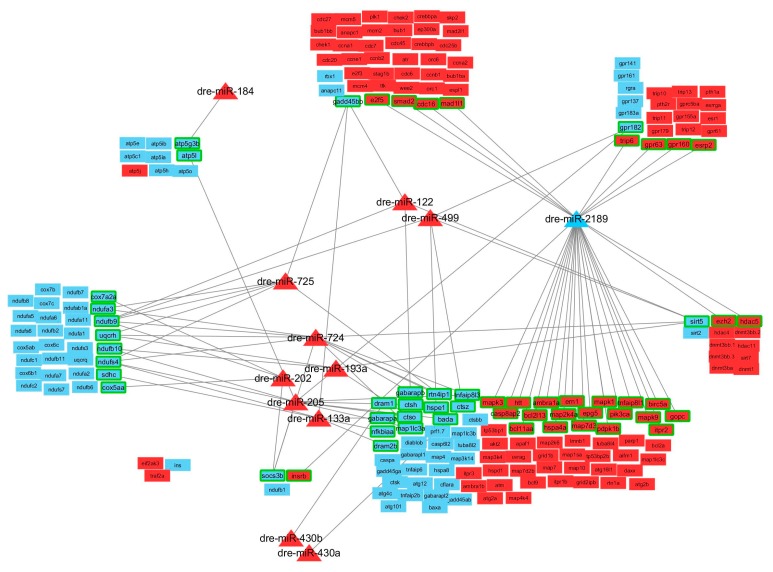
Network of miRNAs, targets and modules. The miRNAs that are upregulated after BPA exposure (positive fold change (FC)) are shown in red triangles, except dre-miR-2189 which was the only downregulated miRNA (negative FC, shown in blue triangle). Each DE gene (*q* ≤ 0.1) present in a module are represented by either a red or blue box based on whether they were up- or down-regulated in the liver after BPA exposure (based on FC values). Genes that are predicted targets of miRNAs are outlined in green. For upregulated miRNAs, only predicted target genes with a negative FC are shown, and for downregulated miRNA (dre-miR-2189), only predicted target genes with a positive FC are shown.

Next, we evaluated the impact of each DE miRNAs on the modules (Figure 10). Note that because certain genes belong to two modules, for clarity we created sub-modules such as “Oxidative phosphorylation and NAFLD”, “Apoptosis and NAFLD” and “Apoptosis/Autophagy and Epigenetics”. This network visualization shows that (1) all modules contain at least 2 genes that are predicted targets of a deregulated miRNA identified in this study, except the module “Apoptosis and NAFLD”; (2) dre-miR-2189, the only DE miRNA that was downregulated, targets many genes in modules that are predominantly upregulated such as Cell cycle (4 target mRNAs), Apoptosis and Autophagy (19 targets), Epigenetics and Apoptosis/Autophagy (2 targets) and Receptors (4 targets); (3) certain miRNAs have only 1 target gene in the selected modules, including dre-miR-184 (“Oxidative phosphorylation”), dre-miR-430a and dre-miR-430b (“Apoptosis and Autophagy”); (4) while other miRNAs have common target genes in the same modules, i.e., dre-miR-725/dre-miR-724/dre-miR-193a, dre-miR-202, dre-miR-205 and dre-miR-133a that have several common target genes in modules “Oxidative phosphorylation and NAFLD”, “Apoptosis/Autophagy”, “NAFLD” and “Cell cycle”.

## 4. Discussion

We investigated in this study the effects of a 3 week exposure to 100 nM BPA on the adult zebrafish liver. This concentration is similar to the high environmental levels detected in river water in Holland [4] and simulates a long term chronic BPA exposure. We determined experimentally that BPA exposure (1) significantly affected the expression of 6188 genes and 15 miRNAs expressed in liver tissue (*q* ≤ 0.1); (2) enriched several GO terms including reproductive and cell cycle processes in these specimen (Gorilla/REViGO analysis). Additionally, based on analysis of the human homologs of the DE zebrafish transcripts; we found (3) other processes such as cell cycle, NAFLD, transcription/translation, metabolic processes and mitochondrial function to be affected by short term exposure to BPA (ToppFun/Advaita analyses). Using a systems level approach to examine and integrate both mRNA and miRNA sequencing datasets; we determined that (4) out of 6188 DE mRNAs, 1491 mRNAs are predicted targets of the 15 deregulated miRNAs, which represent about 24.3% of the transcripts in the RNA-Seq dataset; and that (5) the miRNAs identified regulated many of the enriched biological pathways and GO terms. Furthermore; we showed that (6) BPA exposure altered several specific functional modules such as Epigenetics, Cell cycle, Autophagy/apoptosis, NAFLD and Oxidative phosphorylation. Finally, we showed that (7) several modules were specifically regulated by miRNAs and that “miRNA communities” exercise cooperative regulation. To our knowledge, this is the first study that examined the effect of BPA on the adult zebrafish miRNome using a systems level analysis approach. 

### 4.1. The Effects of BPA on Zebrafish Reproduction

Reproductive processes were amongst the most significantly deregulated biological processes in our GO analysis with GORilla and REViGO, suggesting that BPA has a strong impact on zebrafish reproduction. Growing vertebrate oocytes are surrounded by an extracellular matrix membrane called the zona pellucida, which is required for follicle formation, fertilization, and early development [110,111]; the zona pellucida contains glycoproteins (ZPs) that play an essential role in assembling the extracellular structural coats during oogenesis. At the same time, the oocyte is being filled with yolk proteins (lipovitellin, phosvitin), derived from the plasma proteins vitellogenins (VTGs) found in sexually maturing female fish. VTGs and ZPs are synthesized in the fish liver under endocrine regulation through the hypothalamic-pituitary-gonadal-liver axis [112].Very little VTGs or ZPs, if any, can be detected in male and in juvenile fish, presumably because of low estrogen concentrations, but it is known that these proteins are synthesized by the liver cells of male and juvenile fish treated with 17,β-estradiol [113,114,115]. Here we found that 7 VTGs and 12 ZPs were significantly upregulated in BPA-exposed males, confirming previous findings and highlighting the impact of BPA on the hypothalamic-pituitary-gonadal-liver axis. Maradonna et al. [116] demonstrated that BPA possesses estrogenic activity in seabream by quantifying VTGs and ZP protein levels, and concluded that a different modulation of the different VTG forms was observed, suggesting different regulatory mechanisms for *VTG* genes transcription. *VTG* expression is now commonly used as an environmental biomarker providing evidence on the detrimental action of hormone-mimics substances on reproductive function. 

Santangeli et al. [70] investigated the effects of BPA exposure on epigenetic mechanisms and concluded that the negative effects of BPA on the female reproductive system may be due to its upstream ability to affect histone modifications. Given that deregulation of epigenetics is one of the fundamental prerequisites for tumorigenesis [117] and that endocrine disruptors are carcinogens [118], our goal was to examine how one of the most ubiquitous endocrine disruptors would affect the genome and miRNome of adult zebrafish in the context of cancer biology using a system level approach. Interestingly, several VTGs are targets of miRNAs for silencing [119]: *VTG-3* is targeted by miR-122, the most abundant miRNA in the liver, as well as miR-107, *VTG-7* by miR-107, *VTG-2* by miR-214 and *VTG-6* by miR-23a, highlighting the importance that miRNAs have on vitellogenesis, oocyte maturation and reproduction. 

### 4.2. Bisphenol A Perturbs the Zebrafish Epigenome, Including the miRNome

Epigenetic modifications play critical roles in the control of gene expression in normal and malignant tissues and subsequently affect states of differentiation, activation, and function of all cells [120]. In most instances, histone deacetylation (mediated by histone deacetylases (HDACs) and sirtuins (SIRTs)), histone methylation (mediated by histone methyltransferases such as the enhancer of zeste homolog 2 (EZH2)) and DNA methylation (mediated by DNA methyltransferases (DNMTs)) of regulatory sequences can lead to transcriptional repression [120,121,122]. In the onset of cancer, many tumor-suppressors genes, cell cycle inhibitors, differentiation factors and apoptosis inducers are repressed via epigenetic mechanisms to the advantage of cancerous cells [120]. In utero BPA exposure has been shown to increase risk of prostate and breast cancer later during adulthood by altering DNA methylation in progenitor cells, and increasing EZH2 in mammary glands [31,32]. Manikkam et al. [29] examined “epigenetic transgenerational inheritance of adult onset disease” in subsequent generations (F3) of rats after gestating females (F0 generation) were exposed to BPA; they concluded that germline epimutations and phenotypic alterations induced by BPA-exposure are transmitted to future generations and make them more susceptible to cancer development and progression even though the animals never were in direct contact with BPA.

Here we showed that in adult males exposed to BPA, several DE epigenetic factors in our Epigenetics module (Figure 8) were upregulated, except *sirt5, sirt2* and *hdac5*. This is interesting because this signature suggests that short term exposure to BPA also leads to the acquisition of cancer hallmark capabilities in directly exposed fish via a specific shift in chromatin configuration. As Hanahan and Weinberg [55] mentioned, genome instability is not only established by mutation of tumor-suppressor genes, but also via epigenetic repression and our data suggests that this can occur over a short period of time. Additionally, the bifurcated roles of sirtuins in cancer remain unclear. *SIRT 1* and *SIRT2* both have roles in tumor suppression and promotion [121]; on one hand, functional loss of these genes will promote tumorigenesis because of genomic instability upon their loss, and on the other hand, cancer cells tend to require sirtuins to survive, proliferate, repair the otherwise catastrophic genomic events and evolve. The role of mitochondrial SIRT5 in tumorigenesis has not been evaluated yet but it is a key player in the regulation of metabolic networks and urea cycle [123,124]. Taken together this highlights that BPA is a disruptor of the epigenome and contributes to the establishment of genomic instability and other cancer hallmarks. 

MicroRNAs are an important component of RNA-based mechanisms, one of the three fundamental epigenetic mechanisms of gene regulation; by semi- or full-complementarity, these small non-coding RNAs bind to the 3’ untranslated region (UTR) of target mRNAs along with the RNA-inducing silencing complex (RISC) and either induce inhibition of translation or mRNA degradation [125]. Thanks to this mechanism, miRNAs regulate gene expression without affecting the DNA sequence, a characteristics of epigenetic gene regulation. Each miRNA can target many genes for silencing, and a particular gene can be targeted by more than one miRNA, creating highly complex networks of miRNAs with far reaching regulatory effects on downstream pathways. miRNA networks control the expression of hundreds of protein coding genes and modulate a wide spectrum of biological functions, such as proliferation, differentiation, stress responses, DNA repair, cell adhesion, motility, inflammation, cell survival, senescence and apoptosis, all of which are fundamental to tumorigenesis and cancer cells are capable to hijack almost every step of the miRNA biogenesis pathway to promote dysregulation of miRNA networks [126,127,128].

The effect of BPA exposure on the mammalian miRNome has been investigated in few studies. BPA has been shown to alter the expression profiles of miRNA in human placenta cells [129] and MCF-7 breast cancer cells [130], as well as in sheep ovaries [131] and murine testicular TM4 cell line [132]. To our knowledge, the effect of BPA on miRNA omics has not been investigated in zebrafish yet. Another endocrine disruptor, 2,3,7,8-tetrachlorodibenzo-p-dioxin (TCDD), has been shown to change the expression of several miRNAs in zebrafish embryos (miR-23a, 23b, 24, 27e and 451) that are critical for hematopoiesis and cardiovascular development [133].

Here we show a novel finding that short term exposure to BPA changed the expression of 15 miRNAs in the liver of adult male zebrafish. Zebrafish provide many advantages as toxicology models and miRNAs are currently most extensively studied and identified in the zebrafish compared to other fish models [134]. However, only a few studies have examined the effect of BPA exposure on the zebrafish epigenome [70,135,136,137], and among these, none have specifically considered miRNAs. Approximately 392 dre-miRNAs have been identified and entered in miRBase.org database [100] and several articles have studied some aspect of miRNA function in zebrafish such as their role in basic development and in disease pathways [see Table 4.2 in Freeman et al.’s review [134]]. However this field of research is in its infancy and the function of several dre-miRNAs remain to be defined. Among the 15 dre-miRNAs we identified, only a few have been studied and characterized. 

Of interest because it is expressed specifically in the liver [138,139,140], controls hepatocyte differentiation [141] and gastrointestinal development [140], we found that dre-miR-122 was significantly upregulated after BPA exposure in the adult male liver. In the murine model, overexpression of miR-122 has been shown to perturb hepatic cell differentiation and induce biliary hyperplasia [141], and the authors suggested that monitoring or controlling the expression level of miR-122 might help during programmed in vitro differentiation of stem cells toward hepatocytes for regenerative therapy of liver disease. This is interesting since NAFLD was one of the top 10 highest ranked enriched pathways in our Advaita iPathway analysis and most of the genes in the NAFLD module were downregulated in the BPA-exposed specimens. NAFLD is the build-up of extra fat in liver cells that is not caused by alcohol (if more than 5–10% of the liver’s weight is fat, then it is called a fatty liver “steatosis”) and it may cause the liver to swell (steatohepatitis), scarring (cirrhosis) and may even lead to liver cancer or liver failure over time [142]. BPA steatotic effects have been demonstrated in both zebrafish liver as well as in HHL5 cells in a CB1-dependent manner showing the ability of BPA to produce hepatosteatosis in zebrafish and human hepatocytes by the up-regulation of the endocannabinoid system [22].

Gankyrin is a small ankyrin-repeat protein that is consistently overexpressed in human gastrointestinal (GI) cancers [143,144]. In gankyrin transgenic zebrafish, dre-miR-122 upregulation was associated with dysregulated metabolism and apoptosis in the liver [145]. Additionally, inhibition of miR-122 in mice led to a reduced fatty-acid synthesis rate, substantial reduction of liver steatosis and accumulation of triglycerides [146], implicating miR-122 as a key regulator of cholesterol and fatty-acid metabolism in the adult liver. MiR-122 has also been characterized as a tumor suppressor miRNA affecting hepatocellular carcinoma intrahepatic metastasis by angiogenesis suppression, and its mode of action has been associated with the regulation of the disintegrin and metalloprotease 17 (ADAM17) [147]. 

Another interesting finding in our study is that dre-miR-430a, -430b and -430c were in the top 5 most DE miRNAs, all significantly upregulated after BPA exposure in adult male liver. The zebrafish miR-430 family, which is first expressed during maternal to zygotic transition (MZT), is the most abundant miRNA family during early embryogenesis [148], and has been shown to be essential during zebrafish development [149,150] with striking impacts on brain morphogenesis [151]. A detailed analysis of predicted miR-430 targets revealed that more than 40% of these targets were maternal transcripts that were degraded at MZT [149], highlighting that miR-430 acts as a “developmental switch” by clearing maternal transcripts to facilitate the transition to zygotic programs. In adult male zebrafish, dre-miR-430a, -430b and -430c continue to be expressed, but not in adult female zebrafish [148]. Here we show BPA induced upregulation of these miRNAs in the adult male liver. Further characterization is needed to fully understand the impact elevated levels of these miRNAs might have on the zebrafish liver. 

### 4.3. The Effects of BPA on miRNAs, Their Targets and Downstream Pathways

We determined that half of the DE genes in our mRNA-Seq dataset were predicted targets of the 15 deregulated miRNAs we identified according to TargetScanFish database. Once we applied a fold change (FC) selection (for upregulated miRNAs, only predicted targets with a negative FC were considered, and for miR-2189, the only downregulated miRNA we identified, targets with positive FC were selected), we determined that 24.3% of the DE genes in the mRNA-Seq data were predicted to be regulated by the miRNAs of interest in this study. We subsequently examined the effects of BPA on GO term functional enrichment specifically for that pool of genes and compared it to the information we gathered for the entire DE dataset. REViGO revealed that many significant terms were related to mitochondrial function and metabolism, including Oxidation/Reduction process, Single-organism metabolism, Transmembrane transporter activity of carboxylic acid, Oxidoreductase activity and Mitochondrial part (Figure 3B, Figure 4B and Figure 5B). Both ToppFun and Advaita analyses of the miRNA’s targets also identified enriched pathways and GO terms associated with mitochondrial metabolism such as Oxidative Phosphorylation, Metabolic pathways, Mitochondrial respiratory chain, Oxidoreductase complex, Inner mitochondrial membrane protein complex and Glutathione peroxidase, which has been shown to regulate mitochondrial function by modulating redox-dependent cellular responses [152]. 

Two different analytical approaches (ToppFun and Advaita) using the same pool of transcripts also revealed that NAFLD and Insulin signaling were perturbed by BPA exposure. Under normal conditions, insulin signaling triggers glucose uptake into body cells to be used for energy and inhibits the body from using fat. Consequently, the concentration of glucose in the blood stays within the normal range even when a large amount of carbohydrates is consumed. Insulin resistance is a pathological condition in which cells fail to use insulin effectively, leading to high blood insulin and sugar levels [153]. Interestingly, patients with NAFLD almost universally have hepatic insulin resistance [154] and NAFLD has been shown to be a risk factor for the development of hepatocellular carcinoma [155]. Epigenetic regulation of key enzymes in hepatic fatty acid β-oxidation has been shown to be associated with early-life BPA exposure and the development of the NAFLD phenotype in adult males [156]. Recently, the circulating miRNA signature associated with NAFLD progression has been examined, and miR-122 was the only deregulated miRNA allowing distinction between simple steatosis (SS) and non-alcoholic steatohepatitis (NASH) [157]. 

Chemical carcinogenesis was another significantly enriched pathway identified by Advaita iPathway Guide (Figures S8-B and S10) and all genes in this module were downregulated after BPA exposure, including several cytochrome P450 enzymes (CYPs) such as *CYP1A1* and *CYP3A4*, N-acetyltransferase (*NAT*) and cytosolic glutathione transferase (*GST*). CYPs, NAT and GST are markers of genetic susceptibility in human environmental toxicology [158]. The CYPs catalyze the monooxygenase reaction using molecular oxygen and equivalent electrons transferred from the NADPH-P450 reductase in the endoplasmic reticulum, or from ferredoxin and ferredoxin-reductase in mitochondria [159]. Mitochondrial CYPs are mostly involved in endogenous sterol metabolisms including biosynthesis of steroid hormones (such as estrogen), vitamin D3, and bile acids [160]. CYP1A1 and CYP3A4 are capable of metabolizing a variety of carcinogens, such as polycyclic aromatic hydrocarbons, heterocyclic amines, nitrosamines, azo-dyes, and alkylating agents [161,162], and by doing so may produce active derivatives that lead to the tumor initiation. Many CYPs, including CYP3A, are expressed in varieties of extrahepatic tissues such as digestive tract including cancer tissues [163]. Interestingly, the expression levels of *CYP1A1* and *CYP3A* in tumor and cirrhotic liver tissues are decreased in comparison with those in normal tissues [164,165], which is consistent with the signature we observed after BPA exposure. 

Taken together, the analyses of the miRNA’s targets revealed the same perturbed pathways and GO terms as the analysis of the entire DE dataset did and highlighted that BPA affects many gene programs related to mitochondrial function, energy metabolism, NAFLD, all of which are relevant for tumor and cancer development. Note, however, that Cell cycle was not as enriched in genes targeted by the miRNAs as it was in the analysis of the entire DE dataset. Nevertheless, our data show that the deregulation of the miRNA network is closely related to the overall signature of the BPA impacted hepatic transcriptome. Maybe this is not so surprising since 20–30% of all genes are miRNA targets [166] and miRNAs are estimated to comprise 1–5% of animal genes [125,167,168], making them one of the most abundant classes of regulators. This is further reinforced by their high degree of evolutionary conservation and by the many biological processes in which they are implicated, including developmental timing, cell proliferation, apoptosis, metabolism, cell differentiation, and morphogenesis [169,170].

The matrix of miRNAs we generated indicates that several genes are targeted by more than 1 miRNA, and in some instance more than 5 miRNAs are involved in the regulation of a particular gene. For example, the gene enzyme ecto-5′-nucleotidase is targeted by 10 different miRNAs that have no similarities in their seed sequence. Interestingly, this enzyme’s expression and enzymatic activity is downregulated in patients with alcoholic liver disease [171], suggesting that it is a key player in liver function and its downregulation leads to pathologies of the liver. The matrix allowed us to generate a similarity plot of miRNAs highlighting how similar miRNAs are based on the number of targets they possess in common. The concept of “cooperative regulation by miRNAs” is not new and co-regulation of common genes or biological processes by multiple miRNAs confers a powerful regulatory effect of the miRNA network. Here we show that dre-miR-724 and dre-miR-725 have many common target genes, including ecto-5′-nucleotidase. These 2 miRNAs originate from different chromosomes and their seed sequence share no similarities yet 73 common genes are predicted targets for silencing. In contrast, miRNAs that belong to the same family might not have as many common targets as we could anticipate, even though their seed sequence is exactly the same. This is the case of dre-miR-430a, -430b and -430c. Dre-miR-430a and dre-miR-133a have more targets in common than with any other miR-430 family members. To better understand the effect of the deregulated miRNAs on the modules of interest, we generated a network of miRNAs, targets and modules using Cytoscape (Figure 10). Here we showed that dre-miR-724 and -725 teamed up with dre-miR-193a, -202, -205 and -133a to regulate apoptosis, autophagy, NAFLD, oxidative phosphorylation and cell cycle modules whereas other miRNAs only had one target in a single module (dre-miR-184, -430a, -430b). Dre-miR-2189 was the only downregulated miRNA identified in this study after BPA exposure, yet alone this miRNA has numerous targets in apoptosis, autophagy, cell cycle and receptor modules. Taken together, these observations really emphasize the idea of “miRNA communities” that depends on the effect they have on downstream targets and pathways and not on seed similarities. 

### 4.4. Bisphenol A and Nuclear Hormone Receptors

BPA has changed our society with unforeseen impacts on human and ecosystem health due to its hormone-like properties [1,172]. Because its chemical structure resembles the estrogen Estradiol (E2), BPA acts as an endocrine disruptor. BPA has been shown to bind and activate the two estrogen receptors ERα and ERβ [16], causing multiple adverse outcomes related to the many physiological processes that ERs influence [173]: development or progression of numerous diseases such as breast/ovarian/colorectal/prostate/endometrial cancers, osteoporosis, neurodegenerative diseases, cardiovascular disease, insulin resistance, lupus erythematosus, endometriosis, and obesity. BPA also has ER-independent effects via activation of other receptors. BPA induces otolith malformations during vertebrate embryogenesis via activation of the orphan nuclear estrogen related receptor ERRγ [23,174]; in fact ERRγs have a stronger affinity to BPA than the ERs do by 40 fold. Additionally, BPA has been shown to bind and activate androgen receptors, thyroid hormone receptors, G-protein-coupled receptors (GPRs), glucocorticoid receptors and pregnane X receptor (PXR) [18,19,20,21,175]. We show here that BPA can also modulate the expression of several receptors (Figure 8, Receptors module), which is consistent with other studies in fish liver and gonads [176,177].

BPA, like other endocrine disruptors, can produce a non-monotonic dose response curve (NMDRC) [178], a response where the slope of the curve changes sign from positive to negative or vice versa along the range of doses examined [179]. Mechanisms that produce NMDRCs include cell-tissue specific receptors and cofactors, receptor selectivity, receptor competition and the ratio of receptor being produced over being degraded [180]. Here we found that 100 nM BPA exposure for 3 weeks regulated the expression of many receptors including estrogen receptor 1 (*esr1*), estrogen-related receptor gamma a (*esrrga*), parathyroid hormone 2 receptor (PTH2R), several GPRs and thyroid hormone receptor interactors (TRIPs) (Figure 8). Most of the genes in this module are upregulated, except 6 that are downregulated, including *esr1*. Given that the exposure we subjected these adult zebrafish to is on the high end of the spectrum of environmental exposures [4], it will be necessary to further investigate the role of BPA at lower dose on the regulation of these receptors and clearly define the nature of NMDRC in response to a spectrum of BPA exposures. This will be the subject of future work.

## 5. Conclusions

Many studies have established the link between BPA exposure and cancer development, yet the mechanisms and players involved in this switch from normal to carcinogenic phenotype in cells, ECM and tissue as a whole remain unclear. Epigenetic mechanisms, including miRNAs, are essential in setting up the necessary genomic instability that cancers require to develop and advance. MiRNA networks control the expression of hundreds of protein coding genes and modulate a wide spectrum of biological functions that are fundamental to tumorigenesis. Cancer cells are capable of hijacking miRNA biogenesis to promote dysregulation of miRNA networks. Here we showed that BPA perturbed the expression of several miRNAs and other epigenetic factors important for histone modifications and DNA methylation, as well as their downstream gene regulatory networks. Given that epigenetic alterations deregulate miRNA expression [181,182], this suggests that BPA could have carcinogenic effects partly by modifying epigenetic regulation of gene expression. This will be the focus of future work in our laboratory using zebrafish as toxicology model. Most studies to date have examined the effects of early exposure to BPA on embryonic development and on epigenetic transgenerational inheritance of adult onset disease in subsequent generations. In our study, we showed that short term BPA exposure modulated the adult male zebrafish hepatic transcriptome and miRNome, and that many biological pathways and GO terms related to cancer were perturbed as a result. 

Using zebrafish as a cancer model is a relatively new concept, first proposed about 10 years ago. This model has the potential to help scientists uncover relevant findings using techniques that may not be applicable in the traditional human and mouse systems commonly used by cancer biologists. High-quality chromatin immunoprecipitation followed by deep sequencing and methylation profiling in zebrafish are being developed and optimized [48], and will surely bring more insights into cancer biology and other pathologies. 

## Figures and Tables

**Table 1:** Significant deregulated miRNAs obtained from EdgeR analysis (*q* ≤ 0.1). All miRNAs listed here were upregulated in the liver after bisphenol A (BPA) exposure except miR-2189, the only miRNA that was downregulated (see log2FoldChange column). The Base Mean is a mean value for the counts obtained from the various samples.

Symbol	Base Mean	log2FoldChange	*p*-Value	padj
dre-miR-430c-3p	25	9.99	6.14 × 10^−12^	1.33 × 10^−9^
dre-miR-430b-3p	2007.5	5.75	8.72 × 10^−7^	9.46 × 10^−5^
dre-miR-202-5p	156	3.92	5.17 × 10^−6^	2.80 × 10^−4^
dre-miR-122	12,698	2.60	4.01 × 10^−6^	2.80 × 10^−4^
dre-miR-430a-3p	320.75	4.76	8.71 × 10^−6^	3.78 × 10^−4^
dre-miR-499-3p	35.5	3.15	4.10 × 10^−5^	1.48 × 10^−3^
dre-miR-2189	31.75	−2.34	1.03 × 10^−4^	2.78 × 10^−3^
dre-miR-184	703.75	2.06	9.77 × 10^−5^	2.78 × 10^−3^
dre-miR-499-5p	43	2.56	2.84 × 10^−4^	6.84 × 10^−3^
dre-miR-205-5p	824.5	1.68	1.19 × 10^−3^	0.026
dre-miR-133a-3p	854.25	2.72	1.89 × 10^−3^	0.032
dre-miR-724	122.75	2.51	1.95 × 10^−3^	0.032
dre-miR-458-3p	451	2.02	2.01 × 10^−3^	0.032
dre-miR-725-3p	355	1.55	2.09 × 10^−3^	0.032
dre-miR-193a-3p	18.75	1.52	6.64 × 10^−3^	0.096

**Table 2:** ToppFun functional enrichment analysis—Biological Pathways. The top 20 significant pathways are shown below. ToppFun analysis revealed several pathways that were significantly deregulated related to cell cycle, transcription-translation-elongation, viral infection, mitochondrial energy production and oxidative phosphorylation.

Name	*q*-Value
Cell Cycle	4.56 × 10^−37^
Cell Cycle, Mitotic	2.55 × 10^−31^
Influenza Viral RNA Transcription and Replication	6.93 × 10^−21^
Peptide chain elongation	7.61 × 10^−20^
Influenza Life Cycle	7.61 × 10^−20^
Eukaryotic Translation Elongation	9.02 × 10^−20^
Influenza Infection	1.04 × 10^−19^
Viral mRNA Translation	3.11 × 10^−19^
Selenocysteine synthesis	5.64 × 10^−19^
Infectious disease	7.96 × 10^−19^
Nonsense Mediated Decay (NMD) independent of the Exon Junction Complex (EJC)	2.10 × 10^−18^
Eukaryotic Translation Termination	7.30 × 10^−18^
Oxidative phosphorylation	7.53 × 10^−18^
SRP-dependent co-translational protein targeting to membrane	7.54 × 10^−18^
Respiratory electron transport, ATP synthesis by chemiosmotic coupling, and heat production by uncoupling proteins.	8.61 × 10^−18^
Major pathway of rRNA processing in the nucleolus and cytosol	1.42 × 10^−17^
Formation of a pool of free 40S subunits	5.58 × 10^−17^
Nonsense-Mediated Decay (NMD)	1.83 × 10^−16^
Nonsense Mediated Decay (NMD) enhanced by the Exon Junction Complex (EJC)	1.83 × 10^−16^
rRNA processing in the nucleus and cytosol	1.11 × 10^−15^

**Table 3:** ToppFun GO functional enrichment analysis—Biological Process (BP). The top 20 significant BP terms are shown below.

Name	*q*-Value
Cell cycle	1.94 × 10^−55^
Cell cycle process	3.83 × 10^−52^
Mitotic cell cycle	3.46 × 10^−43^
Mitotic cell cycle process	1.13 × 10^−41^
Chromosome organization	1.40 × 10^−33^
Nuclear division	1.52 × 10^−31^
Organelle fission	6.05 × 10^−29^
Mitotic cell cycle phase transition	2.98 × 10^−24^
Cell cycle phase transition	1.28 × 10^−23^
Chromosome segregation	2.37 × 10^−23^
Cell division	1.67 × 10^−21^
Cellular macromolecule catabolic process	1.67 × 10^−21^
Regulation of cell cycle	5.40 × 10^−21^
Cellular macromolecule localization	2.58 × 10^−20^
Viral transcription	4.33 × 10^−20^
Cellular protein localization	4.76 × 10^−20^
Nuclear chromosome segregation	1.10 × 10^−19^
RNA processing	1.35 × 10^−19^
Sister chromatid segregation	1.94 × 10^−19^
Macromolecule catabolic process	2.26 × 10^−19^

**Table 4:** ToppFun functional enrichment analysis—Molecular Function (MF). The top 20 significant MF terms are shown below.

Name	*q*-Value
RNA binding	1.75 × 10^−28^
Macromolecular complex binding	1.17 × 10^−15^
NADH dehydrogenase (ubiquinone) activity	1.02 × 10^−12^
NADH dehydrogenase activity	1.02 × 10^−12^
NADH dehydrogenase (quinone) activity	1.02 × 10^−12^
Enzyme binding	1.81 × 10^−12^
Cytoskeletal protein binding	2.49 × 10^−12^
ATPase activity	1.92 × 10^−11^
Helicase activity	3.39 × 10^−11^
Chromatin binding	9.85 × 10^−11^
Oxidoreductase activity, acting on NAD(P)H, quinone or similar compound as acceptor	6.83 × 10^−10^
ATP binding	3.83 × 10^−09^
Nucleoside-triphosphatase activity	1.06 × 10^−08^
Tubulin binding	1.68 × 10^−08^
Protein complex binding	1.95 × 10^−08^
Microtubule binding	2.55 × 10^−08^
Adenyl ribonucleotide binding	2.95 × 10^−08^
Adenyl nucleotide binding	2.95 × 10^−08^
Pyrophosphatase activity	6.67 × 10^−08^
DNA helicase activity	7.01 × 10^−08^

**Table 5:** ToppFun functional enrichment analysis—Cellular Component (CC). The top 20 significant CC terms are shown below.

Name	*q*-Value
Chromosome	1.08 × 10^−31^
Chromosomal part	7.38 × 10^−27^
Microtubule cytoskeleton	2.34 × 10^−23^
Nucleolus	2.20 × 10^−22^
Nucleoplasm part	6.66 × 10^−21^
Catalytic complex	1.80 × 10^−20^
Microtubule organizing center	4.67 × 10^−18^
Respiratory chain	3.43 × 10^−17^
Cytosolic ribosome	3.43 × 10^−17^
Centrosome	3.49 × 10^−17^
Cytoskeletal part	1.12 × 10^−16^
Envelope	1.28 × 10^−15^
Chromosome, centromeric region	1.28 × 10^−15^
Chromosomal region	1.29 × 10^−15^
Ribonucleoprotein complex	1.29 × 10^−15^
Intracellular ribonucleoprotein complex	1.29 × 10^−15^
Organelle envelope	1.32 × 10^−15^
Respiratory chain complex	1.78 × 10^−15^
Spindle	3.31 × 10^−15^
Nuclear chromosome	1.04 × 10^−14^

## Supplementary Materials

The following are available online at www.mdpi.com/2073-4425/8/10/269/s1. Figure S1: Comparison of human and zebrafish annotations; Figure S2: Advaita-iPathwayGuide volcano plot; Figure S3: Oxidative phosphorylation (KEGG: 00190); Figure S4: Non-alcoholic fatty liver disease (NAFLD) (KEGG:04932); Figure S5: Cell cycle (KEGG:04110); Figure S6: Fanconia anemia (KEGG:03460); Figure S7: Metabolic pathways (KEGG:01100); Figure S8: Advaita-iPathwayGuide plots; Figure S9: Adherens junction (KEGG: 04520); Figure S10: Chemical carcinogenesis; Figure S11: Ribosome (KEGG: 03010); Table S1: (Matrix) is a separate pdf file that can be found in supplemental materials section; Table S2: Merge of the miRNA targets with the genes selected for each modules; Table S3: Expression levels of vitellogenin (VTG) and zona pellucida (ZP) genes in the liver of exposed zebrafish; Table S4: Advaita-iPathwayGuide analysis — Pathways; Table S5: Advaita-iPathwayGuide analysis — Biological Process; Table S6: Advaita-iPathwayGuide analysis — Molecular Function; Table S7: Advaita-iPathwayGuide analysis — Cellular Component; Table S8: ToppFun functional enrichment analysis of the mRNAs that are predicted targets of miRNAs of interest — Pathways; Table S9: ToppFun functional enrichment analysis of the mRNAs that are predicted targets of miRNAs of interest — Biological Process; Table S10: ToppFun functional enrichment analysis of the mRNAs that are predicted targets of miRNAs of interest — Molecular Function; Table S11: ToppFun functional enrichment analysis of the mRNAs that are predicted targets of miRNAs of interest — Cellular Component; Table S12: Advaita-iPathwayGuide analysis of all DE genes that are predicted targets of miRNAs of interest — Pathways; Table S13: Advaita-iPathwayGuide analysis of all DE genes that are predicted targets of miRNAs of interest — Cellular Component.
