# Supplementary material for: The Plasticizer Bisphenol A Perturbs the Hepatic Epigenome: A Systems Level Analysis of the miRNome
Source: Genes (Basel). 2017 Oct 13;8(10):269. doi: 10.3390/genes8100269 (PMC5664119; doi:10.3390/genes8100269)
Supplement: Supplementary file 1 [file genes-08-00269-s001.zip › zip_GENES_supplemental materials/__Supplemental Figures.pdf]

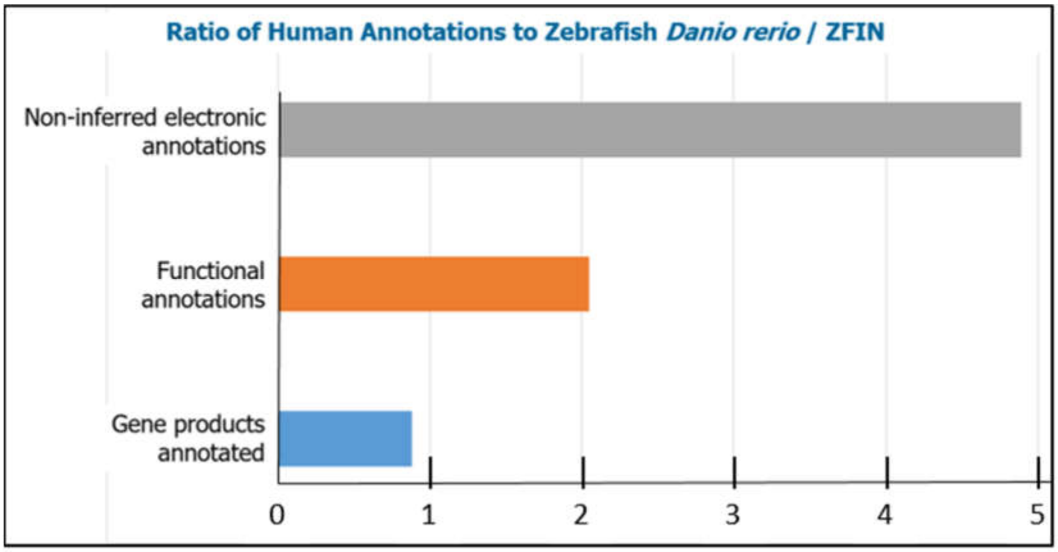

Figure S1: Comparison of human and zebrafish annotations. The ratio of human:zebrafish for non-inferred electronic, functional and annotated gene products are shown here (x-axis). In humans, non-inferred electronic (grey bar) and functional annotations (orange bar) are 5 times and 2 times better defined respectively than they are in zebrafish. In zebrafish, the number of annotated gene products is slightly better than for humans representing the ancient whole genome duplication event in the zebrafish lineage (blue bar).

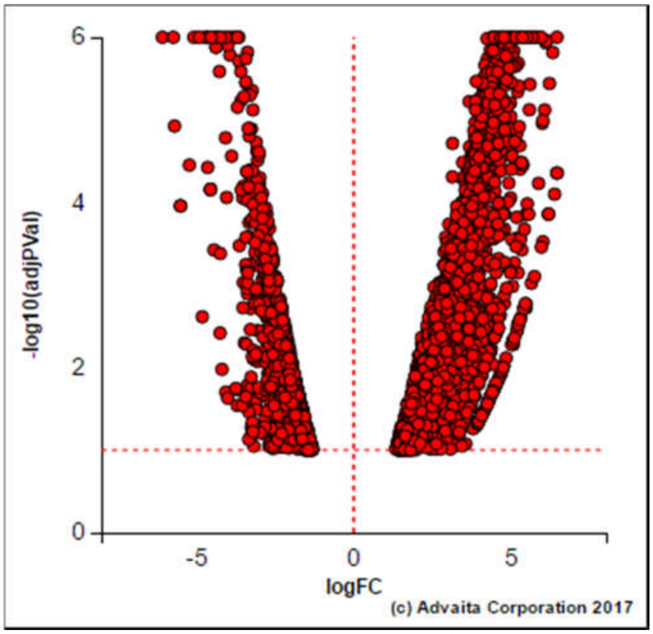

Figure S2: Advaita-iPathwayGuide volcano plot. All 4,341 DE human orthologs are represented in terms of their measured expression change (x-axis) and the significance of the change (y-axis). The significance is represented in terms of the negative log (base 10) of the p-value, so that more significant genes are plotted higher on the y-axis. The dotted lines represent the thresholds used to select the DE genes.

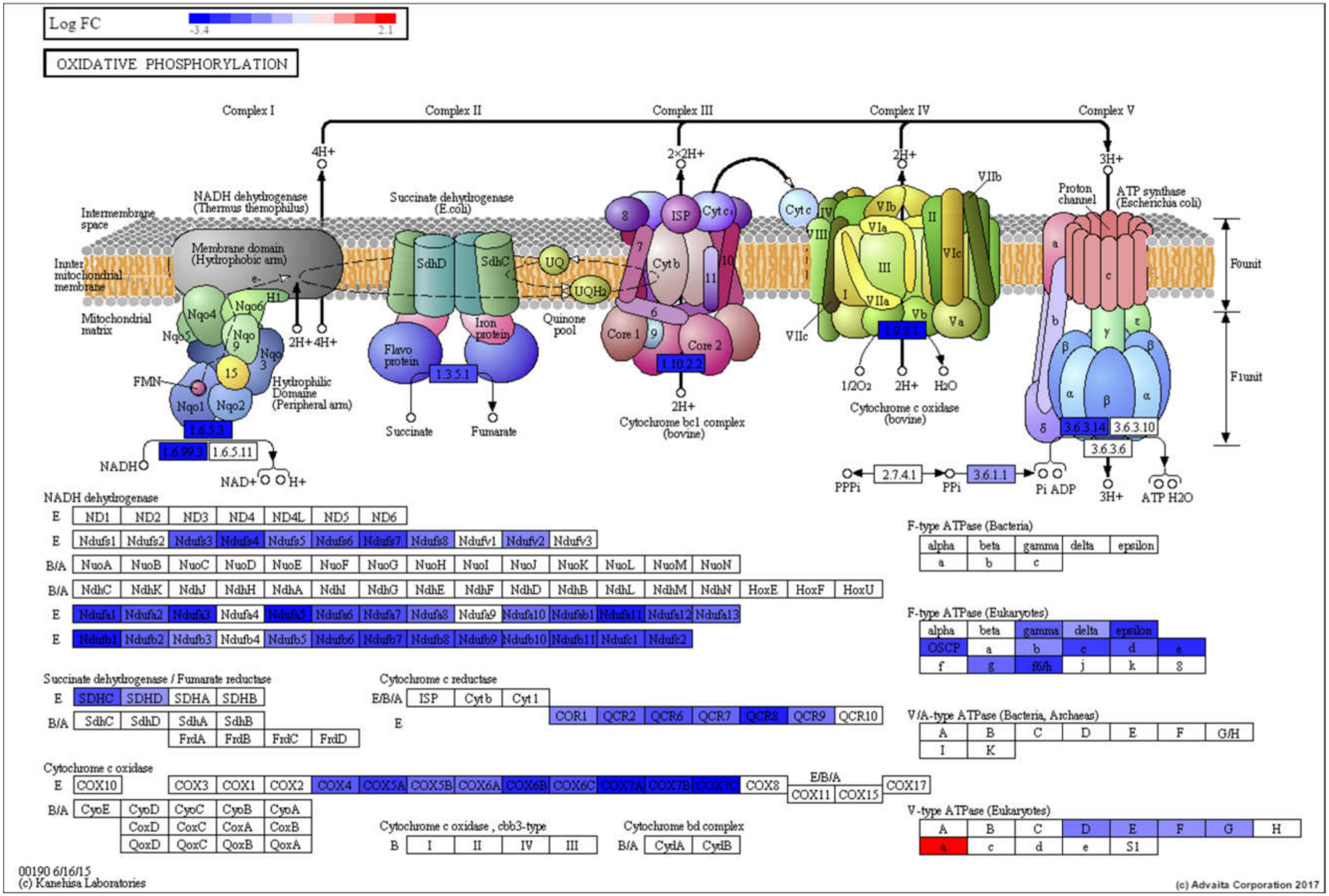

Figure S3: Oxidative phosphorylation (KEGG: 00190). The pathway diagram is overlayed with the computed perturbation of each gene. The perturbation accounts both for the gene's measured fold change and for the accumulated perturbation propagated from any upstream genes (accumulation). The highest negative perturbation is shown in dark blue, while the highest positive perturbation in dark red. The legend describes the values on the gradient. Note: For legibility, one gene may be represented in multiple places in the diagram and one box may represent multiple genes in the same gene family. A gene is highlighted in all locations it occurs in the diagram. For each gene family, the color corresponding to the gene with the highest absolute perturbation is displayed.

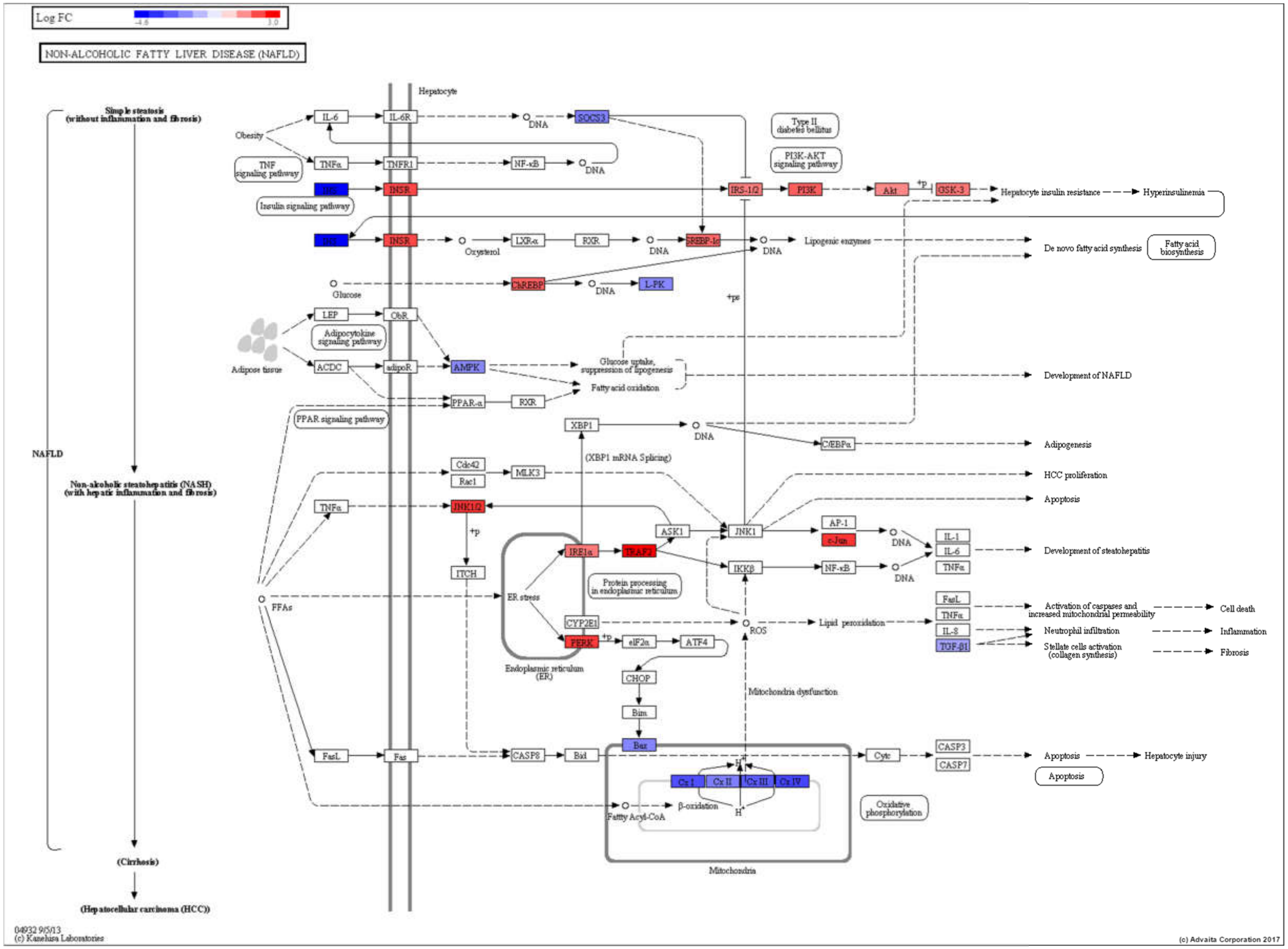

Figure S4: Non-alcoholic fatty liver disease (NAFLD) (KEGG:04932). The pathway diagram is overlayed with the computed perturbation of each gene. The perturbation accounts both for the gene's measured fold change and for the accumulated perturbation propagated from any upstream genes (accumulation). The highest negative perturbation is shown in dark blue, while the highest positive perturbation in dark red. The legend describes the values on the gradient. Note: For legibility, one gene may be represented in multiple places in the diagram and one box may represent multiple genes in the same gene family. A gene is highlighted in all locations it occurs in the diagram. For each gene family, the color corresponding to the gene with the highest absolute perturbation is displayed.

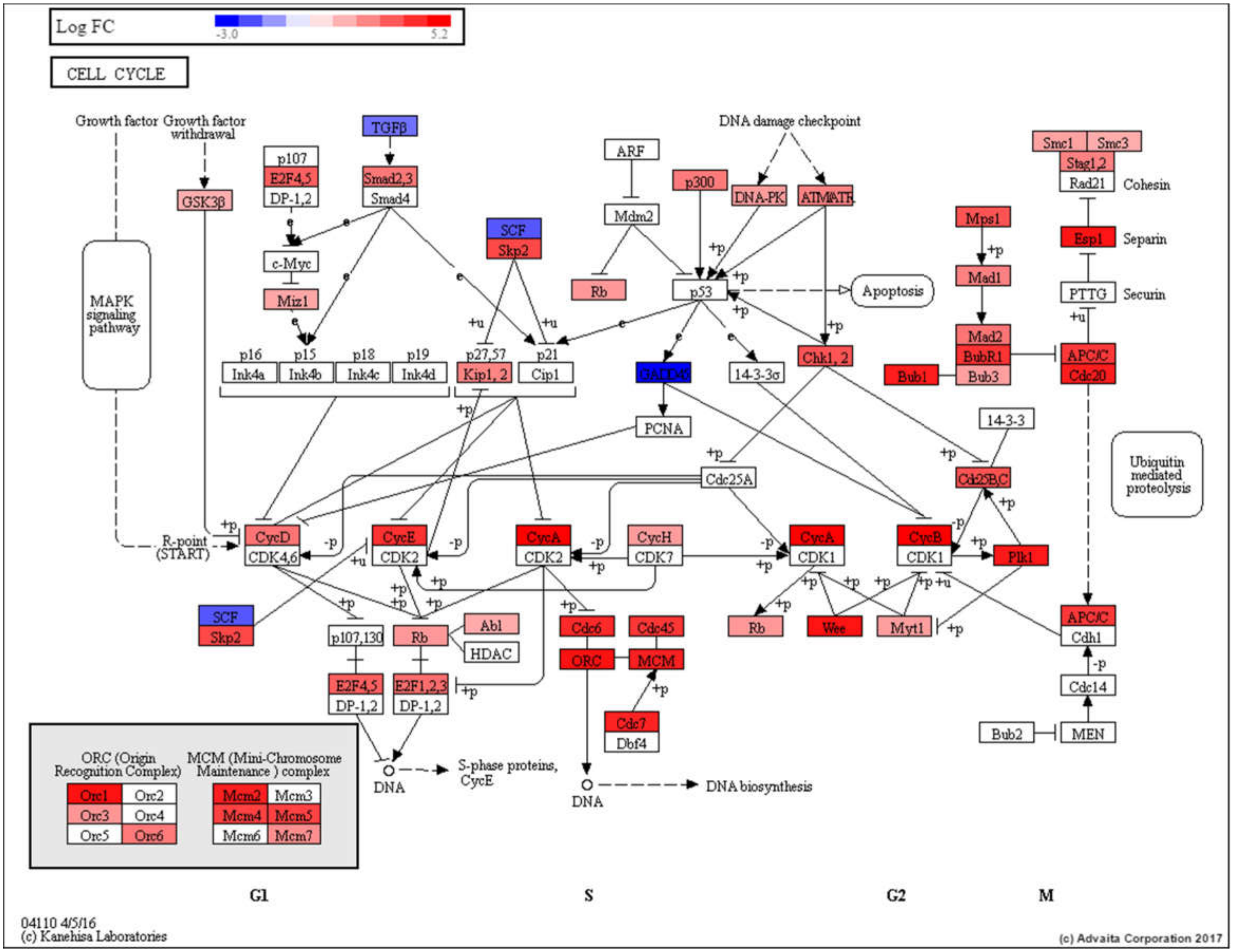

Figure S5: Cell cycle (KEGG:04110). The pathway diagram is overlayed with the computed perturbation of each gene. The perturbation accounts both for the gene's measured fold change and for the accumulated perturbation propagated from any upstream genes (accumulation). The highest negative perturbation is shown in dark blue, while the highest positive perturbation in dark red. The legend describes the values on the gradient. Note: For legibility, one gene may be represented in multiple places in the diagram and one box may represent multiple genes in the same gene family. A gene is highlighted in all locations it occurs in the diagram. For each gene family, the color corresponding to the gene with the highest absolute perturbation is displayed.

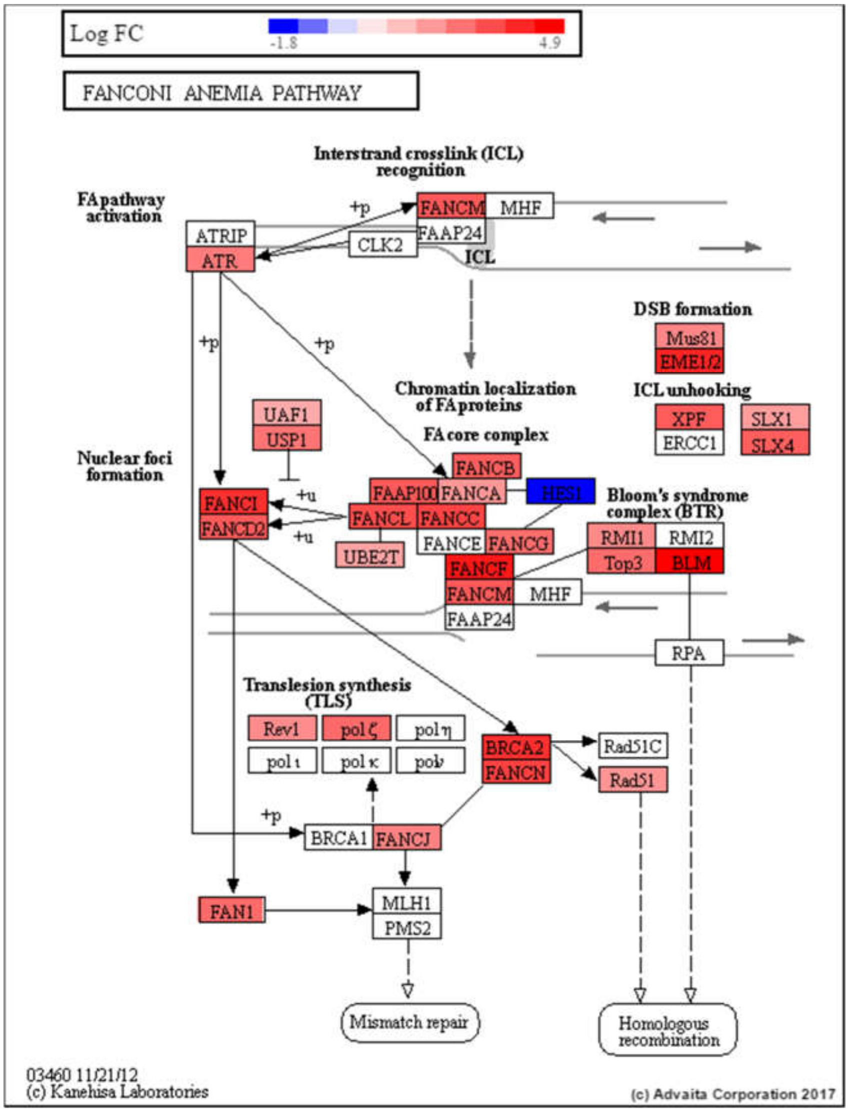

Figure S6: Fanconia anemia (KEGG:03460). The pathway diagram is overlayed with the computed perturbation of each gene. The perturbation accounts both for the gene's measured fold change and for the accumulated perturbation propagated from any upstream genes (accumulation). The highest negative perturbation is shown in dark blue, while the highest positive perturbation in dark red. The legend describes the values on the gradient. Note: For legibility, one gene may be represented in multiple places in the diagram and one box may represent multiple genes in the same gene family. A gene is highlighted in all locations it occurs in the diagram. For each gene family, the color corresponding to the gene with the highest absolute perturbation is displayed.

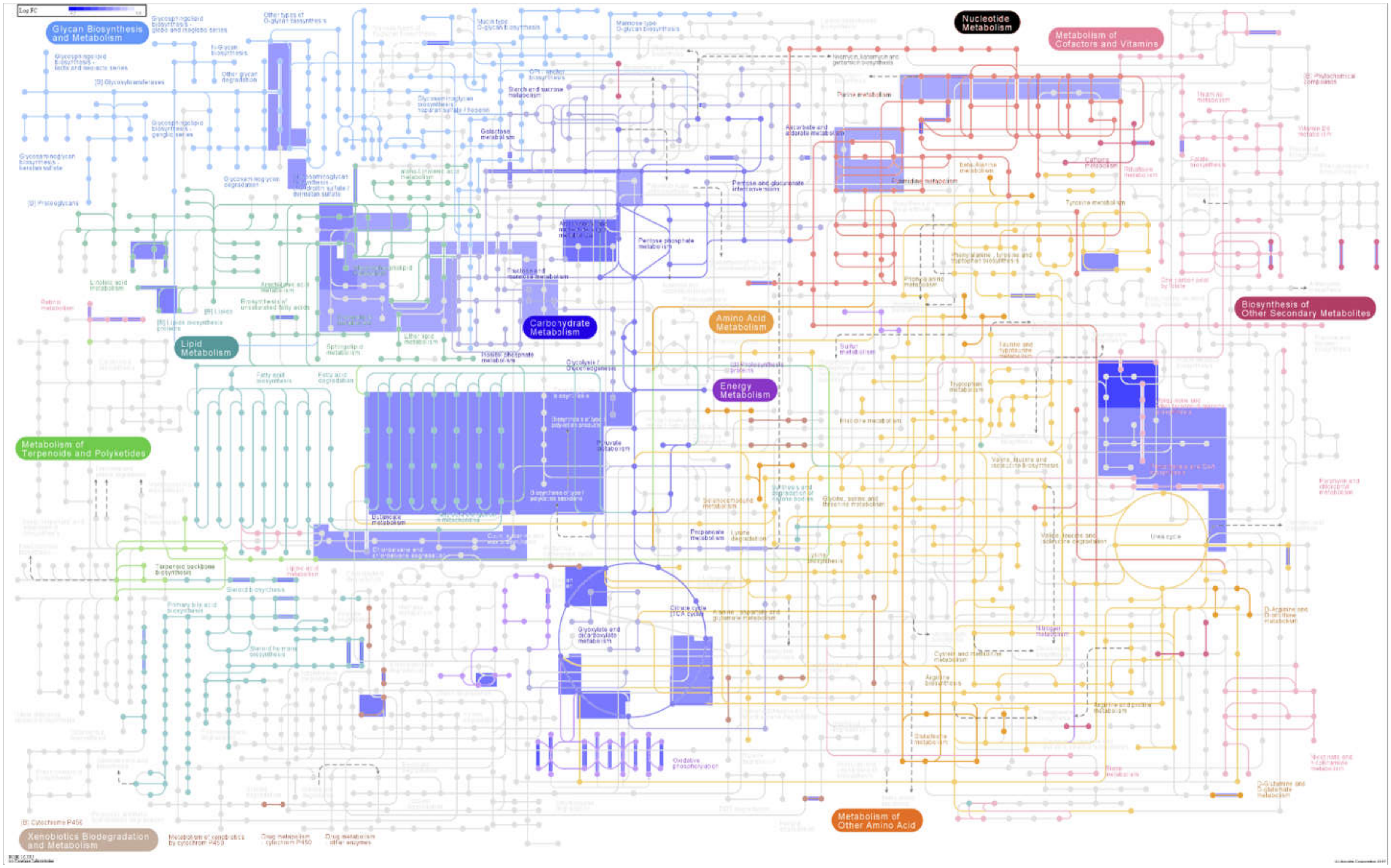

Figure S7: Metabolic pathways (KEGG:01100). The pathway diagram is overlayed with the computed perturbation of each gene. The perturbation accounts both for the gene's measured fold change and for the accumulated perturbation propagated from any upstream genes (accumulation). The highest negative perturbation is shown in dark blue, while the highest positive perturbation in dark red. The legend describes the values on the gradient. Note: For legibility, one gene may be represented in multiple places in the diagram and one box may represent multiple genes in the same gene family. A gene is highlighted in all locations it occurs in the diagram. For each gene family, the color corresponding to the gene with the highest absolute perturbation is displayed.

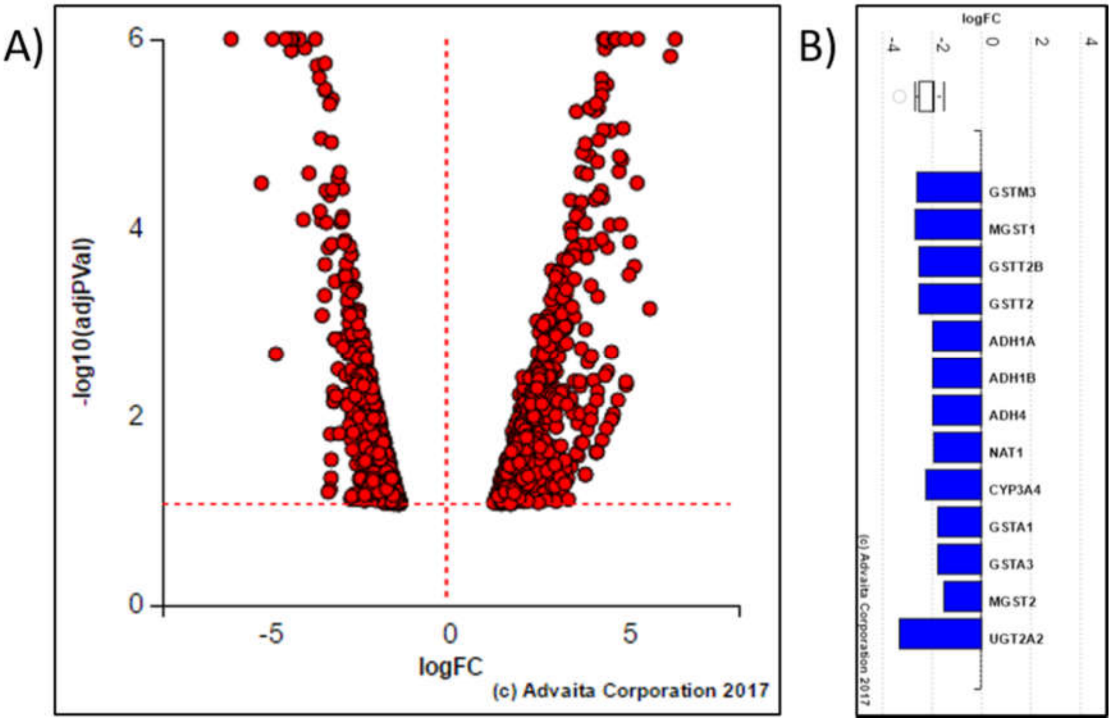

Figure S8: Advaita-iPathwayGuide plots. **(A)** Volcano plot showing all 1,211 significantly differentially expressed (DE) human orthologs ($q \leq 0.1$); genes are represented in terms of their measured expression change (x-axis) and the significance of the change (y-axis). The significance is represented in terms of the negative log (base 10) of the p-value, so that more significant genes are plotted higher on the y-axis. The dotted lines represent the thresholds used to select the DE genes. **(B)** Barplot of the genes belonging to the Chemical carcinogenesis pathway. All the genes are ranked based on their log 2 fold change (FC). For each gene, the FC is represented with negative values in blue. The box and whisker plot on the top summarizes the distribution of all gene expression in this pathway. The box represents the 1st quartile, the median and the 3rd quartile, while the outliers are represented by circles.

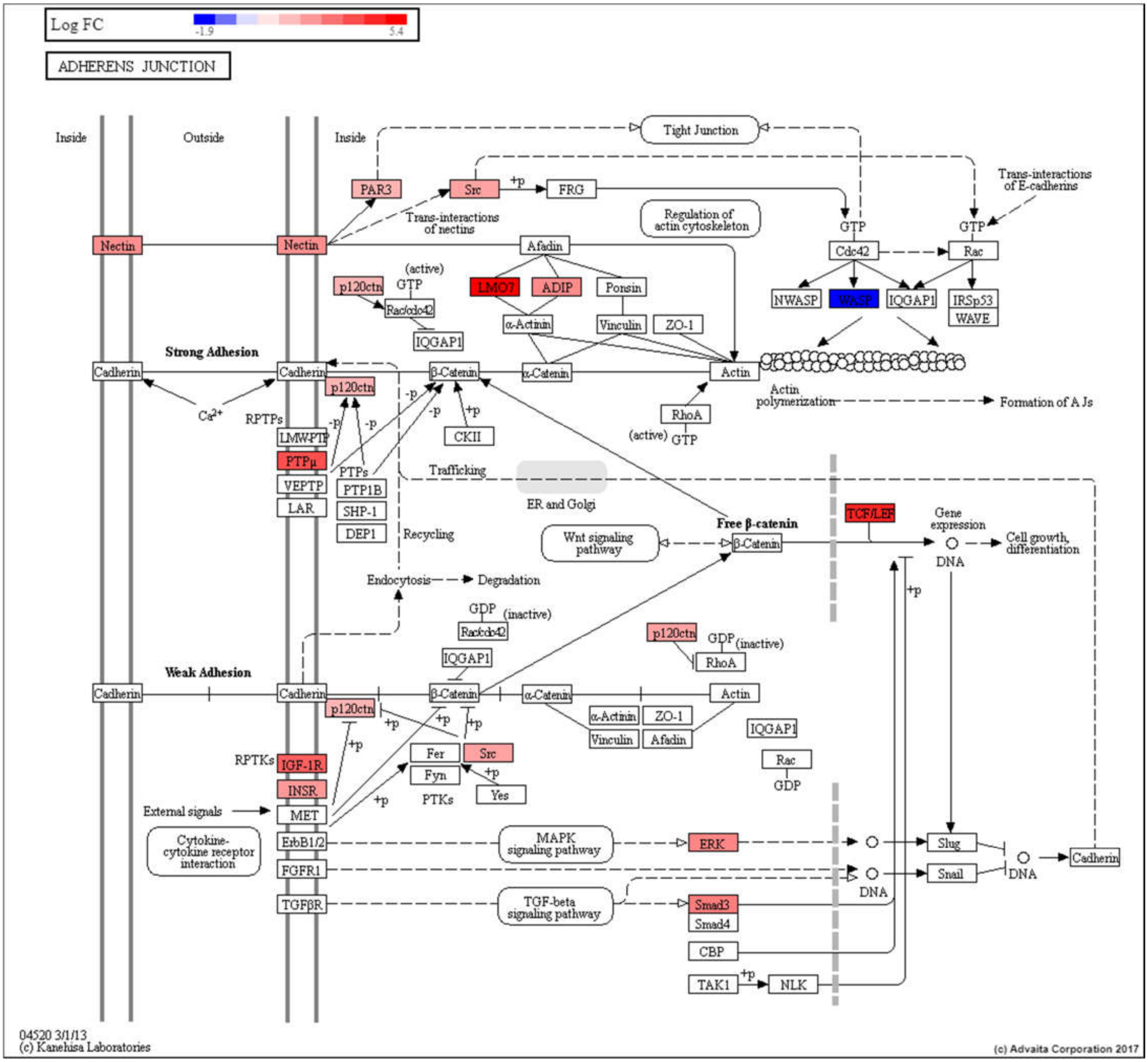

Figure S9: Adherens junction (KEGG: 04520). The pathway diagram is overlayed with the computed perturbation of each gene. The perturbation accounts both for the gene's measured fold change and for the accumulated perturbation propagated from any upstream genes (accumulation). The highest negative perturbation is shown in dark blue, while the highest positive perturbation in dark red. The legend describes the values on the gradient. Note: For legibility, one gene may be represented in multiple places in the diagram and one box may represent multiple genes in the same gene family. A gene is highlighted in all locations it occurs in the diagram. For each gene family, the color corresponding to the gene with the highest absolute perturbation is displayed.

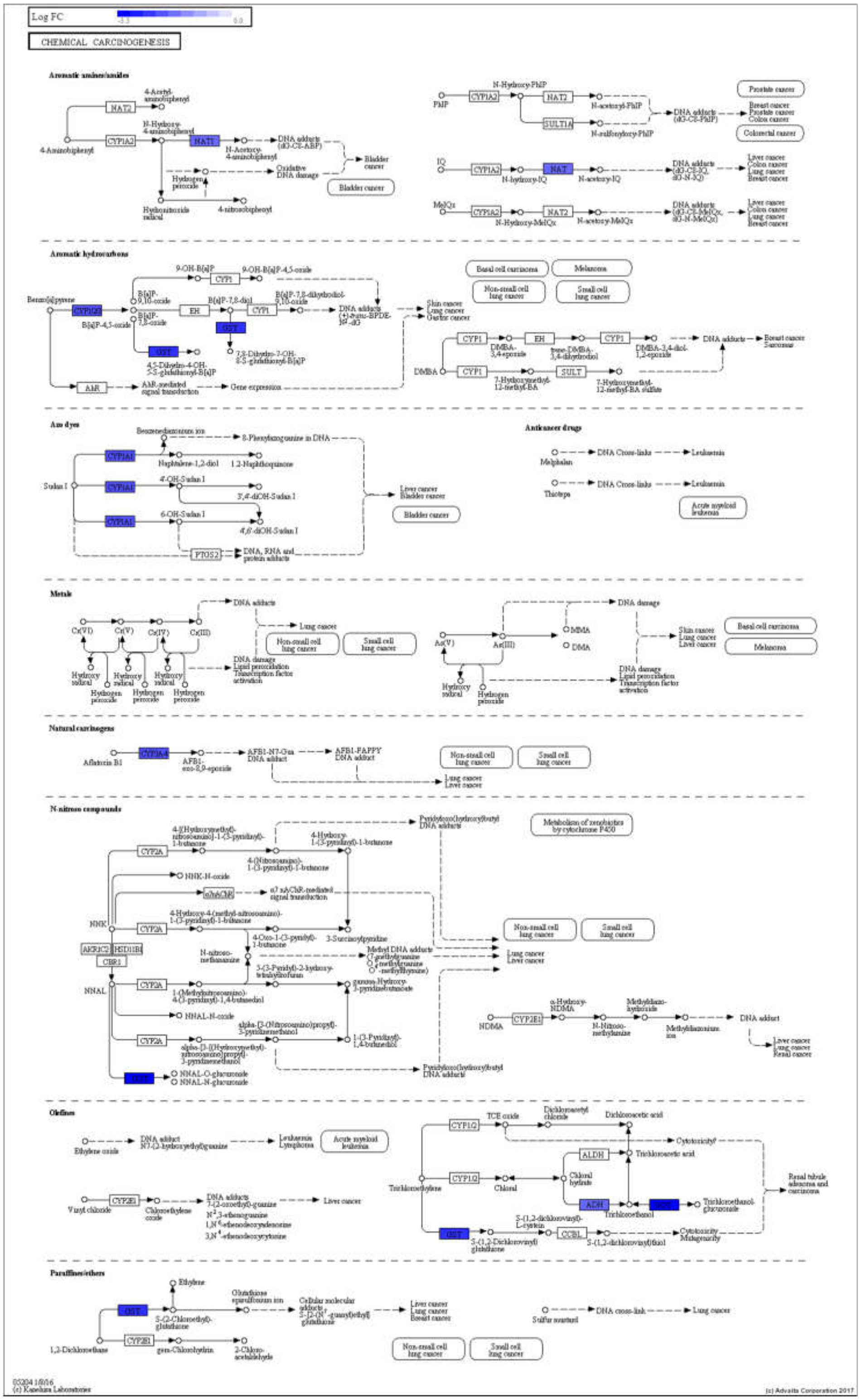

Figure S10: Chemical carcinogenesis. The pathway diagram is overlayed with the computed perturbation of each gene. The perturbation accounts both for the gene's measured fold change and for the accumulated perturbation propagated from any upstream genes (accumulation). The highest negative perturbation is shown in dark blue, while the highest positive perturbation in dark red. The legend describes the values on the gradient. Note: For legibility, one gene may be represented in multiple places in the diagram and one box may represent multiple genes in the same gene family. A gene is highlighted in all locations it occurs in the diagram. For each gene family, the color corresponding to the gene with the highest absolute perturbation is displayed.

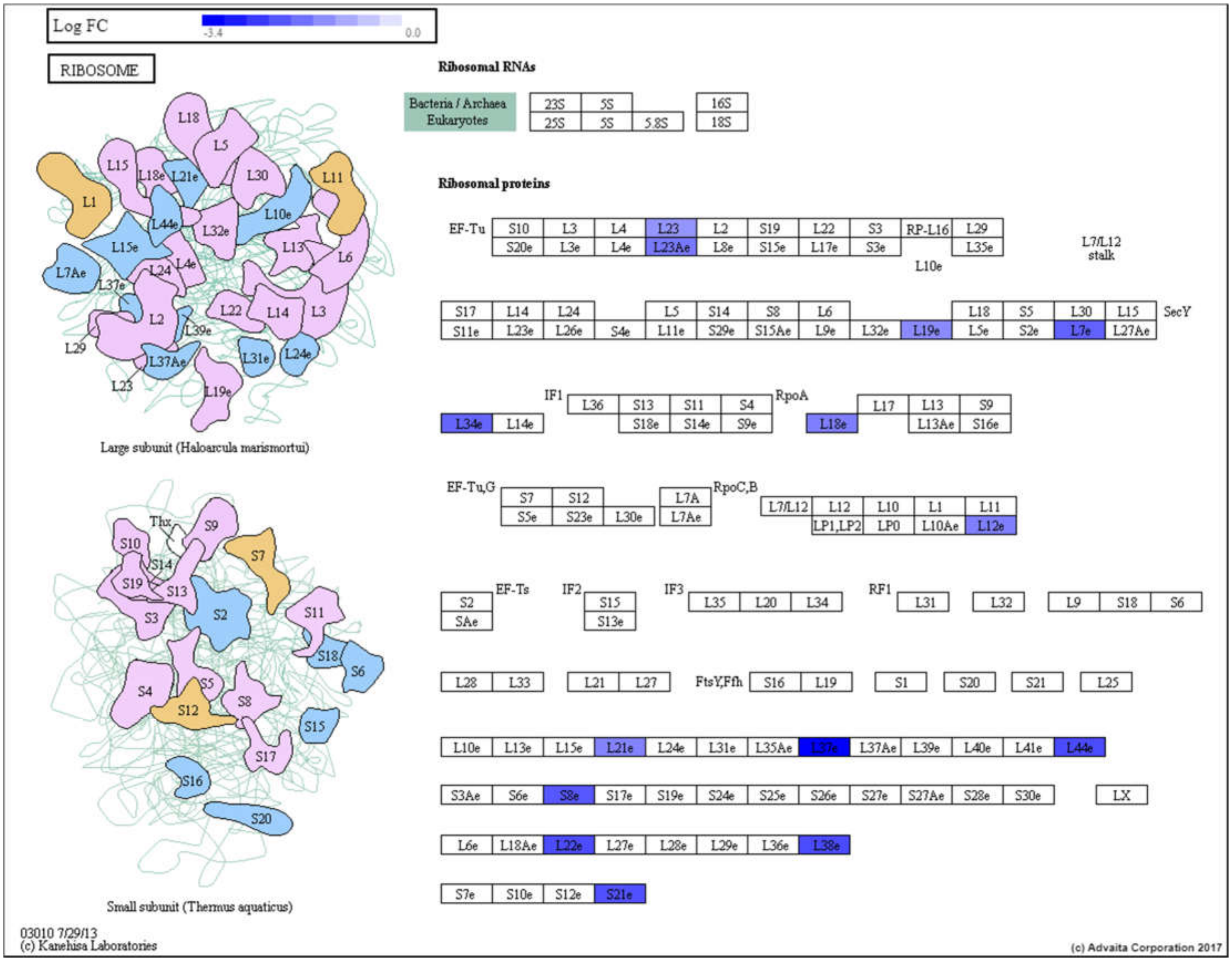

Figure S11: Ribosome (KEGG: 03010). The pathway diagram is overlayed with the computed perturbation of each gene. The perturbation accounts both for the gene's measured fold change and for the accumulated perturbation propagated from any upstream genes (accumulation). The highest negative perturbation is shown in dark blue, while the highest positive perturbation in dark red. The legend describes the values on the gradient. Note: For legibility, one gene may be represented in multiple places in the diagram and one box may represent multiple genes in the same gene family. A gene is highlighted in all locations it occurs in the diagram. For each gene family, the color corresponding to the gene with the highest absolute perturbation is displayed.
