# Supplementary material for: The Plasticizer Bisphenol A Perturbs the Hepatic Epigenome: A Systems Level Analysis of the miRNome
Source: Genes (Basel). 2017 Oct 13;8(10):269. doi: 10.3390/genes8100269 (PMC5664119; doi:10.3390/genes8100269)
Supplement: Supplementary file 1 [file genes-08-00269-s001.zip › zip_GENES_supplemental materials/__Supplemental Table S1_matrix.pdf]

gene symbol	dre-miR-2189	dre-miR-499	dre-miR-724	dre-miR-430a	dre-miR-202	dre-miR-725	dre-miR-205	dre-miR-133a	dre-miR-430b	dre-miR-122	dre-miR-430c	dre-miR-458	dre-miR-193a	dre-miR-184	sum miRNAs
nt5e	0	1	1	1	1	1	1	1	0	1	0	1	1	0	10
lin52	0	1	1	1	0	1	1	1	0	1	0	0	0	1	8
sowaha	0	1	1	0	1	1	1	1	1	0	0	0	1	0	8
mrpl23	0	1	1	0	1	1	0	1	0	0	0	1	1	0	7
dfna5b	0	0	1	1	1	1	0	1	0	0	0	1	1	0	7
si:dkey-19a16.4	0	0	1	1	0	1	1	1	0	1	0	0	1	0	7
hdhd1	0	1	1	0	1	0	1	1	1	0	0	0	1	0	7
tuft1b	0	0	1	1	1	0	1	1	0	0	0	1	0	1	7
upb1	0	1	1	0	0	1	1	0	0	0	0	1	1	0	6
pcbd1	0	1	1	0	1	1	1	0	0	0	0	1	0	0	6
tmem35	0	1	1	1	0	1	1	0	0	0	0	1	0	0	6
adap1	0	1	1	0	0	1	0	1	0	1	1	0	0	0	6
lyplal1	0	1	0	0	1	1	1	0	0	0	0	1	1	0	6
ndufb9	0	1	1	0	1	1	0	0	0	1	0	0	0	0	5
gstt1b	0	1	1	0	1	1	0	0	0	0	0	0	1	0	5
bcap29	0	1	1	0	1	1	0	0	0	1	0	0	0	0	5
zbtb8os	0	1	1	0	0	1	1	0	0	0	0	0	0	1	5
derl1	0	1	1	0	1	1	0	0	0	1	0	0	0	0	5
add2	0	0	1	0	0	1	1	1	0	0	1	0	0	0	5
pla2g12a	0	0	1	0	0	1	1	0	1	0	0	1	0	0	5
gypc	0	0	1	0	1	1	0	0	0	1	0	1	0	0	5
hsd17b12b	0	0	1	0	1	1	1	0	0	0	1	0	0	0	5
nkiras2	0	0	1	0	0	1	0	1	1	0	0	1	0	0	5
cmklr1	0	1	0	1	1	1	1	0	0	0	0	0	0	0	5
mecr	0	1	0	0	0	1	0	0	1	1	0	1	0	0	5
msmo1	0	0	0	1	1	1	1	0	0	0	0	0	1	0	5
ssuh2.3	0	0	0	0	0	1	0	1	1	1	0	1	0	0	5
alas2	0	1	1	0	1	0	1	0	0	0	0	0	1	0	5
manbal	0	1	1	0	0	0	0	0	1	1	0	1	0	0	5
raver2	0	1	1	1	0	0	0	0	0	0	0	1	1	0	5
pcmt	0	1	1	0	0	0	1	0	0	0	0	0	1	1	5
rdh8a	0	1	1	1	0	0	1	0	0	0	0	0	0	1	5
ubald1b	0	1	1	0	0	0	1	0	1	1	0	0	0	0	5
hck	0	1	1	0	0	0	1	1	0	0	0	0	1	0	5
cox19	0	1	1	0	0	1	0	0	0	0	0	1	0	0	4
il1fma	0	1	1	0	0	1	1	0	0	0	0	0	0	0	4
rps8b	0	1	1	1	0	1	0	0	0	0	0	0	0	0	4
ncf2	0	1	1	0	0	1	0	0	0	1	0	0	0	0	4
sdr16c5b	0	1	1	1	0	1	0	0	0	0	0	0	0	0	4
ndufs4	0	0	1	0	0	1	1	1	0	0	0	0	0	0	4
pycard	0	0	1	0	0	1	0	0	0	0	0	1	1	0	4
rhogb	0	0	1	0	0	1	0	0	0	1	0	1	0	0	4
arrdc3a	0	0	1	0	0	1	1	0	1	0	0	0	0	0	4
romo1	0	0	1	0	0	1	1	0	0	0	1	0	0	0	4
tmem71	0	0	1	1	0	1	0	1	0	0	0	0	0	0	4
cd81a	0	0	1	0	1	1	1	0	0	0	0	0	0	0	4
ptmaa	0	0	1	0	0	1	1	0	0	0	0	0	1	0	4
pmp22b	0	0	1	0	0	1	1	0	1	0	0	0	0	0	4
tcf21	0	0	1	0	0	1	0	1	0	0	1	0	0	0	4
ptprh	0	0	1	0	1	1	0	0	0	0	0	1	0	0	4
ciapin1	0	0	1	0	0	1	0	1	0	0	0	1	0	0	4
calca	0	0	1	0	1	1	0	0	0	1	0	0	0	0	4
etnppl	0	0	1	0	0	1	0	0	0	0	1	1	0	0	4
asah1b	0	1	0	0	0	1	0	1	0	0	1	0	0	0	4
gnpnat1	0	1	0	0	0	1	1	0	0	0	0	0	1	0	4
zbtb16b	0	1	0	0	0	1	1	0	0	1	0	0	0	0	4
zgc:172090	0	0	0	1	1	1	0	1	0	0	0	0	0	0	4
cxcl12b	0	0	0	1	0	1	1	1	0	0	0	0	0	0	4
slc38a5a	0	0	0	1	0	1	0	1	0	1	0	0	0	0	4
cav2	0	0	0	0	1	1	1	0	0	0	0	0	1	0	4
c18h15orf48	0	1	1	0	0	0	1	0	0	0	0	0	1	0	4
tnfrsf14	0	1	1	0	1	0	0	0	0	0	0	1	0	0	4
zgc:195173	0	1	1	1	0	0	1	0	0	0	0	0	0	0	4
trpt1	0	1	1	1	0	0	1	0	0	0	0	0	0	0	4
psmg2	0	1	1	0	0	0	1	0	0	0	0	1	0	0	4
yipf2	0	1	1	0	0	0	1	0	0	0	0	1	0	0	4
triqk	0	1	1	0	1	0	0	0	0	0	0	1	0	0	4
sirt5	0	1	1	0	0	0	0	0	0	1	0	0	1	0	4
efhd2	0	1	1	0	1	0	0	1	0	0	0	0	0	0	4
uqcc2	0	1	1	0	0	0	0	1	0	0	0	1	0	0	4
calm3b	0	1	1	0	0	0	1	1	0	0	0	0	0	0	4
cxcl12a	0	0	1	1	0	0	0	1	0	1	0	0	0	0	4
cr762483.1	0	0	1	0	1	0	1	0	0	0	0	1	0	0	4
si:dkey-237j10.2	0	0	1	0	1	0	0	0	0	0	0	1	1	0	4
atp5s	0	0	1	1	1	0	0	0	0	1	0	0	0	0	4
maf1	0	0	1	1	1	0	0	0	0	1	0	0	0	0	4
pebp1	0	0	1	1	0	0	0	1	0	0	0	1	0	0	4
fam46ba	0	0	1	0	1	0	0	1	0	1	0	0	0	0	4
mpdu1b	0	0	1	0	0	0	1	1	0	0	0	0	1	0	4
hsdl2	0	0	1	0	0	0	0	1	0	0	0	1	1	0	4
si:ch211-66k16.27	0	1	0	0	0	0	1	1	0	1	0	0	0	0	4
elovl6	0	1	0	0	1	0	1	0	0	0	0	1	0	0	4
prkab1a	0	1	0	1	0	0	0	1	0	0	0	1	0	0	4
ankrd39	0	1	0	1	0	0	0	0	0	0	0	1	1	0	4

cx30.3	0	1	0	0	0	0	1	0	0	0	0	1	0	1	4
tmem126a	0	1	0	1	1	0	0	0	0	1	0	0	0	0	4
mea1	0	1	0	0	0	0	1	1	0	0	0	1	0	0	4
haao	0	1	0	0	1	0	0	1	0	1	0	0	0	0	4
metap2a	0	1	0	0	0	0	1	0	0	1	0	0	1	0	4
elovl2	0	0	0	1	0	0	1	1	0	1	0	0	0	0	4
ckmt1	0	0	0	1	1	0	0	1	0	1	0	0	0	0	4
agpat9l	0	0	0	0	1	0	1	0	0	0	0	1	1	0	4
pxmp2	0	0	0	0	1	0	1	0	1	0	0	1	0	0	4
serf2	0	1	1	0	0	1	0	0	0	0	0	0	0	0	3
ptger2b	0	1	1	0	0	1	0	0	0	0	0	0	0	0	3
clic2	0	0	1	0	0	1	1	0	0	0	0	0	0	0	3
ramp2	0	0	1	0	0	1	1	0	0	0	0	0	0	0	3
myl9a	0	0	1	0	0	1	0	0	0	0	0	0	1	0	3
uqcrh	0	0	1	0	0	1	0	0	0	0	0	0	1	0	3
idi1	0	0	1	0	0	1	0	0	0	1	0	0	0	0	3
zgc:194659	0	0	1	0	0	1	0	0	0	0	0	1	0	0	3
creld1	0	0	1	0	1	1	0	0	0	0	0	0	0	0	3
lgals3l	0	0	1	0	0	1	0	0	0	0	0	0	1	0	3
si:ch211-245h14.1	0	0	1	0	0	1	0	0	0	1	0	0	0	0	3
si:ch211-193e13.5	0	0	1	0	1	1	0	0	0	0	0	0	0	0	3
si:ch211-217k17.11	0	0	1	1	0	1	0	0	0	0	0	0	0	0	3
fam96b	0	0	1	0	1	1	0	0	0	0	0	0	0	0	3
ablim1a	0	0	1	0	0	1	0	1	0	0	0	0	0	0	3
stom	0	0	1	0	0	1	1	0	0	0	0	0	0	0	3
kcnn4	0	0	1	0	0	1	0	0	0	1	0	0	0	0	3
nudt7	0	0	1	0	0	1	0	0	0	0	0	1	0	0	3
tmem45a	0	0	1	0	0	1	0	1	0	0	0	0	0	0	3
mylpfa	0	1	0	0	0	1	0	0	1	0	0	0	0	0	3
ucp3	0	1	0	0	0	1	0	0	0	0	0	0	1	0	3
derl3	0	1	0	0	0	1	1	0	0	0	0	0	0	0	3
ccr12b.2	0	1	0	0	0	1	0	0	0	0	0	1	0	0	3
zgc:77517	0	1	0	0	0	1	0	0	0	1	0	0	0	0	3
fam162a	0	1	0	0	0	1	0	0	1	0	0	0	0	0	3
si:rp71-36a1.5	0	1	0	0	0	1	0	0	0	0	0	0	1	0	3
cited4a	0	1	0	0	0	1	1	0	0	0	0	0	0	0	3
mapk11	0	1	0	0	0	1	0	1	0	0	0	0	0	0	3
nr2e1	0	0	0	1	0	1	1	0	0	0	0	0	0	0	3
slc43a2b	0	0	0	1	0	1	0	1	0	0	0	0	0	0	3
rpl19	0	0	0	1	0	1	0	0	0	0	0	1	0	0	3
c20h8orf82	0	0	0	1	0	1	1	0	0	0	0	0	0	0	3
c19h9orf91	0	0	0	0	1	1	0	1	0	0	0	0	0	0	3
gadd45bb	0	0	0	0	0	1	0	1	0	1	0	0	0	0	3
syt15	0	0	0	0	0	1	1	0	1	0	0	0	0	0	3
eef2l2	0	0	0	0	0	1	1	0	0	0	0	1	0	0	3
cbr4	0	0	0	0	0	1	1	0	0	0	0	1	0	0	3
aste1	0	0	0	0	0	1	0	0	1	1	0	0	0	0	3
spryd7a	0	0	0	0	0	1	1	0	0	1	0	0	0	0	3
dgcr2	0	0	0	0	0	1	1	0	0	0	0	0	1	0	3
adap2	0	0	0	0	0	1	1	0	0	0	0	1	0	0	3
tmem208	0	1	1	0	0	0	0	0	0	0	0	0	0	1	3
ctsz	0	1	1	1	0	0	0	0	0	0	0	0	0	0	3
gdpd3a	0	1	1	0	0	0	0	1	0	0	0	0	0	0	3
gnptg	0	1	1	0	1	0	0	0	0	0	0	0	0	0	3
mif	0	1	1	1	0	0	0	0	0	0	0	0	0	0	3
her13	0	1	1	0	0	0	1	0	0	0	0	0	0	0	3
pcgf5b	0	1	1	0	1	0	0	0	0	0	0	0	0	0	3
tgfb1b	0	1	1	0	0	0	0	0	1	0	0	0	0	0	3
tpma	0	1	1	0	1	0	0	0	0	0	0	0	0	0	3
rhd	0	0	1	0	0	0	1	0	0	0	0	0	1	0	3
tmem220	0	0	1	0	0	0	1	0	1	0	0	0	0	0	3
lyz	0	0	1	0	1	0	1	0	0	0	0	0	0	0	3
hrsp12	0	0	1	1	0	0	0	0	0	1	0	0	0	0	3
il7r	0	0	1	0	1	0	0	0	0	1	0	0	0	0	3
si:ch211-198m17.1	0	0	1	0	0	0	1	0	0	0	0	0	1	0	3
sla1	0	0	1	1	0	0	0	0	0	1	0	0	0	0	3
socs3b	0	0	1	0	1	0	1	0	0	0	0	0	0	0	3
slc6a19b	0	0	1	0	0	0	0	0	1	0	0	0	1	0	3
cptp	0	0	1	1	0	0	1	0	0	0	0	0	0	0	3
sft2d1	0	0	1	0	0	0	1	0	0	0	0	0	1	0	3
tfpi2	0	0	1	0	0	0	1	0	0	0	0	0	1	0	3
kctd12.2	0	0	1	1	1	0	0	0	0	0	0	0	0	0	3
reep3a	0	0	1	0	0	0	0	1	0	1	0	0	0	0	3
mob4	0	0	1	1	0	0	0	0	0	0	0	1	0	0	3
lfng	0	0	1	0	0	0	1	0	1	0	0	0	0	0	3
timp2b	0	0	1	0	1	0	0	0	1	0	0	0	0	0	3
zgc:162944	0	1	0	0	0	0	1	0	0	0	0	1	0	0	3
rell2	0	1	0	0	0	0	1	0	1	0	0	0	0	0	3
slc5a8l	0	1	0	0	1	0	0	0	1	0	0	0	0	0	3
zgc:113208	0	1	0	0	1	0	0	0	0	1	0	0	0	0	3
bx548047.2	0	1	0	0	0	0	1	0	0	0	0	1	0	0	3
si:dkey-29p10.4	0	1	0	0	0	0	0	1	0	0	0	0	1	0	3
asah2	0	1	0	1	0	0	0	1	0	0	0	0	0	0	3
gosr2	0	1	0	1	0	0	0	0	0	0	0	1	0	0	3
snx3	0	1	0	1	0	0	0	0	0	0	0	0	0	1	3
pex11g	0	1	0	1	0	0	1	0	0	0	0	0	0	0	3
cox7a3	0	1	0	0	1	0	0	0	0	0	1	0	0	0	3
parm1	0	1	0	1	0	0	0	0	0	0	0	0	1	0	3
arpc5b	0	1	0	1	1	0	0	0	0	0	0	0	0	0	3

b2ml	0	1	0	1	0	0	0	0	0	1	0	0	0	0	3
zgc:153031	0	0	0	1	1	0	1	0	0	0	0	0	0	0	3
hsd11b1la	0	0	0	1	0	0	1	1	0	0	0	0	0	0	3
slc7a10a	0	0	0	1	0	0	0	0	0	1	0	0	0	1	3
zgc:77938	0	0	0	0	1	0	1	0	0	0	0	0	1	0	3
bnip3lb	0	0	0	0	1	0	0	1	0	1	0	0	0	0	3
hopx	0	0	0	0	0	0	1	0	0	0	1	1	0	0	3
fbp1a	0	0	0	0	0	0	1	1	0	1	0	0	0	0	3
3-Sep	0	0	0	0	0	0	1	0	0	1	0	0	0	1	3
g6pca.1	0	0	0	0	0	0	1	0	1	1	0	0	0	0	3
tmem196b	0	0	0	0	0	0	1	0	0	0	0	1	1	0	3
cldnh	0	0	0	0	0	0	1	0	0	0	1	1	0	0	3
arhgap15	0	0	0	0	0	0	1	0	0	0	0	1	0	1	3
slc6a19a.2	0	0	0	0	0	0	1	0	1	1	0	0	0	0	3
vamp3	0	0	0	0	0	0	1	1	0	1	0	0	0	0	3
higd2a	0	0	0	0	0	0	0	0	0	1	1	1	0	0	3
ndufa3	0	0	1	0	0	1	0	0	0	0	0	0	0	0	2
pim2	0	0	1	0	0	1	0	0	0	0	0	0	0	0	2
si:ch211-283g2.2	0	0	1	0	0	1	0	0	0	0	0	0	0	0	2
dnase1l3	0	0	1	0	0	1	0	0	0	0	0	0	0	0	2
zgc:112160	0	0	1	0	0	1	0	0	0	0	0	0	0	0	2
slc15a1b	0	0	1	0	0	1	0	0	0	0	0	0	0	0	2
zgc:171759	0	0	1	0	0	1	0	0	0	0	0	0	0	0	2
rtn4ip1	0	0	1	0	0	1	0	0	0	0	0	0	0	0	2
pnoca	0	0	1	0	0	1	0	0	0	0	0	0	0	0	2
gpcpd1	0	0	1	0	0	1	0	0	0	0	0	0	0	0	2
vps33b	0	0	1	0	0	1	0	0	0	0	0	0	0	0	2
pla2g12b	0	0	1	0	0	1	0	0	0	0	0	0	0	0	2
arhgdig	0	0	1	0	0	1	0	0	0	0	0	0	0	0	2
tceb2	0	0	1	0	0	1	0	0	0	0	0	0	0	0	2
upp2	0	1	0	0	0	1	0	0	0	0	0	0	0	0	2
gng12a	0	1	0	0	0	1	0	0	0	0	0	0	0	0	2
klf11b	0	1	0	0	0	1	0	0	0	0	0	0	0	0	2
yrk	0	1	0	0	0	1	0	0	0	0	0	0	0	0	2
mospd1	0	1	0	0	0	1	0	0	0	0	0	0	0	0	2
si:ch211-153b23.7	0	1	0	0	0	1	0	0	0	0	0	0	0	0	2
cahz	0	0	0	1	0	1	0	0	0	0	0	0	0	0	2
si:dkey-51e6.1	0	0	0	1	0	1	0	0	0	0	0	0	0	0	2
stx2a	0	0	0	0	1	1	0	0	0	0	0	0	0	0	2
calm2b	0	0	0	0	1	1	0	0	0	0	0	0	0	0	2
zgc:136872	0	0	0	0	1	1	0	0	0	0	0	0	0	0	2
slc16a3	0	0	0	0	1	1	0	0	0	0	0	0	0	0	2
manf	0	0	0	0	1	1	0	0	0	0	0	0	0	0	2
npsn	0	0	0	0	0	1	0	0	0	1	0	0	0	0	2
rps20	0	0	0	0	0	1	0	0	0	1	0	0	0	0	2
plaua	0	0	0	0	0	1	1	0	0	0	0	0	0	0	2
zgc:77816	0	0	0	0	0	1	1	0	0	0	0	0	0	0	2
mgst1.2	0	0	0	0	0	1	0	0	0	0	0	0	1	0	2
cyth4a	0	0	0	0	0	1	0	0	1	0	0	0	0	0	2
s100v1	0	0	0	0	0	1	0	0	0	0	0	0	1	0	2
im:7151449	0	0	0	0	0	1	0	1	0	0	0	0	0	0	2
si:dkey-111e8.5	0	0	0	0	0	1	0	0	0	0	0	1	0	0	2
tspan37	0	0	0	0	0	1	0	0	0	1	0	0	0	0	2
zgc:92326	0	0	0	0	0	1	1	0	0	0	0	0	0	0	2
slc13a2	0	0	0	0	0	1	1	0	0	0	0	0	0	0	2
cbx7a	0	0	0	0	0	1	0	1	0	0	0	0	0	0	2
lad1	0	0	0	0	0	1	1	0	0	0	0	0	0	0	2
cyp2p6	0	0	0	0	0	1	1	0	0	0	0	0	0	0	2
fis1	0	0	0	0	0	1	0	0	1	0	0	0	0	0	2
zgc:136791	0	0	0	0	0	1	0	0	0	1	0	0	0	0	2
baiap2l2a	0	0	0	0	0	1	0	0	0	1	0	0	0	0	2
lgals9l1	0	0	0	0	0	1	1	0	0	0	0	0	0	0	2
cox8a	0	0	0	0	0	1	0	0	0	1	0	0	0	0	2
gadd45ba	0	0	0	0	0	1	0	0	0	0	0	1	0	0	2
id2b	0	0	0	0	0	1	1	0	0	0	0	0	0	0	2
phlda2	0	0	0	0	0	1	0	0	0	0	0	0	1	0	2
rpl23a	0	0	0	0	0	1	0	0	0	0	1	0	0	0	2
zgc:174888	0	0	0	0	0	1	0	0	0	0	1	0	0	0	2
sdccag3	0	0	0	0	0	1	0	1	0	0	0	0	0	0	2
rbms2b	0	0	0	0	0	1	0	0	0	1	0	0	0	0	2
tcta	0	0	0	0	0	1	1	0	0	0	0	0	0	0	2
arpc5a	0	0	0	0	0	1	0	0	0	1	0	0	0	0	2
si:ch211-196h16.5	0	0	0	0	0	1	0	0	0	1	0	0	0	0	2
tnnc2	0	0	0	0	0	1	0	1	0	0	0	0	0	0	2
cisd1	0	0	0	0	0	1	0	0	0	0	0	1	0	0	2
si:ch211-240l19.5	0	1	1	0	0	0	0	0	0	0	0	0	0	0	2
zgc:158432	0	1	1	0	0	0	0	0	0	0	0	0	0	0	2
pimr171	0	1	1	0	0	0	0	0	0	0	0	0	0	0	2
zgc:110339	0	1	1	0	0	0	0	0	0	0	0	0	0	0	2
igsf6	0	1	1	0	0	0	0	0	0	0	0	0	0	0	2
lmo2	0	1	1	0	0	0	0	0	0	0	0	0	0	0	2
sp7	0	1	1	0	0	0	0	0	0	0	0	0	0	0	2
si:dkeyp-73d8.8	0	1	1	0	0	0	0	0	0	0	0	0	0	0	2
endod1	0	1	1	0	0	0	0	0	0	0	0	0	0	0	2
si:ch211-170d8.5	0	1	1	0	0	0	0	0	0	0	0	0	0	0	2
pllp	0	1	1	0	0	0	0	0	0	0	0	0	0	0	2
si:ch73-380l3.1	0	1	1	0	0	0	0	0	0	0	0	0	0	0	2
mgst2	0	1	1	0	0	0	0	0	0	0	0	0	0	0	2
zgc:123297	0	1	1	0	0	0	0	0	0	0	0	0	0	0	2

phkg1b	0	1	1	0	0	0	0	0	0	0	0	0	0	0	2
ada	0	0	1	0	0	0	1	0	0	0	0	0	0	0	2
si:ch211-114l13.9	0	0	1	0	0	0	1	0	0	0	0	0	0	0	2
acta2	0	0	1	0	0	0	1	0	0	0	0	0	0	0	2
si:dkey-248g21.1	0	0	1	0	1	0	0	0	0	0	0	0	0	0	2
gabarapb	0	0	1	0	0	0	0	0	0	1	0	0	0	0	2
si:dkey-58f10.11	0	0	1	1	0	0	0	0	0	0	0	0	0	0	2
aqp9b	0	0	1	0	0	0	0	0	0	0	0	0	1	0	2
akr1b1	0	0	1	0	0	0	0	1	0	0	0	0	0	0	2
cbln14	0	0	1	0	0	0	0	1	0	0	0	0	0	0	2
si:ch73-343l4.8	0	0	1	0	0	0	1	0	0	0	0	0	0	0	2
glrx	0	0	1	0	0	0	0	0	0	0	0	0	0	1	2
mep1a.1	0	0	1	0	0	0	0	0	0	1	0	0	0	0	2
si:ch73-14h1.2	0	0	1	0	0	0	0	1	0	0	0	0	0	0	2
anxa13	0	0	1	0	0	0	0	0	0	0	0	0	1	0	2
ccng1	0	0	1	0	0	0	0	0	0	1	0	0	0	0	2
angptl4	0	0	1	0	0	0	0	0	0	0	0	0	1	0	2
rpl37	0	0	1	0	0	0	0	0	0	0	1	0	0	0	2
ndufs8a	0	0	1	1	0	0	0	0	0	0	0	0	0	0	2
bnip4	0	0	1	0	0	0	0	0	0	0	0	0	1	0	2
slc6a19a.1	0	0	1	0	0	0	0	0	0	1	0	0	0	0	2
grhprb	0	0	1	0	0	0	1	0	0	0	0	0	0	0	2
s1pr5a	0	0	1	0	0	0	0	0	0	0	0	1	0	0	2
tnfaip8l3	0	0	1	0	0	0	1	0	0	0	0	0	0	0	2
mep1a.2	0	0	1	0	0	0	0	0	0	0	0	1	0	0	2
obsl1b	0	0	1	1	0	0	0	0	0	0	0	0	0	0	2
cmah	0	0	1	0	0	0	0	0	1	0	0	0	0	0	2
ndufv2	0	0	1	0	0	0	0	0	0	0	0	0	1	0	2
isca2	0	0	1	0	0	0	1	0	0	0	0	0	0	0	2
c22h19orf60	0	0	1	0	0	0	1	0	0	0	0	0	0	0	2
zgc:101846	0	0	1	0	0	0	0	1	0	0	0	0	0	0	2
nceh1a	0	0	1	0	0	0	0	0	0	1	0	0	0	0	2
epor	0	0	1	0	0	0	1	0	0	0	0	0	0	0	2
adra1bb	0	0	1	0	0	0	0	0	0	0	0	0	0	1	2
txndc9	0	0	1	0	0	0	1	0	0	0	0	0	0	0	2
map1lc3a	0	0	1	0	0	0	1	0	0	0	0	0	0	0	2
tmem229b	0	0	1	0	0	0	0	0	0	1	0	0	0	0	2
sumf2	0	0	1	0	0	0	0	0	0	1	0	0	0	0	2
myct1a	0	0	1	0	0	0	0	0	0	0	0	0	1	0	2
sdhaf2	0	0	1	0	0	0	0	0	0	0	1	0	0	0	2
fpr1	0	0	1	0	0	0	0	0	0	0	0	1	0	0	2
gltpd2	0	0	1	0	0	0	0	0	0	0	0	1	0	0	2
elf3	0	0	1	0	0	0	1	0	0	0	0	0	0	0	2
si:dkey-25e12.3	0	0	1	0	1	0	0	0	0	0	0	0	0	0	2
alkbh6	0	0	1	0	0	0	0	1	0	0	0	0	0	0	2
rap1b	0	0	1	0	0	0	0	0	0	0	0	1	0	0	2
slc3a2a	0	0	1	0	0	0	0	0	0	1	0	0	0	0	2
dbi	0	1	0	0	0	0	0	0	0	0	0	0	0	1	2
krt4	0	1	0	0	0	0	0	0	0	0	0	0	0	1	2
btf3	0	1	0	1	0	0	0	0	0	0	0	0	0	0	2
tmem176l.4	0	1	0	0	0	0	0	0	0	1	0	0	0	0	2
ier3ip1	0	1	0	0	0	0	0	0	1	0	0	0	0	0	2
gng2	0	1	0	0	0	0	0	0	0	1	0	0	0	0	2
arhgef3l	0	1	0	0	1	0	0	0	0	0	0	0	0	0	2
cd74b	0	1	0	0	0	0	0	0	0	0	0	1	0	0	2
c5h3orf30	0	1	0	0	0	0	1	0	0	0	0	0	0	0	2
idh1	0	1	0	0	0	0	0	1	0	0	0	0	0	0	2
cmtm7	0	1	0	0	0	0	1	0	0	0	0	0	0	0	2
edf1	0	1	0	0	0	0	0	0	0	0	0	0	0	1	2
ctdsp2	0	1	0	0	0	0	0	0	0	0	0	0	1	0	2
sri	0	1	0	0	0	0	1	0	0	0	0	0	0	0	2
gpr182	0	1	0	0	0	0	1	0	0	0	0	0	0	0	2
atp6v1e1b	0	1	0	0	0	0	1	0	0	0	0	0	0	0	2
prdx6	0	1	0	0	0	0	0	0	0	0	0	1	0	0	2
rab40b	0	1	0	0	0	0	0	1	0	0	0	0	0	0	2
meig1	0	1	0	0	0	0	0	0	0	0	0	1	0	0	2
c2h14orf119	0	1	0	0	0	0	0	0	1	0	0	0	0	0	2
agtr2	0	1	0	0	1	0	0	0	0	0	0	0	0	0	2
psmg1	0	1	0	0	1	0	0	0	0	0	0	0	0	0	2
srp19	0	1	0	0	0	0	0	0	0	0	0	0	1	0	2
ufsp2	0	1	0	0	0	0	0	1	0	0	0	0	0	0	2
gmpr2	0	1	0	0	0	0	0	1	0	0	0	0	0	0	2
gig2e	0	1	0	0	0	0	0	0	1	0	0	0	0	0	2
ptk6b	0	1	0	0	0	0	0	0	1	0	0	0	0	0	2
zgc:92249	0	0	0	1	0	0	0	1	0	0	0	0	0	0	2
ace2	0	0	0	1	0	0	1	0	0	0	0	0	0	0	2
skp1	0	0	0	1	0	0	1	0	0	0	0	0	0	0	2
gpia	0	0	0	1	0	0	0	1	0	0	0	0	0	0	2
trappc3	0	0	0	1	0	0	0	1	0	0	0	0	0	0	2
sod1	0	0	0	0	1	0	0	0	1	0	0	0	0	0	2
ftr76	0	0	0	0	1	0	1	0	0	0	0	0	0	0	2
calua	0	0	0	0	1	0	0	0	0	0	0	1	0	0	2
ifit16	0	0	0	0	1	0	1	0	0	0	0	0	0	0	2
si:dkey-78a14.5	0	0	0	0	1	0	0	0	0	0	0	0	1	0	2
b9d1	0	0	0	0	1	0	0	1	0	0	0	0	0	0	2
si:ch211-140l13.3	0	0	0	0	1	0	0	0	0	0	1	0	0	0	2
gna12a	0	0	0	0	1	0	0	0	0	1	0	0	0	0	2
mcamb	0	0	0	0	1	0	0	0	0	1	0	0	0	0	2
il11a	0	0	0	0	0	0	1	0	0	1	0	0	0	0	2

lyl1	0	0	0	0	0	0	1	0	0	0	0	1	0	0	2
c5h8orf4	0	0	0	0	0	0	1	0	1	0	0	0	0	0	2
mdh1aa	0	0	0	0	0	0	1	0	0	1	0	0	0	0	2
ndufb10	0	0	0	0	0	0	1	0	0	0	0	0	1	0	2
nipsnap3a	0	0	0	0	0	0	1	0	0	1	0	0	0	0	2
stoml3b	0	0	0	0	0	0	1	0	0	0	0	0	1	0	2
wdr45b	0	0	0	0	0	0	1	0	0	1	0	0	0	0	2
bcap31	0	0	0	0	0	0	1	1	0	0	0	0	0	0	2
tmem45b	0	0	0	0	0	0	1	0	0	1	0	0	0	0	2
si:ch73-334d15.1	0	0	0	0	0	0	1	0	0	0	0	0	1	0	2
ctso	0	0	0	0	0	0	1	0	0	0	0	0	1	0	2
mylz3	0	0	0	0	0	0	1	0	0	0	0	0	0	1	2
krt8	0	0	0	0	0	0	1	1	0	0	0	0	0	0	2
ifit8	0	0	0	0	0	0	1	0	0	0	0	0	1	0	2
slc35d1b	0	0	0	0	0	0	1	1	0	0	0	0	0	0	2
sepp1a	0	0	0	0	0	0	1	1	0	0	0	0	0	0	2
rab7b	0	0	0	0	0	0	1	0	0	0	0	1	0	0	2
hsc70	0	0	0	0	0	0	0	1	0	0	0	1	0	0	2
slc16a9a	0	0	0	0	0	0	0	1	0	0	0	0	1	0	2
cpb1	0	0	0	0	0	0	0	1	0	0	0	1	0	0	2
dpm1	0	0	0	0	0	0	0	1	0	0	0	1	0	0	2
zgc:174259	0	0	0	0	0	0	0	1	0	0	0	1	0	0	2
ift20	0	0	0	0	0	0	0	1	0	0	0	0	1	0	2
sepw2a	0	0	0	0	0	0	0	1	0	0	0	0	1	0	2
tppp3	0	0	0	0	0	0	0	1	1	0	0	0	0	0	2
thbs1b	0	0	0	0	0	0	0	1	1	0	0	0	0	0	2
sar1ab	0	0	0	0	0	0	0	1	1	0	0	0	0	0	2
ckmb	0	0	0	0	0	0	0	0	1	1	0	0	0	0	2
zgc:172079	0	0	0	0	0	0	0	0	0	1	0	0	0	1	2
gca	0	0	0	0	0	0	0	0	0	1	1	0	0	0	2
zgc:172106	0	0	0	0	0	0	0	0	0	1	0	1	0	0	2
zgc:175284	0	0	0	0	0	0	0	0	0	1	0	0	0	1	2
taf13	0	0	0	0	0	0	0	0	0	0	0	1	1	0	2
dennd1c	0	0	0	0	0	0	0	0	0	0	0	0	1	1	2
plac8l1	0	0	0	0	0	1	0	0	0	0	0	0	0	0	1
lgals2b	0	0	0	0	0	1	0	0	0	0	0	0	0	0	1
fabp10a	0	0	0	0	0	1	0	0	0	0	0	0	0	0	1
si:dkey-286j15.3	0	0	0	0	0	1	0	0	0	0	0	0	0	0	1
gip	0	0	0	0	0	1	0	0	0	0	0	0	0	0	1
parvg	0	0	0	0	0	1	0	0	0	0	0	0	0	0	1
tdo2b	0	0	0	0	0	1	0	0	0	0	0	0	0	0	1
sycn.1	0	0	0	0	0	1	0	0	0	0	0	0	0	0	1
echdc2	0	0	0	0	0	1	0	0	0	0	0	0	0	0	1
chia.3	0	0	0	0	0	1	0	0	0	0	0	0	0	0	1
lrrc15	0	0	0	0	0	1	0	0	0	0	0	0	0	0	1
c6ast3	0	0	0	0	0	1	0	0	0	0	0	0	0	0	1
tm4sf5	0	0	0	0	0	1	0	0	0	0	0	0	0	0	1
pvalb1	0	0	0	0	0	1	0	0	0	0	0	0	0	0	1
si:ch211-217g15.3	0	0	0	0	0	1	0	0	0	0	0	0	0	0	1
ifit14	0	0	0	0	0	1	0	0	0	0	0	0	0	0	1
si:dkey-228a15.1	0	0	0	0	0	1	0	0	0	0	0	0	0	0	1
ak3	0	0	0	0	0	1	0	0	0	0	0	0	0	0	1
ak2	0	0	0	0	0	1	0	0	0	0	0	0	0	0	1
tmem167a	0	0	0	0	0	1	0	0	0	0	0	0	0	0	1
pla2g1b	0	0	0	0	0	1	0	0	0	0	0	0	0	0	1
anxa3b	0	0	0	0	0	1	0	0	0	0	0	0	0	0	1
pdia2	0	0	0	0	0	1	0	0	0	0	0	0	0	0	1
etv2	0	0	0	0	0	1	0	0	0	0	0	0	0	0	1
ggcta	0	0	0	0	0	1	0	0	0	0	0	0	0	0	1
rpl7	0	0	0	0	0	1	0	0	0	0	0	0	0	0	1
si:rp71-36a1.2	0	0	0	0	0	1	0	0	0	0	0	0	0	0	1
tmem72	0	0	0	0	0	1	0	0	0	0	0	0	0	0	1
rpl34	0	0	0	0	0	1	0	0	0	0	0	0	0	0	1
c22h19orf70	0	0	0	0	0	1	0	0	0	0	0	0	0	0	1
pecam1	0	0	0	0	0	1	0	0	0	0	0	0	0	0	1
psmb4	0	0	0	0	0	1	0	0	0	0	0	0	0	0	1
gp1bb	0	0	0	0	0	1	0	0	0	0	0	0	0	0	1
smim7	0	0	0	0	0	1	0	0	0	0	0	0	0	0	1
hgd	0	0	0	0	0	1	0	0	0	0	0	0	0	0	1
cln3	0	0	0	0	0	1	0	0	0	0	0	0	0	0	1
churc1	0	0	0	0	0	1	0	0	0	0	0	0	0	0	1
sh3bgrl2	0	0	0	0	0	1	0	0	0	0	0	0	0	0	1
ociad2	0	0	0	0	0	1	0	0	0	0	0	0	0	0	1
pyyb	0	0	0	0	0	1	0	0	0	0	0	0	0	0	1
olfml3b	0	0	0	0	0	1	0	0	0	0	0	0	0	0	1
ak4	0	0	0	0	0	1	0	0	0	0	0	0	0	0	1
ela3l	0	0	0	0	0	1	0	0	0	0	0	0	0	0	1
il15	0	0	0	0	0	1	0	0	0	0	0	0	0	0	1
iscu	0	0	0	0	0	1	0	0	0	0	0	0	0	0	1
ckba	0	0	0	0	0	1	0	0	0	0	0	0	0	0	1
cyp51	0	0	0	0	0	1	0	0	0	0	0	0	0	0	1
psma8	0	0	0	0	0	1	0	0	0	0	0	0	0	0	1
pin4	0	0	0	0	0	1	0	0	0	0	0	0	0	0	1
dhdds	0	0	0	0	0	1	0	0	0	0	0	0	0	0	1
zgc:112148	0	0	0	0	0	1	0	0	0	0	0	0	0	0	1
dhrs2	0	0	0	0	0	1	0	0	0	0	0	0	0	0	1
cyp3c3	0	0	0	0	0	1	0	0	0	0	0	0	0	0	1
wasa	0	0	0	0	0	1	0	0	0	0	0	0	0	0	1
zan	0	0	0	0	0	1	0	0	0	0	0	0	0	0	1

serp1	0	0	0	0	0	1	0	0	0	0	0	0	0	0	1
il17rel	0	0	0	0	0	1	0	0	0	0	0	0	0	0	1
klf1	0	0	0	0	0	1	0	0	0	0	0	0	0	0	1
si:ch211-226m16.2	0	0	0	0	0	1	0	0	0	0	0	0	0	0	1
hoxb6a	0	0	0	0	0	1	0	0	0	0	0	0	0	0	1
mest	0	0	0	0	0	1	0	0	0	0	0	0	0	0	1
si:ch211-133l5.7	0	0	0	0	0	1	0	0	0	0	0	0	0	0	1
znf521	0	0	0	0	0	1	0	0	0	0	0	0	0	0	1
skap2	0	0	0	0	0	1	0	0	0	0	0	0	0	0	1
zgc:153642	0	0	0	0	0	1	0	0	0	0	0	0	0	0	1
nit1	0	0	0	0	0	1	0	0	0	0	0	0	0	0	1
anxa2a	0	0	1	0	0	0	0	0	0	0	0	0	0	0	1
srgn	0	0	1	0	0	0	0	0	0	0	0	0	0	0	1
si:ch211-201h21.5	0	0	1	0	0	0	0	0	0	0	0	0	0	0	1
tmem176	0	0	1	0	0	0	0	0	0	0	0	0	0	0	1
cox7a1	0	0	1	0	0	0	0	0	0	0	0	0	0	0	1
si:ch211-66e2.3	0	0	1	0	0	0	0	0	0	0	0	0	0	0	1
si:ch1073-78m18.1	0	0	1	0	0	0	0	0	0	0	0	0	0	0	1
cenpv	0	0	1	0	0	0	0	0	0	0	0	0	0	0	1
il21r.1	0	0	1	0	0	0	0	0	0	0	0	0	0	0	1
sult1st5	0	0	1	0	0	0	0	0	0	0	0	0	0	0	1
rps27.1	0	0	1	0	0	0	0	0	0	0	0	0	0	0	1
zgc:158387	0	0	1	0	0	0	0	0	0	0	0	0	0	0	1
fkbp11	0	0	1	0	0	0	0	0	0	0	0	0	0	0	1
cd53	0	0	1	0	0	0	0	0	0	0	0	0	0	0	1
rnaseka	0	0	1	0	0	0	0	0	0	0	0	0	0	0	1
phlda3	0	0	1	0	0	0	0	0	0	0	0	0	0	0	1
afp4	0	0	1	0	0	0	0	0	0	0	0	0	0	0	1
slc43a3a	0	0	1	0	0	0	0	0	0	0	0	0	0	0	1
itln2	0	0	1	0	0	0	0	0	0	0	0	0	0	0	1
cmtm3	0	0	1	0	0	0	0	0	0	0	0	0	0	0	1
rfesd	0	0	1	0	0	0	0	0	0	0	0	0	0	0	1
commd7	0	0	1	0	0	0	0	0	0	0	0	0	0	0	1
pcolcea	0	0	1	0	0	0	0	0	0	0	0	0	0	0	1
rnf183	0	0	1	0	0	0	0	0	0	0	0	0	0	0	1
si:ch211-194m7.3	0	0	1	0	0	0	0	0	0	0	0	0	0	0	1
ttr	0	0	1	0	0	0	0	0	0	0	0	0	0	0	1
rnaset2l	0	0	1	0	0	0	0	0	0	0	0	0	0	0	1
c13h2orf50	0	0	1	0	0	0	0	0	0	0	0	0	0	0	1
dusp23b	0	0	1	0	0	0	0	0	0	0	0	0	0	0	1
si:dkey-15j16.6	0	0	1	0	0	0	0	0	0	0	0	0	0	0	1
si:ch211-105c13.3	0	0	1	0	0	0	0	0	0	0	0	0	0	0	1
trappc1	0	0	1	0	0	0	0	0	0	0	0	0	0	0	1
hcar1-4	0	0	1	0	0	0	0	0	0	0	0	0	0	0	1
nfe2	0	0	1	0	0	0	0	0	0	0	0	0	0	0	1
rer1	0	0	1	0	0	0	0	0	0	0	0	0	0	0	1
cbln6	0	0	1	0	0	0	0	0	0	0	0	0	0	0	1
pstpip2	0	0	1	0	0	0	0	0	0	0	0	0	0	0	1
zmp:0000001085	0	0	1	0	0	0	0	0	0	0	0	0	0	0	1
calm1b	0	0	1	0	0	0	0	0	0	0	0	0	0	0	1
bud31	0	0	1	0	0	0	0	0	0	0	0	0	0	0	1
rpl18	0	0	1	0	0	0	0	0	0	0	0	0	0	0	1
atp6v1g1	0	0	1	0	0	0	0	0	0	0	0	0	0	0	1
parvab	0	0	1	0	0	0	0	0	0	0	0	0	0	0	1
brk1	0	0	1	0	0	0	0	0	0	0	0	0	0	0	1
zgc:173962	0	0	1	0	0	0	0	0	0	0	0	0	0	0	1
fth1b	0	0	1	0	0	0	0	0	0	0	0	0	0	0	1
rgs4	0	0	1	0	0	0	0	0	0	0	0	0	0	0	1
dnajc22	0	0	1	0	0	0	0	0	0	0	0	0	0	0	1
lipf	0	0	1	0	0	0	0	0	0	0	0	0	0	0	1
serpina10a	0	0	1	0	0	0	0	0	0	0	0	0	0	0	1
cfap126	0	0	1	0	0	0	0	0	0	0	0	0	0	0	1
rnf114	0	0	1	0	0	0	0	0	0	0	0	0	0	0	1
lrrc71	0	0	1	0	0	0	0	0	0	0	0	0	0	0	1
ackr4a	0	0	1	0	0	0	0	0	0	0	0	0	0	0	1
cotl1	0	0	1	0	0	0	0	0	0	0	0	0	0	0	1
cda	0	0	1	0	0	0	0	0	0	0	0	0	0	0	1
neo1b	1	0	0	0	0	0	0	0	0	0	0	0	0	0	1
zgc:56231	1	0	0	0	0	0	0	0	0	0	0	0	0	0	1
ddx41	1	0	0	0	0	0	0	0	0	0	0	0	0	0	1
lmo7b	1	0	0	0	0	0	0	0	0	0	0	0	0	0	1
osbpl7	1	0	0	0	0	0	0	0	0	0	0	0	0	0	1
zgc:66024	1	0	0	0	0	0	0	0	0	0	0	0	0	0	1
tmprss4b	1	0	0	0	0	0	0	0	0	0	0	0	0	0	1
zpcx	1	0	0	0	0	0	0	0	0	0	0	0	0	0	1
rif1	1	0	0	0	0	0	0	0	0	0	0	0	0	0	1
bora	1	0	0	0	0	0	0	0	0	0	0	0	0	0	1
si:ch211-250e5.16	1	0	0	0	0	0	0	0	0	0	0	0	0	0	1
zgc:55413	1	0	0	0	0	0	0	0	0	0	0	0	0	0	1
uchl1	1	0	0	0	0	0	0	0	0	0	0	0	0	0	1
cu326366.2	1	0	0	0	0	0	0	0	0	0	0	0	0	0	1
smc4	1	0	0	0	0	0	0	0	0	0	0	0	0	0	1
pola1	1	0	0	0	0	0	0	0	0	0	0	0	0	0	1
fam20cl	1	0	0	0	0	0	0	0	0	0	0	0	0	0	1
trpc5b	1	0	0	0	0	0	0	0	0	0	0	0	0	0	1
zgc:92287	1	0	0	0	0	0	0	0	0	0	0	0	0	0	1
zgc:113162	1	0	0	0	0	0	0	0	0	0	0	0	0	0	1
depdc1a	1	0	0	0	0	0	0	0	0	0	0	0	0	0	1
oxct1a	1	0	0	0	0	0	0	0	0	0	0	0	0	0	1

syt13	1	0	0	0	0	0	0	0	0	0	0	0	0	0	1
cachd1	1	0	0	0	0	0	0	0	0	0	0	0	0	0	1
setdb1a	1	0	0	0	0	0	0	0	0	0	0	0	0	0	1
ephb2b	1	0	0	0	0	0	0	0	0	0	0	0	0	0	1
eme1	1	0	0	0	0	0	0	0	0	0	0	0	0	0	1
tcf19l	1	0	0	0	0	0	0	0	0	0	0	0	0	0	1
psip1	1	0	0	0	0	0	0	0	0	0	0	0	0	0	1
kif2c	1	0	0	0	0	0	0	0	0	0	0	0	0	0	1
syncripl	1	0	0	0	0	0	0	0	0	0	0	0	0	0	1
si:ch211-156j22.4	1	0	0	0	0	0	0	0	0	0	0	0	0	0	1
st6gal1	1	0	0	0	0	0	0	0	0	0	0	0	0	0	1
arhgef7a	1	0	0	0	0	0	0	0	0	0	0	0	0	0	1
adcy5	1	0	0	0	0	0	0	0	0	0	0	0	0	0	1
ankle2	1	0	0	0	0	0	0	0	0	0	0	0	0	0	1
elovl7a	1	0	0	0	0	0	0	0	0	0	0	0	0	0	1
rasal2	1	0	0	0	0	0	0	0	0	0	0	0	0	0	1
f2rl1.1	1	0	0	0	0	0	0	0	0	0	0	0	0	0	1
klhl35	1	0	0	0	0	0	0	0	0	0	0	0	0	0	1
atp10a	1	0	0	0	0	0	0	0	0	0	0	0	0	0	1
fbxo33	1	0	0	0	0	0	0	0	0	0	0	0	0	0	1
slc7a4	1	0	0	0	0	0	0	0	0	0	0	0	0	0	1
zmym2	1	0	0	0	0	0	0	0	0	0	0	0	0	0	1
zgc:66455	1	0	0	0	0	0	0	0	0	0	0	0	0	0	1
pom121	1	0	0	0	0	0	0	0	0	0	0	0	0	0	1
ndst2a	1	0	0	0	0	0	0	0	0	0	0	0	0	0	1
si:ch211-181d7.3	1	0	0	0	0	0	0	0	0	0	0	0	0	0	1
tanc2a	1	0	0	0	0	0	0	0	0	0	0	0	0	0	1
ek1	1	0	0	0	0	0	0	0	0	0	0	0	0	0	1
sall4	1	0	0	0	0	0	0	0	0	0	0	0	0	0	1
adgrl2a	1	0	0	0	0	0	0	0	0	0	0	0	0	0	1
necap1	1	0	0	0	0	0	0	0	0	0	0	0	0	0	1
itpr2	1	0	0	0	0	0	0	0	0	0	0	0	0	0	1
tgfbrap1	1	0	0	0	0	0	0	0	0	0	0	0	0	0	1
chaf1a	1	0	0	0	0	0	0	0	0	0	0	0	0	0	1
uacab	1	0	0	0	0	0	0	0	0	0	0	0	0	0	1
dhx32a	1	0	0	0	0	0	0	0	0	0	0	0	0	0	1
plxna3	1	0	0	0	0	0	0	0	0	0	0	0	0	0	1
lef1	1	0	0	0	0	0	0	0	0	0	0	0	0	0	1
sept4a	1	0	0	0	0	0	0	0	0	0	0	0	0	0	1
stard8	1	0	0	0	0	0	0	0	0	0	0	0	0	0	1
gapvd1	1	0	0	0	0	0	0	0	0	0	0	0	0	0	1
exd2	1	0	0	0	0	0	0	0	0	0	0	0	0	0	1
shisa2	1	0	0	0	0	0	0	0	0	0	0	0	0	0	1
rgs7a	1	0	0	0	0	0	0	0	0	0	0	0	0	0	1
nxph1	1	0	0	0	0	0	0	0	0	0	0	0	0	0	1
bin1b	1	0	0	0	0	0	0	0	0	0	0	0	0	0	1
gpr63	1	0	0	0	0	0	0	0	0	0	0	0	0	0	1
ror1	1	0	0	0	0	0	0	0	0	0	0	0	0	0	1
rundc1	1	0	0	0	0	0	0	0	0	0	0	0	0	0	1
ckbb	1	0	0	0	0	0	0	0	0	0	0	0	0	0	1
ska3	1	0	0	0	0	0	0	0	0	0	0	0	0	0	1
bard1	1	0	0	0	0	0	0	0	0	0	0	0	0	0	1
casd1	1	0	0	0	0	0	0	0	0	0	0	0	0	0	1
znf1007	1	0	0	0	0	0	0	0	0	0	0	0	0	0	1
ssh1b	1	0	0	0	0	0	0	0	0	0	0	0	0	0	1
ptgfrn	1	0	0	0	0	0	0	0	0	0	0	0	0	0	1
casp8ap2	1	0	0	0	0	0	0	0	0	0	0	0	0	0	1
cgnl1	1	0	0	0	0	0	0	0	0	0	0	0	0	0	1
zzz3	1	0	0	0	0	0	0	0	0	0	0	0	0	0	1
dok6	1	0	0	0	0	0	0	0	0	0	0	0	0	0	1
ccdc88c	1	0	0	0	0	0	0	0	0	0	0	0	0	0	1
slc12a8	1	0	0	0	0	0	0	0	0	0	0	0	0	0	1
rrm1	1	0	0	0	0	0	0	0	0	0	0	0	0	0	1
fxr2	1	0	0	0	0	0	0	0	0	0	0	0	0	0	1
cu459160.2	1	0	0	0	0	0	0	0	0	0	0	0	0	0	1
lrrc8aa	1	0	0	0	0	0	0	0	0	0	0	0	0	0	1
si:ch211-183d21.1	1	0	0	0	0	0	0	0	0	0	0	0	0	0	1
tenm2	1	0	0	0	0	0	0	0	0	0	0	0	0	0	1
myh9a	1	0	0	0	0	0	0	0	0	0	0	0	0	0	1
mtfr2	1	0	0	0	0	0	0	0	0	0	0	0	0	0	1
sin3b	1	0	0	0	0	0	0	0	0	0	0	0	0	0	1
znf507	1	0	0	0	0	0	0	0	0	0	0	0	0	0	1
mllt1a	1	0	0	0	0	0	0	0	0	0	0	0	0	0	1
limch1a	1	0	0	0	0	0	0	0	0	0	0	0	0	0	1
ddx31	1	0	0	0	0	0	0	0	0	0	0	0	0	0	1
tmtopsb	1	0	0	0	0	0	0	0	0	0	0	0	0	0	1
med13a	1	0	0	0	0	0	0	0	0	0	0	0	0	0	1
mllt3	1	0	0	0	0	0	0	0	0	0	0	0	0	0	1
ankrd52a	1	0	0	0	0	0	0	0	0	0	0	0	0	0	1
gna11b	1	0	0	0	0	0	0	0	0	0	0	0	0	0	1
abhd6b	1	0	0	0	0	0	0	0	0	0	0	0	0	0	1
akap1b	1	0	0	0	0	0	0	0	0	0	0	0	0	0	1
gtf2a1l	1	0	0	0	0	0	0	0	0	0	0	0	0	0	1
ankle1	1	0	0	0	0	0	0	0	0	0	0	0	0	0	1
slc4a7	1	0	0	0	0	0	0	0	0	0	0	0	0	0	1
fancc	1	0	0	0	0	0	0	0	0	0	0	0	0	0	1
per3	1	0	0	0	0	0	0	0	0	0	0	0	0	0	1
cdc16	1	0	0	0	0	0	0	0	0	0	0	0	0	0	1
ipo13	1	0	0	0	0	0	0	0	0	0	0	0	0	0	1

slc4a2b	1	0	0	0	0	0	0	0	0	0	0	0	0	0	1
dhx29	1	0	0	0	0	0	0	0	0	0	0	0	0	0	1
tsc22d1	1	0	0	0	0	0	0	0	0	0	0	0	0	0	1
cep85	1	0	0	0	0	0	0	0	0	0	0	0	0	0	1
prr12a	1	0	0	0	0	0	0	0	0	0	0	0	0	0	1
igf1rb	1	0	0	0	0	0	0	0	0	0	0	0	0	0	1
e2f5	1	0	0	0	0	0	0	0	0	0	0	0	0	0	1
zranb3	1	0	0	0	0	0	0	0	0	0	0	0	0	0	1
ptpn4b	1	0	0	0	0	0	0	0	0	0	0	0	0	0	1
chsy1	1	0	0	0	0	0	0	0	0	0	0	0	0	0	1
lppr4a	1	0	0	0	0	0	0	0	0	0	0	0	0	0	1
rpgrip1l	1	0	0	0	0	0	0	0	0	0	0	0	0	0	1
slc24a4a	1	0	0	0	0	0	0	0	0	0	0	0	0	0	1
eif4g1a	1	0	0	0	0	0	0	0	0	0	0	0	0	0	1
inadl	1	0	0	0	0	0	0	0	0	0	0	0	0	0	1
dennd2db	1	0	0	0	0	0	0	0	0	0	0	0	0	0	1
mink1	1	0	0	0	0	0	0	0	0	0	0	0	0	0	1
si:dkey-105h11.2	1	0	0	0	0	0	0	0	0	0	0	0	0	0	1
cep120	1	0	0	0	0	0	0	0	0	0	0	0	0	0	1
cnot3b	1	0	0	0	0	0	0	0	0	0	0	0	0	0	1
tmem79a	1	0	0	0	0	0	0	0	0	0	0	0	0	0	1
ralgapa1	1	0	0	0	0	0	0	0	0	0	0	0	0	0	1
gltscr1	1	0	0	0	0	0	0	0	0	0	0	0	0	0	1
arhgap33	1	0	0	0	0	0	0	0	0	0	0	0	0	0	1
pcnt	1	0	0	0	0	0	0	0	0	0	0	0	0	0	1
si:dkey-67c22.2	1	0	0	0	0	0	0	0	0	0	0	0	0	0	1
si:dkey-114c15.7	1	0	0	0	0	0	0	0	0	0	0	0	0	0	1
cap1	1	0	0	0	0	0	0	0	0	0	0	0	0	0	1
far1	1	0	0	0	0	0	0	0	0	0	0	0	0	0	1
glipr2l	1	0	0	0	0	0	0	0	0	0	0	0	0	0	1
ctbp1	1	0	0	0	0	0	0	0	0	0	0	0	0	0	1
agpat4	1	0	0	0	0	0	0	0	0	0	0	0	0	0	1
micall1a	1	0	0	0	0	0	0	0	0	0	0	0	0	0	1
adnpb	1	0	0	0	0	0	0	0	0	0	0	0	0	0	1
capn12	1	0	0	0	0	0	0	0	0	0	0	0	0	0	1
ppp2r5d	1	0	0	0	0	0	0	0	0	0	0	0	0	0	1
hspa4a	1	0	0	0	0	0	0	0	0	0	0	0	0	0	1
scn8aa	1	0	0	0	0	0	0	0	0	0	0	0	0	0	1
zmat1	1	0	0	0	0	0	0	0	0	0	0	0	0	0	1
igf1ra	1	0	0	0	0	0	0	0	0	0	0	0	0	0	1
kif1b	1	0	0	0	0	0	0	0	0	0	0	0	0	0	1
ccser2a	1	0	0	0	0	0	0	0	0	0	0	0	0	0	1
bokb	1	0	0	0	0	0	0	0	0	0	0	0	0	0	1
trim71	1	0	0	0	0	0	0	0	0	0	0	0	0	0	1
si:dkey-10f21.4	1	0	0	0	0	0	0	0	0	0	0	0	0	0	1
ttll7	1	0	0	0	0	0	0	0	0	0	0	0	0	0	1
znf335	1	0	0	0	0	0	0	0	0	0	0	0	0	0	1
smad2	1	0	0	0	0	0	0	0	0	0	0	0	0	0	1
taf5	1	0	0	0	0	0	0	0	0	0	0	0	0	0	1
trim62	1	0	0	0	0	0	0	0	0	0	0	0	0	0	1
gys1	1	0	0	0	0	0	0	0	0	0	0	0	0	0	1
fbxo10	1	0	0	0	0	0	0	0	0	0	0	0	0	0	1
pi4kaa	1	0	0	0	0	0	0	0	0	0	0	0	0	0	1
cdr2a	1	0	0	0	0	0	0	0	0	0	0	0	0	0	1
fgd6	1	0	0	0	0	0	0	0	0	0	0	0	0	0	1
polr1b	1	0	0	0	0	0	0	0	0	0	0	0	0	0	1
bicc1a	1	0	0	0	0	0	0	0	0	0	0	0	0	0	1
prmt5	1	0	0	0	0	0	0	0	0	0	0	0	0	0	1
cdc42ep2	1	0	0	0	0	0	0	0	0	0	0	0	0	0	1
si:dkeyp-121d4.3	1	0	0	0	0	0	0	0	0	0	0	0	0	0	1
zbtb21	1	0	0	0	0	0	0	0	0	0	0	0	0	0	1
gnao1a	1	0	0	0	0	0	0	0	0	0	0	0	0	0	1
yeats2	1	0	0	0	0	0	0	0	0	0	0	0	0	0	1
nova1	1	0	0	0	0	0	0	0	0	0	0	0	0	0	1
rbm33	1	0	0	0	0	0	0	0	0	0	0	0	0	0	1
rbbp8	1	0	0	0	0	0	0	0	0	0	0	0	0	0	1
znf395a	1	0	0	0	0	0	0	0	0	0	0	0	0	0	1
bms1	1	0	0	0	0	0	0	0	0	0	0	0	0	0	1
gkap1	1	0	0	0	0	0	0	0	0	0	0	0	0	0	1
mapk1	1	0	0	0	0	0	0	0	0	0	0	0	0	0	1
slc43a1a	1	0	0	0	0	0	0	0	0	0	0	0	0	0	1
pde12	1	0	0	0	0	0	0	0	0	0	0	0	0	0	1
mark4a	1	0	0	0	0	0	0	0	0	0	0	0	0	0	1
srgap1a	1	0	0	0	0	0	0	0	0	0	0	0	0	0	1
ccdc149b	1	0	0	0	0	0	0	0	0	0	0	0	0	0	1
scrib	1	0	0	0	0	0	0	0	0	0	0	0	0	0	1
srpk1b	1	0	0	0	0	0	0	0	0	0	0	0	0	0	1
snx27b	1	0	0	0	0	0	0	0	0	0	0	0	0	0	1
atp2c1	1	0	0	0	0	0	0	0	0	0	0	0	0	0	1
si:dkey-253d23.5	1	0	0	0	0	0	0	0	0	0	0	0	0	0	1
mier3b	1	0	0	0	0	0	0	0	0	0	0	0	0	0	1
ezh2	1	0	0	0	0	0	0	0	0	0	0	0	0	0	1
sh3yl1	1	0	0	0	0	0	0	0	0	0	0	0	0	0	1
tmem102	1	0	0	0	0	0	0	0	0	0	0	0	0	0	1
ephb6	1	0	0	0	0	0	0	0	0	0	0	0	0	0	1
mad1l1	1	0	0	0	0	0	0	0	0	0	0	0	0	0	1
rab11al	1	0	0	0	0	0	0	0	0	0	0	0	0	0	1
nrip1b	1	0	0	0	0	0	0	0	0	0	0	0	0	0	1
polr2e	1	0	0	0	0	0	0	0	0	0	0	0	0	0	1

timeless	1	0	0	0	0	0	0	0	0	0	0	0	0	0	1
znf532	1	0	0	0	0	0	0	0	0	0	0	0	0	0	1
dnajc5aa	1	0	0	0	0	0	0	0	0	0	0	0	0	0	1
clip2	1	0	0	0	0	0	0	0	0	0	0	0	0	0	1
si:dkeyp-4c7.3	1	0	0	0	0	0	0	0	0	0	0	0	0	0	1
oprl1	1	0	0	0	0	0	0	0	0	0	0	0	0	0	1
eftud1	1	0	0	0	0	0	0	0	0	0	0	0	0	0	1
rab33a	1	0	0	0	0	0	0	0	0	0	0	0	0	0	1
cluha	1	0	0	0	0	0	0	0	0	0	0	0	0	0	1
rarab	1	0	0	0	0	0	0	0	0	0	0	0	0	0	1
nfrkb	1	0	0	0	0	0	0	0	0	0	0	0	0	0	1
rbpms2a	1	0	0	0	0	0	0	0	0	0	0	0	0	0	1
mybl2b	1	0	0	0	0	0	0	0	0	0	0	0	0	0	1
pdik1l	1	0	0	0	0	0	0	0	0	0	0	0	0	0	1
si:ch211-181d7.1	1	0	0	0	0	0	0	0	0	0	0	0	0	0	1
st6gal2b	1	0	0	0	0	0	0	0	0	0	0	0	0	0	1
trip6	1	0	0	0	0	0	0	0	0	0	0	0	0	0	1
neurod6b	1	0	0	0	0	0	0	0	0	0	0	0	0	0	1
slc25a44a	1	0	0	0	0	0	0	0	0	0	0	0	0	0	1
usp40	1	0	0	0	0	0	0	0	0	0	0	0	0	0	1
nav3	1	0	0	0	0	0	0	0	0	0	0	0	0	0	1
si:dkey-103i16.6	1	0	0	0	0	0	0	0	0	0	0	0	0	0	1
acvr1bb	1	0	0	0	0	0	0	0	0	0	0	0	0	0	1
pum2	1	0	0	0	0	0	0	0	0	0	0	0	0	0	1
pigo	1	0	0	0	0	0	0	0	0	0	0	0	0	0	1
gtf2a1	1	0	0	0	0	0	0	0	0	0	0	0	0	0	1
agap1	1	0	0	0	0	0	0	0	0	0	0	0	0	0	1
chd4b	1	0	0	0	0	0	0	0	0	0	0	0	0	0	1
ical1	1	0	0	0	0	0	0	0	0	0	0	0	0	0	1
golga3	1	0	0	0	0	0	0	0	0	0	0	0	0	0	1
epc2	1	0	0	0	0	0	0	0	0	0	0	0	0	0	1
l3mbtl3	1	0	0	0	0	0	0	0	0	0	0	0	0	0	1
cnp	1	0	0	0	0	0	0	0	0	0	0	0	0	0	1
cnnm2a	1	0	0	0	0	0	0	0	0	0	0	0	0	0	1
tnpo2	1	0	0	0	0	0	0	0	0	0	0	0	0	0	1
dlgap2a	1	0	0	0	0	0	0	0	0	0	0	0	0	0	1
dus1l	1	0	0	0	0	0	0	0	0	0	0	0	0	0	1
si:dkey-191g9.5	1	0	0	0	0	0	0	0	0	0	0	0	0	0	1
tsc1b	1	0	0	0	0	0	0	0	0	0	0	0	0	0	1
usp31	1	0	0	0	0	0	0	0	0	0	0	0	0	0	1
mvb12ba	1	0	0	0	0	0	0	0	0	0	0	0	0	0	1
arntl2	1	0	0	0	0	0	0	0	0	0	0	0	0	0	1
si:ch73-22o12.1	1	0	0	0	0	0	0	0	0	0	0	0	0	0	1
cul5a	1	0	0	0	0	0	0	0	0	0	0	0	0	0	1
trhde	1	0	0	0	0	0	0	0	0	0	0	0	0	0	1
frem2a	1	0	0	0	0	0	0	0	0	0	0	0	0	0	1
rab8a	1	0	0	0	0	0	0	0	0	0	0	0	0	0	1
ccdc120	1	0	0	0	0	0	0	0	0	0	0	0	0	0	1
rapgef2	1	0	0	0	0	0	0	0	0	0	0	0	0	0	1
pcnxl3	1	0	0	0	0	0	0	0	0	0	0	0	0	0	1
c19h6orf136	1	0	0	0	0	0	0	0	0	0	0	0	0	0	1
zc3h12b	1	0	0	0	0	0	0	0	0	0	0	0	0	0	1
tom1l2	1	0	0	0	0	0	0	0	0	0	0	0	0	0	1
sh3rf1	1	0	0	0	0	0	0	0	0	0	0	0	0	0	1
dhx16	1	0	0	0	0	0	0	0	0	0	0	0	0	0	1
insrb	1	0	0	0	0	0	0	0	0	0	0	0	0	0	1
aco1	1	0	0	0	0	0	0	0	0	0	0	0	0	0	1
asb3	1	0	0	0	0	0	0	0	0	0	0	0	0	0	1
cep290	1	0	0	0	0	0	0	0	0	0	0	0	0	0	1
pdp2	1	0	0	0	0	0	0	0	0	0	0	0	0	0	1
tsc1a	1	0	0	0	0	0	0	0	0	0	0	0	0	0	1
ncoa1	1	0	0	0	0	0	0	0	0	0	0	0	0	0	1
upf1	1	0	0	0	0	0	0	0	0	0	0	0	0	0	1
cltca	1	0	0	0	0	0	0	0	0	0	0	0	0	0	1
vps37b	1	0	0	0	0	0	0	0	0	0	0	0	0	0	1
ptprdb	1	0	0	0	0	0	0	0	0	0	0	0	0	0	1
cyp17a2	1	0	0	0	0	0	0	0	0	0	0	0	0	0	1
epg5	1	0	0	0	0	0	0	0	0	0	0	0	0	0	1
nphp1	1	0	0	0	0	0	0	0	0	0	0	0	0	0	1
disc1	1	0	0	0	0	0	0	0	0	0	0	0	0	0	1
acer1	1	0	0	0	0	0	0	0	0	0	0	0	0	0	1
skila	1	0	0	0	0	0	0	0	0	0	0	0	0	0	1
zfyve28	1	0	0	0	0	0	0	0	0	0	0	0	0	0	1
efna5a	1	0	0	0	0	0	0	0	0	0	0	0	0	0	1
cenpu	1	0	0	0	0	0	0	0	0	0	0	0	0	0	1
arfgef1	1	0	0	0	0	0	0	0	0	0	0	0	0	0	1
kpna6	1	0	0	0	0	0	0	0	0	0	0	0	0	0	1
ambra1a	1	0	0	0	0	0	0	0	0	0	0	0	0	0	1
pknox2	1	0	0	0	0	0	0	0	0	0	0	0	0	0	1
sphk2	1	0	0	0	0	0	0	0	0	0	0	0	0	0	1
zfand5b	1	0	0	0	0	0	0	0	0	0	0	0	0	0	1
kirrel3a	1	0	0	0	0	0	0	0	0	0	0	0	0	0	1
znf839	1	0	0	0	0	0	0	0	0	0	0	0	0	0	1
syngr1a	1	0	0	0	0	0	0	0	0	0	0	0	0	0	1
tead1a	1	0	0	0	0	0	0	0	0	0	0	0	0	0	1
tnikb	1	0	0	0	0	0	0	0	0	0	0	0	0	0	1
sf3b2	1	0	0	0	0	0	0	0	0	0	0	0	0	0	1
galnt2	1	0	0	0	0	0	0	0	0	0	0	0	0	0	1
qrich1	1	0	0	0	0	0	0	0	0	0	0	0	0	0	1

atp8b3	1	0	0	0	0	0	0	0	0	0	0	0	0	0	1
brpf3b	1	0	0	0	0	0	0	0	0	0	0	0	0	0	1
prdm9	1	0	0	0	0	0	0	0	0	0	0	0	0	0	1
lamp2	1	0	0	0	0	0	0	0	0	0	0	0	0	0	1
mpped2a	1	0	0	0	0	0	0	0	0	0	0	0	0	0	1
ssx2ipa	1	0	0	0	0	0	0	0	0	0	0	0	0	0	1
si:ch211-218o21.4	1	0	0	0	0	0	0	0	0	0	0	0	0	0	1
tmpob	1	0	0	0	0	0	0	0	0	0	0	0	0	0	1
flot1a	1	0	0	0	0	0	0	0	0	0	0	0	0	0	1
mical1	1	0	0	0	0	0	0	0	0	0	0	0	0	0	1
kcnab2	1	0	0	0	0	0	0	0	0	0	0	0	0	0	1
usp24	1	0	0	0	0	0	0	0	0	0	0	0	0	0	1
snapc4	1	0	0	0	0	0	0	0	0	0	0	0	0	0	1
zbtb3	1	0	0	0	0	0	0	0	0	0	0	0	0	0	1
cntnap5l	1	0	0	0	0	0	0	0	0	0	0	0	0	0	1
samd10b	1	0	0	0	0	0	0	0	0	0	0	0	0	0	1
ccnt1	1	0	0	0	0	0	0	0	0	0	0	0	0	0	1
cu856539.3	1	0	0	0	0	0	0	0	0	0	0	0	0	0	1
map7d3	1	0	0	0	0	0	0	0	0	0	0	0	0	0	1
laptm4b	1	0	0	0	0	0	0	0	0	0	0	0	0	0	1
prmt7	1	0	0	0	0	0	0	0	0	0	0	0	0	0	1
otud7b	1	0	0	0	0	0	0	0	0	0	0	0	0	0	1
trim23	1	0	0	0	0	0	0	0	0	0	0	0	0	0	1
yars2	1	0	0	0	0	0	0	0	0	0	0	0	0	0	1
ptprm	1	0	0	0	0	0	0	0	0	0	0	0	0	0	1
mphosph10	1	0	0	0	0	0	0	0	0	0	0	0	0	0	1
rbm15	1	0	0	0	0	0	0	0	0	0	0	0	0	0	1
nedd4l	1	0	0	0	0	0	0	0	0	0	0	0	0	0	1
itgb5	1	0	0	0	0	0	0	0	0	0	0	0	0	0	1
jade1	1	0	0	0	0	0	0	0	0	0	0	0	0	0	1
fermt2	1	0	0	0	0	0	0	0	0	0	0	0	0	0	1
snrka	1	0	0	0	0	0	0	0	0	0	0	0	0	0	1
snx13	1	0	0	0	0	0	0	0	0	0	0	0	0	0	1
pfkfb2a	1	0	0	0	0	0	0	0	0	0	0	0	0	0	1
c21h17orf85	1	0	0	0	0	0	0	0	0	0	0	0	0	0	1
neurl4	1	0	0	0	0	0	0	0	0	0	0	0	0	0	1
sort1a	1	0	0	0	0	0	0	0	0	0	0	0	0	0	1
col9a2	1	0	0	0	0	0	0	0	0	0	0	0	0	0	1
zgc:92218	1	0	0	0	0	0	0	0	0	0	0	0	0	0	1
cdc23	1	0	0	0	0	0	0	0	0	0	0	0	0	0	1
amotl1	1	0	0	0	0	0	0	0	0	0	0	0	0	0	1
arfip2a	1	0	0	0	0	0	0	0	0	0	0	0	0	0	1
ppp4ca	1	0	0	0	0	0	0	0	0	0	0	0	0	0	1
st8sia2	1	0	0	0	0	0	0	0	0	0	0	0	0	0	1
fam222a	1	0	0	0	0	0	0	0	0	0	0	0	0	0	1
pomgnt1	1	0	0	0	0	0	0	0	0	0	0	0	0	0	1
glcci1	1	0	0	0	0	0	0	0	0	0	0	0	0	0	1
larp7	1	0	0	0	0	0	0	0	0	0	0	0	0	0	1
mapk9	1	0	0	0	0	0	0	0	0	0	0	0	0	0	1
ythdf1	1	0	0	0	0	0	0	0	0	0	0	0	0	0	1
senp1	1	0	0	0	0	0	0	0	0	0	0	0	0	0	1
atrn	1	0	0	0	0	0	0	0	0	0	0	0	0	0	1
esf1	1	0	0	0	0	0	0	0	0	0	0	0	0	0	1
ankrd10a	1	0	0	0	0	0	0	0	0	0	0	0	0	0	1
gak	1	0	0	0	0	0	0	0	0	0	0	0	0	0	1
znf646	1	0	0	0	0	0	0	0	0	0	0	0	0	0	1
jag2	1	0	0	0	0	0	0	0	0	0	0	0	0	0	1
ptbp3	1	0	0	0	0	0	0	0	0	0	0	0	0	0	1
si:dkey-8e10.2	1	0	0	0	0	0	0	0	0	0	0	0	0	0	1
ezrb	1	0	0	0	0	0	0	0	0	0	0	0	0	0	1
csrnp1b	1	0	0	0	0	0	0	0	0	0	0	0	0	0	1
tmem201	1	0	0	0	0	0	0	0	0	0	0	0	0	0	1
sp3a	1	0	0	0	0	0	0	0	0	0	0	0	0	0	1
gpr160	1	0	0	0	0	0	0	0	0	0	0	0	0	0	1
usp20	1	0	0	0	0	0	0	0	0	0	0	0	0	0	1
hectd1	1	0	0	0	0	0	0	0	0	0	0	0	0	0	1
dnajb12b	1	0	0	0	0	0	0	0	0	0	0	0	0	0	1
pgm2l1	1	0	0	0	0	0	0	0	0	0	0	0	0	0	1
rbfox2	1	0	0	0	0	0	0	0	0	0	0	0	0	0	1
cep104	1	0	0	0	0	0	0	0	0	0	0	0	0	0	1
cracr2a	1	0	0	0	0	0	0	0	0	0	0	0	0	0	1
traf3ip1	1	0	0	0	0	0	0	0	0	0	0	0	0	0	1
atp2b1a	1	0	0	0	0	0	0	0	0	0	0	0	0	0	1
zbtb10	1	0	0	0	0	0	0	0	0	0	0	0	0	0	1
lrig1	1	0	0	0	0	0	0	0	0	0	0	0	0	0	1
ncapg2	1	0	0	0	0	0	0	0	0	0	0	0	0	0	1
kcnc4	1	0	0	0	0	0	0	0	0	0	0	0	0	0	1
gpam	1	0	0	0	0	0	0	0	0	0	0	0	0	0	1
atic	1	0	0	0	0	0	0	0	0	0	0	0	0	0	1
cdc42bpa	1	0	0	0	0	0	0	0	0	0	0	0	0	0	1
znf576.2	1	0	0	0	0	0	0	0	0	0	0	0	0	0	1
stk35l	1	0	0	0	0	0	0	0	0	0	0	0	0	0	1
tiam1a	1	0	0	0	0	0	0	0	0	0	0	0	0	0	1
lrrc61	1	0	0	0	0	0	0	0	0	0	0	0	0	0	1
haus8	1	0	0	0	0	0	0	0	0	0	0	0	0	0	1
sh2b1	1	0	0	0	0	0	0	0	0	0	0	0	0	0	1
slc25a51a	1	0	0	0	0	0	0	0	0	0	0	0	0	0	1
rxfp2a	1	0	0	0	0	0	0	0	0	0	0	0	0	0	1
znf296	1	0	0	0	0	0	0	0	0	0	0	0	0	0	1

bcl2l13	1	0	0	0	0	0	0	0	0	0	0	0	0	0	1
slco1c1	1	0	0	0	0	0	0	0	0	0	0	0	0	0	1
zgc:152951	1	0	0	0	0	0	0	0	0	0	0	0	0	0	1
prpf4ba	1	0	0	0	0	0	0	0	0	0	0	0	0	0	1
rhobtb2a	1	0	0	0	0	0	0	0	0	0	0	0	0	0	1
brf2	1	0	0	0	0	0	0	0	0	0	0	0	0	0	1
btbd16	1	0	0	0	0	0	0	0	0	0	0	0	0	0	1
setd4	1	0	0	0	0	0	0	0	0	0	0	0	0	0	1
myo5aa	1	0	0	0	0	0	0	0	0	0	0	0	0	0	1
fam160a1a	1	0	0	0	0	0	0	0	0	0	0	0	0	0	1
neurod6a	1	0	0	0	0	0	0	0	0	0	0	0	0	0	1
xab2	1	0	0	0	0	0	0	0	0	0	0	0	0	0	1
ttc37	1	0	0	0	0	0	0	0	0	0	0	0	0	0	1
ulk4	1	0	0	0	0	0	0	0	0	0	0	0	0	0	1
inppl1b	1	0	0	0	0	0	0	0	0	0	0	0	0	0	1
acta1b	1	0	0	0	0	0	0	0	0	0	0	0	0	0	1
fnbp1l	1	0	0	0	0	0	0	0	0	0	0	0	0	0	1
adgra3	1	0	0	0	0	0	0	0	0	0	0	0	0	0	1
ankrd22	1	0	0	0	0	0	0	0	0	0	0	0	0	0	1
map2k4a	1	0	0	0	0	0	0	0	0	0	0	0	0	0	1
zgc:110329	1	0	0	0	0	0	0	0	0	0	0	0	0	0	1
pak2a	1	0	0	0	0	0	0	0	0	0	0	0	0	0	1
tnrc18	1	0	0	0	0	0	0	0	0	0	0	0	0	0	1
nr2f1b	1	0	0	0	0	0	0	0	0	0	0	0	0	0	1
camk2g1	1	0	0	0	0	0	0	0	0	0	0	0	0	0	1
ptprk	1	0	0	0	0	0	0	0	0	0	0	0	0	0	1
bin2b	1	0	0	0	0	0	0	0	0	0	0	0	0	0	1
ube2d4	1	0	0	0	0	0	0	0	0	0	0	0	0	0	1
magi3b	1	0	0	0	0	0	0	0	0	0	0	0	0	0	1
znf865	1	0	0	0	0	0	0	0	0	0	0	0	0	0	1
col23a1	1	0	0	0	0	0	0	0	0	0	0	0	0	0	1
rgs9bp	1	0	0	0	0	0	0	0	0	0	0	0	0	0	1
nfatc3a	1	0	0	0	0	0	0	0	0	0	0	0	0	0	1
si:ch211-157c3.4	1	0	0	0	0	0	0	0	0	0	0	0	0	0	1
kcnj1b	1	0	0	0	0	0	0	0	0	0	0	0	0	0	1
hydin	1	0	0	0	0	0	0	0	0	0	0	0	0	0	1
birc5a	1	0	0	0	0	0	0	0	0	0	0	0	0	0	1
nnt	1	0	0	0	0	0	0	0	0	0	0	0	0	0	1
mstnb	1	0	0	0	0	0	0	0	0	0	0	0	0	0	1
zfpm2a	1	0	0	0	0	0	0	0	0	0	0	0	0	0	1
maml3	1	0	0	0	0	0	0	0	0	0	0	0	0	0	1
gtf3c4	1	0	0	0	0	0	0	0	0	0	0	0	0	0	1
ppm1g	1	0	0	0	0	0	0	0	0	0	0	0	0	0	1
mier3a	1	0	0	0	0	0	0	0	0	0	0	0	0	0	1
zfp64	1	0	0	0	0	0	0	0	0	0	0	0	0	0	1
smurf2	1	0	0	0	0	0	0	0	0	0	0	0	0	0	1
snx27a	1	0	0	0	0	0	0	0	0	0	0	0	0	0	1
rims1a	1	0	0	0	0	0	0	0	0	0	0	0	0	0	1
si:dkeyp-68b7.7	1	0	0	0	0	0	0	0	0	0	0	0	0	0	1
bcl11aa	1	0	0	0	0	0	0	0	0	0	0	0	0	0	1
vezf1b	1	0	0	0	0	0	0	0	0	0	0	0	0	0	1
vps11	1	0	0	0	0	0	0	0	0	0	0	0	0	0	1
htt	1	0	0	0	0	0	0	0	0	0	0	0	0	0	1
kcnt2	1	0	0	0	0	0	0	0	0	0	0	0	0	0	1
eng	1	0	0	0	0	0	0	0	0	0	0	0	0	0	1
alg1	1	0	0	0	0	0	0	0	0	0	0	0	0	0	1
akap10	1	0	0	0	0	0	0	0	0	0	0	0	0	0	1
mknk2a	1	0	0	0	0	0	0	0	0	0	0	0	0	0	1
ppfibp1a	1	0	0	0	0	0	0	0	0	0	0	0	0	0	1
cbll1	1	0	0	0	0	0	0	0	0	0	0	0	0	0	1
eya3	1	0	0	0	0	0	0	0	0	0	0	0	0	0	1
wdfy3	1	0	0	0	0	0	0	0	0	0	0	0	0	0	1
zmynd11	1	0	0	0	0	0	0	0	0	0	0	0	0	0	1
gopc	1	0	0	0	0	0	0	0	0	0	0	0	0	0	1
tsr1	1	0	0	0	0	0	0	0	0	0	0	0	0	0	1
epha4b	1	0	0	0	0	0	0	0	0	0	0	0	0	0	1
ptpn21	1	0	0	0	0	0	0	0	0	0	0	0	0	0	1
ctr9	1	0	0	0	0	0	0	0	0	0	0	0	0	0	1
hnrnpua	1	0	0	0	0	0	0	0	0	0	0	0	0	0	1
trim35	1	0	0	0	0	0	0	0	0	0	0	0	0	0	1
stard14	1	0	0	0	0	0	0	0	0	0	0	0	0	0	1
pak4	1	0	0	0	0	0	0	0	0	0	0	0	0	0	1
slc19a2	1	0	0	0	0	0	0	0	0	0	0	0	0	0	1
hpse2	1	0	0	0	0	0	0	0	0	0	0	0	0	0	1
si:ch73-138n13.1	1	0	0	0	0	0	0	0	0	0	0	0	0	0	1
tmem138	1	0	0	0	0	0	0	0	0	0	0	0	0	0	1
tmem57a	1	0	0	0	0	0	0	0	0	0	0	0	0	0	1
ash1l	1	0	0	0	0	0	0	0	0	0	0	0	0	0	1
mtmr10	1	0	0	0	0	0	0	0	0	0	0	0	0	0	1
lrpprc	1	0	0	0	0	0	0	0	0	0	0	0	0	0	1
atp9b	1	0	0	0	0	0	0	0	0	0	0	0	0	0	1
rfng	1	0	0	0	0	0	0	0	0	0	0	0	0	0	1
znf106a	1	0	0	0	0	0	0	0	0	0	0	0	0	0	1
4-Mar	1	0	0	0	0	0	0	0	0	0	0	0	0	0	1
srebf1	1	0	0	0	0	0	0	0	0	0	0	0	0	0	1
tmbim1	1	0	0	0	0	0	0	0	0	0	0	0	0	0	1
ell	1	0	0	0	0	0	0	0	0	0	0	0	0	0	1
alg3	1	0	0	0	0	0	0	0	0	0	0	0	0	0	1
dkc1	1	0	0	0	0	0	0	0	0	0	0	0	0	0	1

tmem63a	1	0	0	0	0	0	0	0	0	0	0	0	0	0	1
ntrk3a	1	0	0	0	0	0	0	0	0	0	0	0	0	0	1
ypel1	1	0	0	0	0	0	0	0	0	0	0	0	0	0	1
arhgef4	1	0	0	0	0	0	0	0	0	0	0	0	0	0	1
phlpp1	1	0	0	0	0	0	0	0	0	0	0	0	0	0	1
amot	1	0	0	0	0	0	0	0	0	0	0	0	0	0	1
scai	1	0	0	0	0	0	0	0	0	0	0	0	0	0	1
kansl1b	1	0	0	0	0	0	0	0	0	0	0	0	0	0	1
hic1	1	0	0	0	0	0	0	0	0	0	0	0	0	0	1
igf2bp2a	1	0	0	0	0	0	0	0	0	0	0	0	0	0	1
nrbp1	1	0	0	0	0	0	0	0	0	0	0	0	0	0	1
elmsan1a	1	0	0	0	0	0	0	0	0	0	0	0	0	0	1
insra	1	0	0	0	0	0	0	0	0	0	0	0	0	0	1
src	1	0	0	0	0	0	0	0	0	0	0	0	0	0	1
cacnb4b	1	0	0	0	0	0	0	0	0	0	0	0	0	0	1
pik3ca	1	0	0	0	0	0	0	0	0	0	0	0	0	0	1
six5	1	0	0	0	0	0	0	0	0	0	0	0	0	0	1
tab3	1	0	0	0	0	0	0	0	0	0	0	0	0	0	1
dusp4	1	0	0	0	0	0	0	0	0	0	0	0	0	0	1
rufy1	1	0	0	0	0	0	0	0	0	0	0	0	0	0	1
ankrd28	1	0	0	0	0	0	0	0	0	0	0	0	0	0	1
klhl11	1	0	0	0	0	0	0	0	0	0	0	0	0	0	1
ubp1	1	0	0	0	0	0	0	0	0	0	0	0	0	0	1
reps1	1	0	0	0	0	0	0	0	0	0	0	0	0	0	1
slco4a1	1	0	0	0	0	0	0	0	0	0	0	0	0	0	1
kansl1a	1	0	0	0	0	0	0	0	0	0	0	0	0	0	1
prune	1	0	0	0	0	0	0	0	0	0	0	0	0	0	1
rab33ba	1	0	0	0	0	0	0	0	0	0	0	0	0	0	1
nelfa	1	0	0	0	0	0	0	0	0	0	0	0	0	0	1
ubtd1b	1	0	0	0	0	0	0	0	0	0	0	0	0	0	1
c18h15orf26	1	0	0	0	0	0	0	0	0	0	0	0	0	0	1
slc25a25b	1	0	0	0	0	0	0	0	0	0	0	0	0	0	1
sipa1l3	1	0	0	0	0	0	0	0	0	0	0	0	0	0	1
btbd2a	1	0	0	0	0	0	0	0	0	0	0	0	0	0	1
rreb1b	1	0	0	0	0	0	0	0	0	0	0	0	0	0	1
ctnnd1	1	0	0	0	0	0	0	0	0	0	0	0	0	0	1
athl1	1	0	0	0	0	0	0	0	0	0	0	0	0	0	1
si:ch211-207d6.2	1	0	0	0	0	0	0	0	0	0	0	0	0	0	1
pard3bb	1	0	0	0	0	0	0	0	0	0	0	0	0	0	1
chd6	1	0	0	0	0	0	0	0	0	0	0	0	0	0	1
diexf	1	0	0	0	0	0	0	0	0	0	0	0	0	0	1
msi2b	1	0	0	0	0	0	0	0	0	0	0	0	0	0	1
vdrb	1	0	0	0	0	0	0	0	0	0	0	0	0	0	1
znf1028	1	0	0	0	0	0	0	0	0	0	0	0	0	0	1
triob	1	0	0	0	0	0	0	0	0	0	0	0	0	0	1
ahcyl2	1	0	0	0	0	0	0	0	0	0	0	0	0	0	1
tpp2	1	0	0	0	0	0	0	0	0	0	0	0	0	0	1
brsk2	1	0	0	0	0	0	0	0	0	0	0	0	0	0	1
ano8b	1	0	0	0	0	0	0	0	0	0	0	0	0	0	1
hecw1b	1	0	0	0	0	0	0	0	0	0	0	0	0	0	1
sap130a	1	0	0	0	0	0	0	0	0	0	0	0	0	0	1
rnf26	1	0	0	0	0	0	0	0	0	0	0	0	0	0	1
ascc2	1	0	0	0	0	0	0	0	0	0	0	0	0	0	1
nek4	1	0	0	0	0	0	0	0	0	0	0	0	0	0	1
gdf6a	1	0	0	0	0	0	0	0	0	0	0	0	0	0	1
slc30a1b	1	0	0	0	0	0	0	0	0	0	0	0	0	0	1
tnpo1	1	0	0	0	0	0	0	0	0	0	0	0	0	0	1
phactr3a	1	0	0	0	0	0	0	0	0	0	0	0	0	0	1
lmbr1l	1	0	0	0	0	0	0	0	0	0	0	0	0	0	1
pou2f1b	1	0	0	0	0	0	0	0	0	0	0	0	0	0	1
ubl7a	1	0	0	0	0	0	0	0	0	0	0	0	0	0	1
sp3b	1	0	0	0	0	0	0	0	0	0	0	0	0	0	1
tbc1d8b	1	0	0	0	0	0	0	0	0	0	0	0	0	0	1
otx2	1	0	0	0	0	0	0	0	0	0	0	0	0	0	1
ranbp10	1	0	0	0	0	0	0	0	0	0	0	0	0	0	1
rnf111	1	0	0	0	0	0	0	0	0	0	0	0	0	0	1
hexim1	1	0	0	0	0	0	0	0	0	0	0	0	0	0	1
c18h19orf47	1	0	0	0	0	0	0	0	0	0	0	0	0	0	1
wdr48b	1	0	0	0	0	0	0	0	0	0	0	0	0	0	1
dnah7	1	0	0	0	0	0	0	0	0	0	0	0	0	0	1
socs5b	1	0	0	0	0	0	0	0	0	0	0	0	0	0	1
slc22a23	1	0	0	0	0	0	0	0	0	0	0	0	0	0	1
cux1a	1	0	0	0	0	0	0	0	0	0	0	0	0	0	1
mlec	1	0	0	0	0	0	0	0	0	0	0	0	0	0	1
pard3	1	0	0	0	0	0	0	0	0	0	0	0	0	0	1
si:dkey-52l18.4	1	0	0	0	0	0	0	0	0	0	0	0	0	0	1
spna2	1	0	0	0	0	0	0	0	0	0	0	0	0	0	1
robo1	1	0	0	0	0	0	0	0	0	0	0	0	0	0	1
ltbp3	1	0	0	0	0	0	0	0	0	0	0	0	0	0	1
klf13	1	0	0	0	0	0	0	0	0	0	0	0	0	0	1
ssh1a	1	0	0	0	0	0	0	0	0	0	0	0	0	0	1
zcchc11	1	0	0	0	0	0	0	0	0	0	0	0	0	0	1
nbn	1	0	0	0	0	0	0	0	0	0	0	0	0	0	1
zdhhc17	1	0	0	0	0	0	0	0	0	0	0	0	0	0	1
kdm6al	1	0	0	0	0	0	0	0	0	0	0	0	0	0	1
arhgap35a	1	0	0	0	0	0	0	0	0	0	0	0	0	0	1
sema3e	1	0	0	0	0	0	0	0	0	0	0	0	0	0	1
si:ch211-266d19.4	1	0	0	0	0	0	0	0	0	0	0	0	0	0	1
usp19	1	0	0	0	0	0	0	0	0	0	0	0	0	0	1

zgc:158564	1	0	0	0	0	0	0	0	0	0	0	0	0	0	1
mylk4a	1	0	0	0	0	0	0	0	0	0	0	0	0	0	1
irf2bp1	1	0	0	0	0	0	0	0	0	0	0	0	0	0	1
dacha	1	0	0	0	0	0	0	0	0	0	0	0	0	0	1
mrgbp	1	0	0	0	0	0	0	0	0	0	0	0	0	0	1
kifc3	1	0	0	0	0	0	0	0	0	0	0	0	0	0	1
pcgf6	1	0	0	0	0	0	0	0	0	0	0	0	0	0	1
si:ch211-10d23.5	1	0	0	0	0	0	0	0	0	0	0	0	0	0	1
b4galt3	1	0	0	0	0	0	0	0	0	0	0	0	0	0	1
ccdc9	1	0	0	0	0	0	0	0	0	0	0	0	0	0	1
hdac5	1	0	0	0	0	0	0	0	0	0	0	0	0	0	1
csrnp2	1	0	0	0	0	0	0	0	0	0	0	0	0	0	1
mtpap	1	0	0	0	0	0	0	0	0	0	0	0	0	0	1
tead3b	1	0	0	0	0	0	0	0	0	0	0	0	0	0	1
chst14	1	0	0	0	0	0	0	0	0	0	0	0	0	0	1
sytl5	1	0	0	0	0	0	0	0	0	0	0	0	0	0	1
igsf9b	1	0	0	0	0	0	0	0	0	0	0	0	0	0	1
si:ch1073-357b18.3	1	0	0	0	0	0	0	0	0	0	0	0	0	0	1
pramef1	1	0	0	0	0	0	0	0	0	0	0	0	0	0	1
lin54	1	0	0	0	0	0	0	0	0	0	0	0	0	0	1
ppat	1	0	0	0	0	0	0	0	0	0	0	0	0	0	1
ubr3	1	0	0	0	0	0	0	0	0	0	0	0	0	0	1
tsc22d2	1	0	0	0	0	0	0	0	0	0	0	0	0	0	1
tnfaip8l1	1	0	0	0	0	0	0	0	0	0	0	0	0	0	1
crtc1b	1	0	0	0	0	0	0	0	0	0	0	0	0	0	1
si:ch211-105d11.1	1	0	0	0	0	0	0	0	0	0	0	0	0	0	1
shroom4	1	0	0	0	0	0	0	0	0	0	0	0	0	0	1
fam89a	1	0	0	0	0	0	0	0	0	0	0	0	0	0	1
atxn1a	1	0	0	0	0	0	0	0	0	0	0	0	0	0	1
spag9a	1	0	0	0	0	0	0	0	0	0	0	0	0	0	1
adamtsl4	1	0	0	0	0	0	0	0	0	0	0	0	0	0	1
stk38a	1	0	0	0	0	0	0	0	0	0	0	0	0	0	1
rgmd	1	0	0	0	0	0	0	0	0	0	0	0	0	0	1
git1	1	0	0	0	0	0	0	0	0	0	0	0	0	0	1
kcnma1a	1	0	0	0	0	0	0	0	0	0	0	0	0	0	1
gps2	1	0	0	0	0	0	0	0	0	0	0	0	0	0	1
dusp3a	1	0	0	0	0	0	0	0	0	0	0	0	0	0	1
fcho2	1	0	0	0	0	0	0	0	0	0	0	0	0	0	1
plxnb3	1	0	0	0	0	0	0	0	0	0	0	0	0	0	1
cabz01055347.1	1	0	0	0	0	0	0	0	0	0	0	0	0	0	1
fbxw11b	1	0	0	0	0	0	0	0	0	0	0	0	0	0	1
slmapa	1	0	0	0	0	0	0	0	0	0	0	0	0	0	1
ptenb	1	0	0	0	0	0	0	0	0	0	0	0	0	0	1
setd1ba	1	0	0	0	0	0	0	0	0	0	0	0	0	0	1
rpp38	1	0	0	0	0	0	0	0	0	0	0	0	0	0	1
srm	1	0	0	0	0	0	0	0	0	0	0	0	0	0	1
galnt12	1	0	0	0	0	0	0	0	0	0	0	0	0	0	1
cacnb2b	1	0	0	0	0	0	0	0	0	0	0	0	0	0	1
rictorb	1	0	0	0	0	0	0	0	0	0	0	0	0	0	1
irs2a	1	0	0	0	0	0	0	0	0	0	0	0	0	0	1
rnf41l	1	0	0	0	0	0	0	0	0	0	0	0	0	0	1
dis3l2	1	0	0	0	0	0	0	0	0	0	0	0	0	0	1
ptpra	1	0	0	0	0	0	0	0	0	0	0	0	0	0	1
rnf144ab	1	0	0	0	0	0	0	0	0	0	0	0	0	0	1
fzd5	1	0	0	0	0	0	0	0	0	0	0	0	0	0	1
tnpo3	1	0	0	0	0	0	0	0	0	0	0	0	0	0	1
ccdc30	1	0	0	0	0	0	0	0	0	0	0	0	0	0	1
itsn2a	1	0	0	0	0	0	0	0	0	0	0	0	0	0	1
trmt1	1	0	0	0	0	0	0	0	0	0	0	0	0	0	1
dennd4a	1	0	0	0	0	0	0	0	0	0	0	0	0	0	1
taok2a	1	0	0	0	0	0	0	0	0	0	0	0	0	0	1
mon1bb	1	0	0	0	0	0	0	0	0	0	0	0	0	0	1
scel	1	0	0	0	0	0	0	0	0	0	0	0	0	0	1
grb10a	1	0	0	0	0	0	0	0	0	0	0	0	0	0	1
mri1	1	0	0	0	0	0	0	0	0	0	0	0	0	0	1
arfip1	1	0	0	0	0	0	0	0	0	0	0	0	0	0	1
mkl2a	1	0	0	0	0	0	0	0	0	0	0	0	0	0	1
zdhhc9	1	0	0	0	0	0	0	0	0	0	0	0	0	0	1
gatad2b	1	0	0	0	0	0	0	0	0	0	0	0	0	0	1
si:ch1073-219n12.1	1	0	0	0	0	0	0	0	0	0	0	0	0	0	1
si:ch211-51e12.7	1	0	0	0	0	0	0	0	0	0	0	0	0	0	1
foxk1	1	0	0	0	0	0	0	0	0	0	0	0	0	0	1
srfa	1	0	0	0	0	0	0	0	0	0	0	0	0	0	1
pxn	1	0	0	0	0	0	0	0	0	0	0	0	0	0	1
smu1a	1	0	0	0	0	0	0	0	0	0	0	0	0	0	1
shda	1	0	0	0	0	0	0	0	0	0	0	0	0	0	1
plcl1	1	0	0	0	0	0	0	0	0	0	0	0	0	0	1
memo1	1	0	0	0	0	0	0	0	0	0	0	0	0	0	1
mapk3	1	0	0	0	0	0	0	0	0	0	0	0	0	0	1
rps6ka3a	1	0	0	0	0	0	0	0	0	0	0	0	0	0	1
chst5	1	0	0	0	0	0	0	0	0	0	0	0	0	0	1
slc18a2	1	0	0	0	0	0	0	0	0	0	0	0	0	0	1
taf6l	1	0	0	0	0	0	0	0	0	0	0	0	0	0	1
ppfia4	1	0	0	0	0	0	0	0	0	0	0	0	0	0	1
rapgef1a	1	0	0	0	0	0	0	0	0	0	0	0	0	0	1
ppp2r5ea	1	0	0	0	0	0	0	0	0	0	0	0	0	0	1
asic2	1	0	0	0	0	0	0	0	0	0	0	0	0	0	1
pdpk1b	1	0	0	0	0	0	0	0	0	0	0	0	0	0	1
ppapdc2	1	0	0	0	0	0	0	0	0	0	0	0	0	0	1

tbc1d16	1	0	0	0	0	0	0	0	0	0	0	0	0	0	1
ern1	1	0	0	0	0	0	0	0	0	0	0	0	0	0	1
si:ch211-253p2.2	1	0	0	0	0	0	0	0	0	0	0	0	0	0	1
b4galnt1b	1	0	0	0	0	0	0	0	0	0	0	0	0	0	1
lin28a	1	0	0	0	0	0	0	0	0	0	0	0	0	0	1
ppp6r2b	1	0	0	0	0	0	0	0	0	0	0	0	0	0	1
dxo	1	0	0	0	0	0	0	0	0	0	0	0	0	0	1
zyg11	1	0	0	0	0	0	0	0	0	0	0	0	0	0	1
arid4b	1	0	0	0	0	0	0	0	0	0	0	0	0	0	1
tbcc	1	0	0	0	0	0	0	0	0	0	0	0	0	0	1
aatf	1	0	0	0	0	0	0	0	0	0	0	0	0	0	1
cacna1eb	1	0	0	0	0	0	0	0	0	0	0	0	0	0	1
ifngr1	1	0	0	0	0	0	0	0	0	0	0	0	0	0	1
c25h7orf60	1	0	0	0	0	0	0	0	0	0	0	0	0	0	1
ppm1j	1	0	0	0	0	0	0	0	0	0	0	0	0	0	1
pcyt1ba	1	0	0	0	0	0	0	0	0	0	0	0	0	0	1
cabz01038499.1	1	0	0	0	0	0	0	0	0	0	0	0	0	0	1
mbd3b	1	0	0	0	0	0	0	0	0	0	0	0	0	0	1
trpm4b.2	1	0	0	0	0	0	0	0	0	0	0	0	0	0	1
c5h9orf85	1	0	0	0	0	0	0	0	0	0	0	0	0	0	1
fbxl20	1	0	0	0	0	0	0	0	0	0	0	0	0	0	1
si:dkey-16j16.4	1	0	0	0	0	0	0	0	0	0	0	0	0	0	1
zc4h2	1	0	0	0	0	0	0	0	0	0	0	0	0	0	1
zdhhc7	1	0	0	0	0	0	0	0	0	0	0	0	0	0	1
zgc:158803	1	0	0	0	0	0	0	0	0	0	0	0	0	0	1
pak2b	1	0	0	0	0	0	0	0	0	0	0	0	0	0	1
esrp2	1	0	0	0	0	0	0	0	0	0	0	0	0	0	1
si:ch211-1e14.1	1	0	0	0	0	0	0	0	0	0	0	0	0	0	1
hip1	1	0	0	0	0	0	0	0	0	0	0	0	0	0	1
cbx1a	1	0	0	0	0	0	0	0	0	0	0	0	0	0	1
si:ch211-67e16.4	1	0	0	0	0	0	0	0	0	0	0	0	0	0	1
hemk1	1	0	0	0	0	0	0	0	0	0	0	0	0	0	1
pim3	1	0	0	0	0	0	0	0	0	0	0	0	0	0	1
tram2	1	0	0	0	0	0	0	0	0	0	0	0	0	0	1
rc3h1	1	0	0	0	0	0	0	0	0	0	0	0	0	0	1
saa	0	1	0	0	0	0	0	0	0	0	0	0	0	0	1
ppdpfb	0	1	0	0	0	0	0	0	0	0	0	0	0	0	1
si:dkey-14d8.7	0	1	0	0	0	0	0	0	0	0	0	0	0	0	1
si:ch211-194i10.5	0	1	0	0	0	0	0	0	0	0	0	0	0	0	1
myeov2	0	1	0	0	0	0	0	0	0	0	0	0	0	0	1
lgals2a	0	1	0	0	0	0	0	0	0	0	0	0	0	0	1
hsd17b7	0	1	0	0	0	0	0	0	0	0	0	0	0	0	1
tspo	0	1	0	0	0	0	0	0	0	0	0	0	0	0	1
si:ch1073-406l10.2	0	1	0	0	0	0	0	0	0	0	0	0	0	0	1
tagln2	0	1	0	0	0	0	0	0	0	0	0	0	0	0	1
lyrm5b	0	1	0	0	0	0	0	0	0	0	0	0	0	0	1
pvalb4	0	1	0	0	0	0	0	0	0	0	0	0	0	0	1
hspe1	0	1	0	0	0	0	0	0	0	0	0	0	0	0	1
tnni2a.1	0	1	0	0	0	0	0	0	0	0	0	0	0	0	1
rpl36a	0	1	0	0	0	0	0	0	0	0	0	0	0	0	1
ebpl	0	1	0	0	0	0	0	0	0	0	0	0	0	0	1
f11r.2	0	1	0	0	0	0	0	0	0	0	0	0	0	0	1
cu984579.1	0	1	0	0	0	0	0	0	0	0	0	0	0	0	1
gpd1b	0	1	0	0	0	0	0	0	0	0	0	0	0	0	1
cyba	0	1	0	0	0	0	0	0	0	0	0	0	0	0	1
rps8a	0	1	0	0	0	0	0	0	0	0	0	0	0	0	1
cr352328.3	0	1	0	0	0	0	0	0	0	0	0	0	0	0	1
s100v2	0	1	0	0	0	0	0	0	0	0	0	0	0	0	1
si:dkeyp-75b4.10	0	1	0	0	0	0	0	0	0	0	0	0	0	0	1
hmox2a	0	1	0	0	0	0	0	0	0	0	0	0	0	0	1
capns1b	0	1	0	0	0	0	0	0	0	0	0	0	0	0	1
fkbp2	0	1	0	0	0	0	0	0	0	0	0	0	0	0	1
tmed3	0	1	0	0	0	0	0	0	0	0	0	0	0	0	1
hsbp1b	0	1	0	0	0	0	0	0	0	0	0	0	0	0	1
sh3bgrl	0	1	0	0	0	0	0	0	0	0	0	0	0	0	1
si:ch211-244b2.4	0	1	0	0	0	0	0	0	0	0	0	0	0	0	1
ftr56	0	1	0	0	0	0	0	0	0	0	0	0	0	0	1
cnn2	0	1	0	0	0	0	0	0	0	0	0	0	0	0	1
sult1st4	0	1	0	0	0	0	0	0	0	0	0	0	0	0	1
cishb	0	1	0	0	0	0	0	0	0	0	0	0	0	0	1
hagh	0	1	0	0	0	0	0	0	0	0	0	0	0	0	1
lpar6a	0	1	0	0	0	0	0	0	0	0	0	0	0	0	1
rnaset2	0	1	0	0	0	0	0	0	0	0	0	0	0	0	1
traf3ip3	0	1	0	0	0	0	0	0	0	0	0	0	0	0	1
alpi.2	0	1	0	0	0	0	0	0	0	0	0	0	0	0	1
med11	0	1	0	0	0	0	0	0	0	0	0	0	0	0	1
si:ch1073-126c3.2	0	1	0	0	0	0	0	0	0	0	0	0	0	0	1
vps29	0	1	0	0	0	0	0	0	0	0	0	0	0	0	1
bdh2	0	1	0	0	0	0	0	0	0	0	0	0	0	0	1
si:ch211-284e13.9	0	1	0	0	0	0	0	0	0	0	0	0	0	0	1
dcun1d2b	0	1	0	0	0	0	0	0	0	0	0	0	0	0	1
pgrmc1	0	1	0	0	0	0	0	0	0	0	0	0	0	0	1
sdhdb	0	1	0	0	0	0	0	0	0	0	0	0	0	0	1
si:dkey-188i13.6	0	1	0	0	0	0	0	0	0	0	0	0	0	0	1
fp236810.1	0	1	0	0	0	0	0	0	0	0	0	0	0	0	1
gcnt4	0	1	0	0	0	0	0	0	0	0	0	0	0	0	1
glo1	0	1	0	0	0	0	0	0	0	0	0	0	0	0	1
pdcd6	0	1	0	0	0	0	0	0	0	0	0	0	0	0	1
lipca	0	1	0	0	0	0	0	0	0	0	0	0	0	0	1

cutc	0	1	0	0	0	0	0	0	0	0	0	0	0	0	1
zgc:158846	0	0	0	1	0	0	0	0	0	0	0	0	0	0	1
tmem97	0	0	0	1	0	0	0	0	0	0	0	0	0	0	1
hp	0	0	0	1	0	0	0	0	0	0	0	0	0	0	1
si:ch211-284o19.8	0	0	0	1	0	0	0	0	0	0	0	0	0	0	1
pdzk1	0	0	0	1	0	0	0	0	0	0	0	0	0	0	1
krtcap2	0	0	0	1	0	0	0	0	0	0	0	0	0	0	1
gstz1	0	0	0	1	0	0	0	0	0	0	0	0	0	0	1
rgs18	0	0	0	1	0	0	0	0	0	0	0	0	0	0	1
park7	0	0	0	1	0	0	0	0	0	0	0	0	0	0	1
gadd45aa	0	0	0	1	0	0	0	0	0	0	0	0	0	0	1
agt	0	0	0	1	0	0	0	0	0	0	0	0	0	0	1
tspan33a	0	0	0	1	0	0	0	0	0	0	0	0	0	0	1
spg21	0	0	0	1	0	0	0	0	0	0	0	0	0	0	1
capns1a	0	0	0	1	0	0	0	0	0	0	0	0	0	0	1
psma6l	0	0	0	1	0	0	0	0	0	0	0	0	0	0	1
si:dkey-180p18.9	0	0	0	1	0	0	0	0	0	0	0	0	0	0	1
dkk1b	0	0	0	1	0	0	0	0	0	0	0	0	0	0	1
si:ch73-308m11.1	0	0	0	1	0	0	0	0	0	0	0	0	0	0	1
agmo	0	0	0	1	0	0	0	0	0	0	0	0	0	0	1
ucmaa	0	0	0	1	0	0	0	0	0	0	0	0	0	0	1
si:ch73-111e15.1	0	0	0	1	0	0	0	0	0	0	0	0	0	0	1
scinlb	0	0	0	1	0	0	0	0	0	0	0	0	0	0	1
atp5g3a	0	0	0	1	0	0	0	0	0	0	0	0	0	0	1
ctns	0	0	0	1	0	0	0	0	0	0	0	0	0	0	1
cpb2	0	0	0	1	0	0	0	0	0	0	0	0	0	0	1
eef1b2	0	0	0	1	0	0	0	0	0	0	0	0	0	0	1
sec61g	0	0	0	0	1	0	0	0	0	0	0	0	0	0	1
zgc:92880	0	0	0	0	1	0	0	0	0	0	0	0	0	0	1
cox7a2a	0	0	0	0	1	0	0	0	0	0	0	0	0	0	1
endou	0	0	0	0	1	0	0	0	0	0	0	0	0	0	1
rhag	0	0	0	0	1	0	0	0	0	0	0	0	0	0	1
cox5aa	0	0	0	0	1	0	0	0	0	0	0	0	0	0	1
zgc:158446	0	0	0	0	1	0	0	0	0	0	0	0	0	0	1
wnt11r	0	0	0	0	1	0	0	0	0	0	0	0	0	0	1
tmem14a	0	0	0	0	1	0	0	0	0	0	0	0	0	0	1
ndufa8	0	0	0	0	1	0	0	0	0	0	0	0	0	0	1
rgs5b	0	0	0	0	1	0	0	0	0	0	0	0	0	0	1
tnni2a.3	0	0	0	0	1	0	0	0	0	0	0	0	0	0	1
ebp	0	0	0	0	1	0	0	0	0	0	0	0	0	0	1
cct7	0	0	0	0	1	0	0	0	0	0	0	0	0	0	1
hsd17b14	0	0	0	0	1	0	0	0	0	0	0	0	0	0	1
zgc:103601	0	0	0	0	1	0	0	0	0	0	0	0	0	0	1
magt1	0	0	0	0	1	0	0	0	0	0	0	0	0	0	1
aldh6a1	0	0	0	0	1	0	0	0	0	0	0	0	0	0	1
myl9b	0	0	0	0	1	0	0	0	0	0	0	0	0	0	1
nt5c3a	0	0	0	0	1	0	0	0	0	0	0	0	0	0	1
hpgd	0	0	0	0	1	0	0	0	0	0	0	0	0	0	1
si:dkey-58f10.7	0	0	0	0	1	0	0	0	0	0	0	0	0	0	1
spink4	0	0	0	0	0	0	1	0	0	0	0	0	0	0	1
gpx4a	0	0	0	0	0	0	1	0	0	0	0	0	0	0	1
g0s2	0	0	0	0	0	0	1	0	0	0	0	0	0	0	1
plac8.1	0	0	0	0	0	0	1	0	0	0	0	0	0	0	1
si:dkey-5n18.1	0	0	0	0	0	0	1	0	0	0	0	0	0	0	1
cox6b2	0	0	0	0	0	0	1	0	0	0	0	0	0	0	1
zgc:64106	0	0	0	0	0	0	1	0	0	0	0	0	0	0	1
ccl34b.4	0	0	0	0	0	0	1	0	0	0	0	0	0	0	1
apom	0	0	0	0	0	0	1	0	0	0	0	0	0	0	1
rbp2a	0	0	0	0	0	0	1	0	0	0	0	0	0	0	1
si:ch211-156j16.1	0	0	0	0	0	0	1	0	0	0	0	0	0	0	1
tldc1	0	0	0	0	0	0	1	0	0	0	0	0	0	0	1
ckap4	0	0	0	0	0	0	1	0	0	0	0	0	0	0	1
rpl38	0	0	0	0	0	0	1	0	0	0	0	0	0	0	1
c25h12orf73	0	0	0	0	0	0	1	0	0	0	0	0	0	0	1
rps21	0	0	0	0	0	0	1	0	0	0	0	0	0	0	1
scpp8	0	0	0	0	0	0	1	0	0	0	0	0	0	0	1
ctsh	0	0	0	0	0	0	1	0	0	0	0	0	0	0	1
atp5l	0	0	0	0	0	0	1	0	0	0	0	0	0	0	1
samsn1a	0	0	0	0	0	0	1	0	0	0	0	0	0	0	1
selt1b	0	0	0	0	0	0	1	0	0	0	0	0	0	0	1
uqcrc2a	0	0	0	0	0	0	1	0	0	0	0	0	0	0	1
gfpt2	0	0	0	0	0	0	1	0	0	0	0	0	0	0	1
cox4i2	0	0	0	0	0	0	1	0	0	0	0	0	0	0	1
psma2	0	0	0	0	0	0	1	0	0	0	0	0	0	0	1
emp2	0	0	0	0	0	0	1	0	0	0	0	0	0	0	1
apip	0	0	0	0	0	0	1	0	0	0	0	0	0	0	1
ms4a17a.17	0	0	0	0	0	0	1	0	0	0	0	0	0	0	1
tnfsf13b	0	0	0	0	0	0	1	0	0	0	0	0	0	0	1
bada	0	0	0	0	0	0	1	0	0	0	0	0	0	0	1
cecr1a	0	0	0	0	0	0	1	0	0	0	0	0	0	0	1
h1fx	0	0	0	0	0	0	1	0	0	0	0	0	0	0	1
gda	0	0	0	0	0	0	1	0	0	0	0	0	0	0	1
bre	0	0	0	0	0	0	1	0	0	0	0	0	0	0	1
bbip1	0	0	0	0	0	0	1	0	0	0	0	0	0	0	1
zgc:194392	0	0	0	0	0	0	1	0	0	0	0	0	0	0	1
entpd1	0	0	0	0	0	0	1	0	0	0	0	0	0	0	1
cd151	0	0	0	0	0	0	1	0	0	0	0	0	0	0	1
tprg1	0	0	0	0	0	0	1	0	0	0	0	0	0	0	1
med31	0	0	0	0	0	0	1	0	0	0	0	0	0	0	1

smim15	0	0	0	0	0	0	1	0	0	0	0	0	0	0	1
si:dkey-276j7.1	0	0	0	0	0	0	1	0	0	0	0	0	0	0	1
atp6v1f	0	0	0	0	0	0	1	0	0	0	0	0	0	0	1
aldh1a2	0	0	0	0	0	0	1	0	0	0	0	0	0	0	1
bida	0	0	0	0	0	0	1	0	0	0	0	0	0	0	1
si:dkey-172o19.2	0	0	0	0	0	0	1	0	0	0	0	0	0	0	1
mydgf	0	0	0	0	0	0	1	0	0	0	0	0	0	0	1
desmb	0	0	0	0	0	0	1	0	0	0	0	0	0	0	1
s100a10b	0	0	0	0	0	0	0	1	0	0	0	0	0	0	1
cela1	0	0	0	0	0	0	0	1	0	0	0	0	0	0	1
grn1	0	0	0	0	0	0	0	1	0	0	0	0	0	0	1
ppdpfa	0	0	0	0	0	0	0	1	0	0	0	0	0	0	1
b2m	0	0	0	0	0	0	0	1	0	0	0	0	0	0	1
si:ch211-222l21.1	0	0	0	0	0	0	0	1	0	0	0	0	0	0	1
sdhc	0	0	0	0	0	0	0	1	0	0	0	0	0	0	1
gabarapa	0	0	0	0	0	0	0	1	0	0	0	0	0	0	1
tmem123	0	0	0	0	0	0	0	1	0	0	0	0	0	0	1
rpl22	0	0	0	0	0	0	0	1	0	0	0	0	0	0	1
cox7a2	0	0	0	0	0	0	0	1	0	0	0	0	0	0	1
ifit15	0	0	0	0	0	0	0	1	0	0	0	0	0	0	1
nfkbiaa	0	0	0	0	0	0	0	1	0	0	0	0	0	0	1
sec61b	0	0	0	0	0	0	0	1	0	0	0	0	0	0	1
usp41	0	0	0	0	0	0	0	1	0	0	0	0	0	0	1
c5h9orf16	0	0	0	0	0	0	0	1	0	0	0	0	0	0	1
si:ch1073-291c23.2	0	0	0	0	0	0	0	1	0	0	0	0	0	0	1
ufd1l	0	0	0	0	0	0	0	1	0	0	0	0	0	0	1
rassf6	0	0	0	0	0	0	0	1	0	0	0	0	0	0	1
uqcrc1	0	0	0	0	0	0	0	1	0	0	0	0	0	0	1
si:ch211-170d8.8	0	0	0	0	0	0	0	1	0	0	0	0	0	0	1
rpl12	0	0	0	0	0	0	0	1	0	0	0	0	0	0	1
acadm	0	0	0	0	0	0	0	1	0	0	0	0	0	0	1
bri3	0	0	0	0	0	0	0	1	0	0	0	0	0	0	1
rpl21	0	0	0	0	0	0	0	1	0	0	0	0	0	0	1
polr2l	0	0	0	0	0	0	0	1	0	0	0	0	0	0	1
pfkfb3	0	0	0	0	0	0	0	1	0	0	0	0	0	0	1
zfyve21	0	0	0	0	0	0	0	1	0	0	0	0	0	0	1
dram2b	0	0	0	0	0	0	0	1	0	0	0	0	0	0	1
mtx2	0	0	0	0	0	0	0	1	0	0	0	0	0	0	1
desi1b	0	0	0	0	0	0	0	1	0	0	0	0	0	0	1
lect2	0	0	0	0	0	0	0	0	1	0	0	0	0	0	1
prdx2	0	0	0	0	0	0	0	0	1	0	0	0	0	0	1
gstm.2	0	0	0	0	0	0	0	0	1	0	0	0	0	0	1
acat2	0	0	0	0	0	0	0	0	1	0	0	0	0	0	1
si:ch211-195o20.7	0	0	0	0	0	0	0	0	1	0	0	0	0	0	1
dram1	0	0	0	0	0	0	0	0	1	0	0	0	0	0	1
si:ch211-217k17.12	0	0	0	0	0	0	0	0	1	0	0	0	0	0	1
zgc:110540	0	0	0	0	0	0	0	0	1	0	0	0	0	0	1
allc	0	0	0	0	0	0	0	0	1	0	0	0	0	0	1
cfb	0	0	0	0	0	0	0	0	1	0	0	0	0	0	1
nfil3	0	0	0	0	0	0	0	0	1	0	0	0	0	0	1
luzp2	0	0	0	0	0	0	0	0	1	0	0	0	0	0	1
atp5f1	0	0	0	0	0	0	0	0	1	0	0	0	0	0	1
c13h10orf54	0	0	0	0	0	0	0	0	1	0	0	0	0	0	1
glipr1a	0	0	0	0	0	0	0	0	1	0	0	0	0	0	1
coq9	0	0	0	0	0	0	0	0	1	0	0	0	0	0	1
lsm6	0	0	0	0	0	0	0	0	1	0	0	0	0	0	1
si:ch211-117c9.5	0	0	0	0	0	0	0	0	1	0	0	0	0	0	1
zgc:100906	0	0	0	0	0	0	0	0	1	0	0	0	0	0	1
ftr13	0	0	0	0	0	0	0	0	1	0	0	0	0	0	1
prkab2	0	0	0	0	0	0	0	0	1	0	0	0	0	0	1
hmgn3	0	0	0	0	0	0	0	0	0	1	0	0	0	0	1
tmod4	0	0	0	0	0	0	0	0	0	1	0	0	0	0	1
bhmt	0	0	0	0	0	0	0	0	0	1	0	0	0	0	1
dnajc19	0	0	0	0	0	0	0	0	0	1	0	0	0	0	1
sult2st1	0	0	0	0	0	0	0	0	0	1	0	0	0	0	1
zgc:153968	0	0	0	0	0	0	0	0	0	1	0	0	0	0	1
dnajc12	0	0	0	0	0	0	0	0	0	1	0	0	0	0	1
isca1	0	0	0	0	0	0	0	0	0	1	0	0	0	0	1
psmb10	0	0	0	0	0	0	0	0	0	1	0	0	0	0	1
c1h19orf53	0	0	0	0	0	0	0	0	0	1	0	0	0	0	1
cd36	0	0	0	0	0	0	0	0	0	1	0	0	0	0	1
si:ch211-202h22.9	0	0	0	0	0	0	0	0	0	1	0	0	0	0	1
rps27.2	0	0	0	0	0	0	0	0	0	1	0	0	0	0	1
sh3glb1b	0	0	0	0	0	0	0	0	0	1	0	0	0	0	1
crb3b	0	0	0	0	0	0	0	0	0	1	0	0	0	0	1
uqcrc2b	0	0	0	0	0	0	0	0	0	1	0	0	0	0	1
tifa	0	0	0	0	0	0	0	0	0	1	0	0	0	0	1
sap30l	0	0	0	0	0	0	0	0	0	1	0	0	0	0	1
tnfb	0	0	0	0	0	0	0	0	0	1	0	0	0	0	1
copz1	0	0	0	0	0	0	0	0	0	1	0	0	0	0	1
txnipa	0	0	0	0	0	0	0	0	0	1	0	0	0	0	1
gapdhs	0	0	0	0	0	0	0	0	0	1	0	0	0	0	1
tmem150b	0	0	0	0	0	0	0	0	0	1	0	0	0	0	1
rsad2	0	0	0	0	0	0	0	0	0	0	1	0	0	0	1
tlx1	0	0	0	0	0	0	0	0	0	0	1	0	0	0	1
suclg1	0	0	0	0	0	0	0	0	0	0	1	0	0	0	1
c7h15orf43	0	0	0	0	0	0	0	0	0	0	1	0	0	0	1
yipf6	0	0	0	0	0	0	0	0	0	0	1	0	0	0	1
clca1	0	0	0	0	0	0	0	0	0	0	1	0	0	0	1

bx571811.1	0	0	0	0	0	0	0	0	0	0	1	0	0	0	1
ppcdc	0	0	0	0	0	0	0	0	0	0	1	0	0	0	1
ppp1r18	0	0	0	0	0	0	0	0	0	0	1	0	0	0	1
slc43a2a	0	0	0	0	0	0	0	0	0	0	1	0	0	0	1
cyp2p9	0	0	0	0	0	0	0	0	0	0	1	0	0	0	1
bag3	0	0	0	0	0	0	0	0	0	0	1	0	0	0	1
zgc:174895	0	0	0	0	0	0	0	0	0	0	1	0	0	0	1
amn1	0	0	0	0	0	0	0	0	0	0	1	0	0	0	1
atp6v1e1a	0	0	0	0	0	0	0	0	0	0	1	0	0	0	1
vwa7	0	0	0	0	0	0	0	0	0	0	1	0	0	0	1
acat1	0	0	0	0	0	0	0	0	0	0	1	0	0	0	1
cyp2j20	0	0	0	0	0	0	0	0	0	0	1	0	0	0	1
prdx5	0	0	0	0	0	0	0	0	0	0	0	1	0	0	1
rnasel3	0	0	0	0	0	0	0	0	0	0	0	1	0	0	1
cmbl	0	0	0	0	0	0	0	0	0	0	0	1	0	0	1
fabp7a	0	0	0	0	0	0	0	0	0	0	0	1	0	0	1
sf3b6	0	0	0	0	0	0	0	0	0	0	0	1	0	0	1
esd	0	0	0	0	0	0	0	0	0	0	0	1	0	0	1
pphln1	0	0	0	0	0	0	0	0	0	0	0	1	0	0	1
soul5	0	0	0	0	0	0	0	0	0	0	0	1	0	0	1
mmp9	0	0	0	0	0	0	0	0	0	0	0	1	0	0	1
fabp6	0	0	0	0	0	0	0	0	0	0	0	1	0	0	1
cytip	0	0	0	0	0	0	0	0	0	0	0	1	0	0	1
rcl1	0	0	0	0	0	0	0	0	0	0	0	1	0	0	1
zgc:153911	0	0	0	0	0	0	0	0	0	0	0	1	0	0	1
mob3c	0	0	0	0	0	0	0	0	0	0	0	1	0	0	1
tbca	0	0	0	0	0	0	0	0	0	0	0	1	0	0	1
fam213aa	0	0	0	0	0	0	0	0	0	0	0	1	0	0	1
pon1	0	0	0	0	0	0	0	0	0	0	0	1	0	0	1
sftpba	0	0	0	0	0	0	0	0	0	0	0	1	0	0	1
neu3.3	0	0	0	0	0	0	0	0	0	0	0	1	0	0	1
pfkfb1	0	0	0	0	0	0	0	0	0	0	0	1	0	0	1
ppa1b	0	0	0	0	0	0	0	0	0	0	0	1	0	0	1
si:dkey-21o19.2	0	0	0	0	0	0	0	0	0	0	0	1	0	0	1
atp6ap2	0	0	0	0	0	0	0	0	0	0	0	1	0	0	1
zgc:123068	0	0	0	0	0	0	0	0	0	0	0	0	1	0	1
etfb	0	0	0	0	0	0	0	0	0	0	0	0	1	0	1
serpina7	0	0	0	0	0	0	0	0	0	0	0	0	1	0	1
cu019646.2	0	0	0	0	0	0	0	0	0	0	0	0	1	0	1
si:ch211-186e20.6	0	0	0	0	0	0	0	0	0	0	0	0	1	0	1
slc2a12	0	0	0	0	0	0	0	0	0	0	0	0	1	0	1
arpc2	0	0	0	0	0	0	0	0	0	0	0	0	1	0	1
si:ch211-218c6.8	0	0	0	0	0	0	0	0	0	0	0	0	1	0	1
krt18	0	0	0	0	0	0	0	0	0	0	0	0	1	0	1
si:ch211-173n18.3	0	0	0	0	0	0	0	0	0	0	0	0	1	0	1
slco2b1	0	0	0	0	0	0	0	0	0	0	0	0	1	0	1
si:dkey-261l7.2	0	0	0	0	0	0	0	0	0	0	0	0	1	0	1
zgc:171686	0	0	0	0	0	0	0	0	0	0	0	0	1	0	1
c9h21orf33	0	0	0	0	0	0	0	0	0	0	0	0	1	0	1
hmgcra	0	0	0	0	0	0	0	0	0	0	0	0	1	0	1
si:ch211-186e20.7	0	0	0	0	0	0	0	0	0	0	0	0	1	0	1
shfm1	0	0	0	0	0	0	0	0	0	0	0	0	1	0	1
lrg1	0	0	0	0	0	0	0	0	0	0	0	0	1	0	1
bccip	0	0	0	0	0	0	0	0	0	0	0	0	1	0	1
rrm2b	0	0	0	0	0	0	0	0	0	0	0	0	1	0	1
aimp2	0	0	0	0	0	0	0	0	0	0	0	0	1	0	1
grap2a	0	0	0	0	0	0	0	0	0	0	0	0	1	0	1
gpx4b	0	0	0	0	0	0	0	0	0	0	0	0	0	1	1
apoa2	0	0	0	0	0	0	0	0	0	0	0	0	0	1	1
atp5g3b	0	0	0	0	0	0	0	0	0	0	0	0	0	1	1
fabp3	0	0	0	0	0	0	0	0	0	0	0	0	0	1	1
fth1a	0	0	0	0	0	0	0	0	0	0	0	0	0	1	1
rnf7	0	0	0	0	0	0	0	0	0	0	0	0	0	1	1
gpx1a	0	0	0	0	0	0	0	0	0	0	0	0	0	1	1
pvalb3	0	0	0	0	0	0	0	0	0	0	0	0	0	1	1
emd	0	0	0	0	0	0	0	0	0	0	0	0	0	1	1
azin1b	0	0	0	0	0	0	0	0	0	0	0	0	0	1	1
zgc:158409	0	0	0	0	0	0	0	0	0	0	0	0	0	1	1
akap12b	0	0	0	0	0	0	0	0	0	0	0	0	0	1	1
