# Supplementary material for: The Plasticizer Bisphenol A Perturbs the Hepatic Epigenome: A Systems Level Analysis of the miRNome
Source: Genes (Basel). 2017 Oct 13;8(10):269. doi: 10.3390/genes8100269 (PMC5664119; doi:10.3390/genes8100269)
Supplement: Supplementary file 1 [file genes-08-00269-s001.zip › zip_GENES_supplemental materials/_Supplemental Tables.pdf]

Supplemental Table S1 (Matrix) is a separate pdf file that can be found in supplemental materials section.

Supplemental Table S2: Merge of the miRNA targets with the genes selected for each modules. When a gene present in a module is not a predicted target of miRNA, non-applicable (NA) was entered in "miRNA" column. This table also contains log2 fold change ("FoldChange") and adjusted p-value ("P-value FDR") columns. Certain genes belong to two modules and in that case a sub-module was created, i.e. Epigenetics/Apop-Autophag for DNMT1. Abbreviations: Apop-Autophag = Apoptosis & Autophagy, Oxi-phospho = Oxidative Phosphorylation.

Gene	Module	miRNA	FoldChange	q-value FDR
map1lc3c	Apoptosis & Autophagy	NA	4.42	1.45E-06
map1sa	Apoptosis & Autophagy	NA	3.82	2.51E-05
bcl9	Apoptosis & Autophagy	NA	4.04	4.92E-05
itpr2	Apoptosis & Autophagy	dre-miR-2189	3.50	5.38E-05
casp8ap2	Apoptosis & Autophagy	dre-miR-2189	3.25	3.07E-04
parp1	Apoptosis & Autophagy	NA	3.09	4.36E-04
prf1.7	Apoptosis & Autophagy	NA	-3.38	6.79E-04
map3k4	Apoptosis & Autophagy	NA	3.15	1.01E-03
gabarapb	Apoptosis & Autophagy	dre-miR-122	-2.43	1.42E-03
gabarapb	Apoptosis & Autophagy	dre-miR-724	-2.43	1.42E-03
gabarapl1	Apoptosis & Autophagy	NA	-2.46	1.56E-03
hspa8	Apoptosis & Autophagy	NA	3.08	2.05E-03
hspe1	Apoptosis & Autophagy	dre-miR-499	-2.28	2.06E-03
gabarapl2	Apoptosis & Autophagy	NA	-2.28	2.22E-03
hspa4a	Apoptosis & Autophagy	dre-miR-2189	2.72	2.31E-03
caspa	Apoptosis & Autophagy	NA	-2.75	2.39E-03
gabarapa	Apoptosis & Autophagy	dre-miR-133a	-2.18	3.28E-03
lmnb1	Apoptosis & Autophagy	NA	2.64	3.45E-03
tp53bp2b	Apoptosis & Autophagy	NA	3.17	3.63E-03
mapk1	Apoptosis & Autophagy	dre-miR-2189	2.55	3.92E-03
grid1b	Apoptosis & Autophagy	NA	4.36	5.91E-03
tnfaip2b	Apoptosis & Autophagy	NA	-2.56	6.08E-03
ctsh	Apoptosis & Autophagy	dre-miR-205	-2.17	7.04E-03
map7d2b	Apoptosis & Autophagy	NA	3.89	7.32E-03
map2k6	Apoptosis & Autophagy	NA	2.30	8.22E-03
ambra1b	Apoptosis & Autophagy	NA	2.30	9.01E-03
dram1	Apoptosis & Autophagy	dre-miR-430b	-2.27	9.27E-03
epg5	Apoptosis & Autophagy	dre-miR-2189	2.22	9.79E-03
ambra1a	Apoptosis & Autophagy	dre-miR-2189	2.25	1.05E-02
map4k4	Apoptosis & Autophagy	NA	2.19	1.09E-02
daxx	Apoptosis & Autophagy	NA	2.33	1.17E-02
rtn1a	Apoptosis & Autophagy	NA	2.55	1.21E-02

Gene	Module	miRNA	FoldChange	q-value FDR
map7d3	Apoptosis & Autophagy	dre-miR-2189	2.15	1.30E-02
ctsbb	Apoptosis & Autophagy	NA	-2.55	1.47E-02
gadd45ga	Apoptosis & Autophagy	NA	-2.00	1.50E-02
mapk9	Apoptosis & Autophagy	dre-miR-2189	2.40	1.62E-02
map10	Apoptosis & Autophagy	NA	3.45	1.68E-02
uvrag	Apoptosis & Autophagy	NA	2.23	1.71E-02
itpr3	Apoptosis & Autophagy	NA	2.37	1.82E-02
nfkbiaa	Apoptosis & Autophagy	dre-miR-133a	-1.91	1.86E-02
ctsz	Apoptosis & Autophagy	dre-miR-430a	-1.73	2.20E-02
ctsz	Apoptosis & Autophagy	dre-miR-499	-1.73	2.20E-02
ctsz	Apoptosis & Autophagy	dre-miR-724	-1.73	2.20E-02
bcl2l13	Apoptosis & Autophagy	dre-miR-2189	2.23	2.25E-02
bada	Apoptosis & Autophagy	dre-miR-205	-1.85	2.33E-02
tnfaip6	Apoptosis & Autophagy	NA	-2.36	2.40E-02
map2k4a	Apoptosis & Autophagy	dre-miR-2189	2.08	2.57E-02
birc5a	Apoptosis & Autophagy	dre-miR-2189	2.17	2.81E-02
itpr1b	Apoptosis & Autophagy	NA	2.01	2.87E-02
bcl11aa	Apoptosis & Autophagy	dre-miR-2189	3.01	2.99E-02
htt	Apoptosis & Autophagy	dre-miR-2189	1.81	3.01E-02
gopc	Apoptosis & Autophagy	dre-miR-2189	1.90	3.25E-02
tnfaip8l3	Apoptosis & Autophagy	dre-miR-205	-2.29	3.46E-02
tnfaip8l3	Apoptosis & Autophagy	dre-miR-724	-2.29	3.46E-02
atg101	Apoptosis & Autophagy	NA	-1.66	3.51E-02
atg2a	Apoptosis & Autophagy	NA	1.69	3.64E-02
pik3ca	Apoptosis & Autophagy	dre-miR-2189	1.90	4.03E-02
aifm1	Apoptosis & Autophagy	NA	1.65	4.47E-02
rtn4ip1	Apoptosis & Autophagy	dre-miR-724	-1.68	5.06E-02
rtn4ip1	Apoptosis & Autophagy	dre-miR-725	-1.68	5.06E-02
bcl2a	Apoptosis & Autophagy	NA	2.46	5.09E-02
tuba8l2	Apoptosis & Autophagy	NA	-1.58	5.30E-02
casp6l2	Apoptosis & Autophagy	NA	-2.49	5.33E-02
atg4c	Apoptosis & Autophagy	NA	-1.65	5.43E-02
hspd1	Apoptosis & Autophagy	NA	1.57	5.50E-02
apaf1	Apoptosis & Autophagy	NA	1.64	5.61E-02
ctso	Apoptosis & Autophagy	dre-miR-193a	-1.66	5.63E-02
ctso	Apoptosis & Autophagy	dre-miR-205	-1.66	5.63E-02
map7	Apoptosis & Autophagy	NA	1.62	5.68E-02
atg2b	Apoptosis & Autophagy	NA	2.82	5.73E-02
grid2ipb	Apoptosis & Autophagy	NA	2.90	5.84E-02
atm	Apoptosis & Autophagy	NA	1.67	6.14E-02

Gene	Module	miRNA	FoldChange	q-value FDR
map1lc3a	Apoptosis & Autophagy	dre-miR-205	-1.58	6.65E-02
map1lc3a	Apoptosis & Autophagy	dre-miR-724	-1.58	6.65E-02
tnfaip8l1	Apoptosis & Autophagy	dre-miR-2189	1.64	6.79E-02
atg16l1	Apoptosis & Autophagy	NA	1.59	7.02E-02
diablob	Apoptosis & Autophagy	NA	-1.46	7.46E-02
ctsk	Apoptosis & Autophagy	NA	-1.49	7.59E-02
dram2b	Apoptosis & Autophagy	dre-miR-133a	-1.51	8.14E-02
tp53bp1	Apoptosis & Autophagy	NA	1.55	8.24E-02
baxa	Apoptosis & Autophagy	NA	-2.21	8.34E-02
mapk3	Apoptosis & Autophagy	dre-miR-2189	1.39	8.37E-02
map4	Apoptosis & Autophagy	NA	1.90	8.43E-02
pdpk1b	Apoptosis & Autophagy	dre-miR-2189	1.48	8.62E-02
map3k14	Apoptosis & Autophagy	NA	3.09	8.67E-02
ern1	Apoptosis & Autophagy	dre-miR-2189	1.57	8.81E-02
akt2	Apoptosis & Autophagy	NA	1.42	9.47E-02
atg12	Apoptosis & Autophagy	NA	-1.49	9.56E-02
map1lc3b	Apoptosis & Autophagy	NA	-1.32	9.74E-02
map1lc3b	Apoptosis & Autophagy	NA	-1.32	9.74E-02
gadd45ab	Apoptosis & Autophagy	NA	-1.45	1.10E-01
map4	Apoptosis & Autophagy	NA	-1.94	1.13E-01
hspa8	Apoptosis & Autophagy	NA	-0.89	2.75E-01
tuba8l4	Apoptosis & Autophagy	NA	0.90	3.50E-01
baxa	Apoptosis & Autophagy	NA	-0.73	4.30E-01
cflara	Apoptosis & Autophagy	NA	-0.70	4.52E-01
map3k14	Apoptosis & Autophagy	NA	-0.44	6.86E-01
ccnb1	Cell cycle	NA	5.01	4.04E-08
ccnb2	Cell cycle	NA	5.18	4.04E-08
orc1	Cell cycle	NA	4.82	1.18E-07
cdc20	Cell cycle	NA	4.58	1.99E-07
plk1	Cell cycle	NA	4.64	2.40E-07
espl1	Cell cycle	NA	4.76	2.45E-07
ccna1	Cell cycle	NA	5.11	2.66E-07
mcm2	Cell cycle	NA	4.52	4.58E-07
wee2	Cell cycle	NA	4.76	5.03E-07
bub1	Cell cycle	NA	4.60	1.29E-06
cdc6	Cell cycle	NA	4.33	1.87E-06
cdc7	Cell cycle	NA	4.45	3.06E-06
ccna2	Cell cycle	NA	4.07	4.92E-06
cdc45	Cell cycle	NA	4.06	6.84E-06
ccne1	Cell cycle	NA	4.18	1.04E-05

Gene	Module	miRNA	FoldChange	q-value FDR
anapc1	Cell cycle	NA	4.02	2.22E-05
mcm5	Cell cycle	NA	3.82	2.22E-05
mcm4	Cell cycle	NA	3.84	4.22E-05
skp2	Cell cycle	NA	3.70	5.56E-05
bub1bb	Cell cycle	NA	4.12	7.14E-05
bub1ba	Cell cycle	NA	3.68	1.17E-04
ttk	Cell cycle	NA	3.47	1.53E-04
chek2	Cell cycle	NA	3.44	2.59E-04
cdc25b	Cell cycle	NA	3.49	3.42E-04
chek1	Cell cycle	NA	3.46	3.58E-04
cdc16	Cell cycle	dre-miR-2189	3.02	1.05E-03
e2f5	Cell cycle	dre-miR-2189	3.20	1.22E-03
e2f3	Cell cycle	NA	2.95	1.53E-03
cdc27	Cell cycle	NA	2.80	1.72E-03
crebbpb	Cell cycle	NA	2.67	2.38E-03
crebbpa	Cell cycle	NA	2.64	2.57E-03
smad2	Cell cycle	dre-miR-2189	2.74	2.85E-03
gadd45bb	Cell cycle	dre-miR-122	-3.04	3.54E-03
gadd45bb	Cell cycle	dre-miR-133a	-3.04	3.54E-03
gadd45bb	Cell cycle	dre-miR-725	-3.04	3.54E-03
stag1b	Cell cycle	NA	2.55	3.82E-03
mad2l1	Cell cycle	NA	2.80	3.84E-03
orc6	Cell cycle	NA	2.71	4.48E-03
mad1l1	Cell cycle	dre-miR-2189	2.55	4.77E-03
ep300a	Cell cycle	NA	2.26	5.83E-03
anapc11	Cell cycle	NA	-2.15	6.41E-03
atr	Cell cycle	NA	2.54	7.88E-03
rbx1	Cell cycle	NA	-2.00	8.13E-03
dnmt1	Epigenetics/Apop-Autophag	NA	4.99	5.42E-08
dnmt3bb.2	Epigenetics/Apop-Autophag	NA	4.93	8.43E-07
dnmt3bb.3	Epigenetics/Apop-Autophag	NA	3.97	2.63E-05
dnmt3bb.1	Epigenetics/Apop-Autophag	NA	2.68	3.63E-03
ezh2	Epigenetics/Apop-Autophag	dre-miR-2189	2.51	4.55E-03
dnmt3ba	Epigenetics/Apop-Autophag	NA	2.25	3.46E-02
sirt7	Epigenetics/Apop-Autophag	NA	2.17	3.69E-02
hdac4	Epigenetics/Apop-Autophag	NA	2.35	5.54E-02
hdac11	Epigenetics/Apop-Autophag	NA	2.39	5.81E-02
hdac5	Epigenetics/Apop-Autophag	dre-miR-2189	1.81	6.34E-02
sirt2	Epigenetics/Apop-Autophag	NA	-1.55	6.95E-02
sirt5	Epigenetics/Apop-Autophag	dre-miR-122	-1.46	8.41E-02

Gene	Module	miRNA	FoldChange	q-value FDR
sirt5	Epigenetics/Apop-Autophag	dre-miR-193a	-1.46	8.41E-02
sirt5	Epigenetics/Apop-Autophag	dre-miR-430i	-1.46	8.41E-02
sirt5	Epigenetics/Apop-Autophag	dre-miR-499	-1.46	8.41E-02
sirt5	Epigenetics/Apop-Autophag	dre-miR-724	-1.46	8.41E-02
ndufb1	NAFLD	NA	-3.09	1.87E-05
socs3b	NAFLD	dre-miR-202	-2.33	8.73E-03
socs3b	NAFLD	dre-miR-205	-2.33	8.73E-03
socs3b	NAFLD	dre-miR-724	-2.33	8.73E-03
insrb	NAFLD	dre-miR-2189	2.16	8.81E-03
ins	NAFLD/ Apop-Autophag	NA	-4.65	7.88E-08
traf2a	NAFLD/ Apop-Autophag	NA	3.03	1.19E-03
eif2ak3	NAFLD/ Apop-Autophag	NA	2.33	7.29E-03
atp5e	Oxidative Phosphorylation	NA	-2.75	1.38E-04
atp5ia	Oxidative Phosphorylation	NA	-2.80	2.11E-04
atp5j	Oxidative Phosphorylation	NA	-2.71	4.16E-04
atp5g3b	Oxidative Phosphorylation	dre-miR-184	-2.57	4.74E-04
atp5o	Oxidative Phosphorylation	NA	-2.57	5.14E-04
atp5ib	Oxidative Phosphorylation	NA	-2.34	2.37E-03
atp5h	Oxidative Phosphorylation	NA	-2.26	3.11E-03
atp5c1	Oxidative Phosphorylation	NA	-2.08	4.74E-03
atp5l	Oxidative Phosphorylation	dre-miR-205	-2.01	8.01E-03
atp5j	Oxidative Phosphorylation	NA	-1.41	3.04E-01
atp5j	Oxidative Phosphorylation	NA	0.30	8.02E-01
cox7c	Oxi-phospho/NAFLD	NA	-3.37	1.52E-06
cox7a2a	Oxi-phospho/NAFLD	dre-miR-202	-3.22	4.43E-06
ndufa5	Oxi-phospho/NAFLD	NA	-3.19	7.58E-06
cox7b	Oxi-phospho/NAFLD	NA	-3.07	2.39E-05
ndufa11	Oxi-phospho/NAFLD	NA	-3.00	2.45E-05
uqcrq	Oxi-phospho/NAFLD	NA	-2.97	5.03E-05
ndufa3	Oxi-phospho/NAFLD	dre-miR-724	-2.92	8.03E-05
ndufa3	Oxi-phospho/NAFLD	dre-miR-725	-2.92	8.03E-05
ndufs4	Oxi-phospho/NAFLD	dre-miR-133a	-2.72	1.68E-04
ndufs4	Oxi-phospho/NAFLD	dre-miR-205	-2.72	1.68E-04
ndufs4	Oxi-phospho/NAFLD	dre-miR-724	-2.72	1.68E-04
ndufs4	Oxi-phospho/NAFLD	dre-miR-725	-2.72	1.68E-04
ndufa1	Oxi-phospho/NAFLD	NA	-2.76	1.90E-04
ndufs7	Oxi-phospho/NAFLD	NA	-2.61	3.73E-04
ndufb7	Oxi-phospho/NAFLD	NA	-2.67	3.76E-04
cox5ab	Oxi-phospho/NAFLD	NA	-2.56	4.86E-04
ndufb11	Oxi-phospho/NAFLD	NA	-2.56	5.30E-04

Gene	Module	miRNA	FoldChange	q-value FDR
cox6b1	Oxi-phospho/NAFLD	NA	-2.85	6.68E-04
ndufb6	Oxi-phospho/NAFLD	NA	-2.50	7.87E-04
ndufb9	Oxi-phospho/NAFLD	dre-miR-122	-2.46	8.21E-04
ndufb9	Oxi-phospho/NAFLD	dre-miR-202	-2.46	8.21E-04
ndufb9	Oxi-phospho/NAFLD	dre-miR-499	-2.46	8.21E-04
ndufb9	Oxi-phospho/NAFLD	dre-miR-724	-2.46	8.21E-04
ndufb9	Oxi-phospho/NAFLD	dre-miR-725	-2.46	8.21E-04
ndufc1	Oxi-phospho/NAFLD	NA	-2.47	1.02E-03
ndufc2	Oxi-phospho/NAFLD	NA	-2.51	1.42E-03
sdhc	Oxi-phospho/NAFLD	dre-miR-133a	-2.34	1.48E-03
cox6c	Oxi-phospho/NAFLD	NA	-2.36	1.56E-03
ndufb8	Oxi-phospho/NAFLD	NA	-2.47	1.62E-03
ndufab1a	Oxi-phospho/NAFLD	NA	-2.29	1.80E-03
uqcrh	Oxi-phospho/NAFLD	dre-miR-193a	-2.31	1.91E-03
uqcrh	Oxi-phospho/NAFLD	dre-miR-724	-2.31	1.91E-03
uqcrh	Oxi-phospho/NAFLD	dre-miR-725	-2.31	1.91E-03
ndufa7	Oxi-phospho/NAFLD	NA	-2.36	1.92E-03
ndufa7	Oxi-phospho/NAFLD	NA	-2.36	1.92E-03
ndufa6	Oxi-phospho/NAFLD	NA	-2.36	2.71E-03
ndufs3	Oxi-phospho/NAFLD	NA	-2.21	2.78E-03
ndufb10	Oxi-phospho/NAFLD	dre-miR-193a	-2.25	3.13E-03
ndufb10	Oxi-phospho/NAFLD	dre-miR-205	-2.25	3.13E-03
ndufa2	Oxi-phospho/NAFLD	NA	-2.23	3.20E-03
ndufs6	Oxi-phospho/NAFLD	NA	-2.17	3.63E-03
ndufb2	Oxi-phospho/NAFLD	NA	-2.08	6.20E-03
cox5aa	Oxi-phospho/NAFLD	dre-miR-202	-2.11	6.27E-03
gpr63	Receptors	dre-miR-2189	3.83	1.61E-04
gpr161	Receptors	NA	3.54	3.41E-04
esr1	Receptors	NA	3.50	6.18E-04
trip10	Receptors	NA	4.49	1.68E-03
pth1a	Receptors	NA	4.73	1.69E-03
pth2r	Receptors	NA	5.23	2.93E-03
gpr179	Receptors	NA	4.60	3.32E-03
gpr183a	Receptors	NA	-2.61	5.88E-03
trip6	Receptors	dre-miR-2189	2.67	6.12E-03
trip11	Receptors	NA	2.03	9.05E-03
gpr61	Receptors	NA	4.73	9.95E-03
trip13	Receptors	NA	2.61	1.00E-02
gpr160	Receptors	dre-miR-2189	2.70	1.88E-02
gpr141	Receptors	NA	-2.77	1.88E-02

Gene	Module	miRNA	FoldChange	q-value FDR
rgra	Receptors	NA	-3.99	2.34E-02
gpr182	Receptors	dre-miR-205	-1.78	4.21E-02
gpr182	Receptors	dre-miR-499	-1.78	4.21E-02
gpr155a	Receptors	NA	1.72	5.18E-02
trip12	Receptors	NA	1.59	5.84E-02
gpr137	Receptors	NA	-1.66	5.91E-02
esrrga	Receptors	NA	3.10	6.32E-02
gprc5ba	Receptors	NA	3.23	6.59E-02
esrp2	Receptors	dre-miR-2189	1.33	9.66E-02
gpr161	Receptors	NA	-0.31	8.43E-01
pth2r	Receptors	NA	2.26	NA

Supplemental Table S3: Expression levels of vitellogenin (VTG) and zona pellucida (ZP) genes in the liver of exposed zebrafish.

Symbol	Description	Log-2 fold change	q-value
VTG1	Vitellogenin-1	5.107946	7.16E-07
VTG2	Vitellogenin-2	4.895476	3.59E-06
VTG3	Vitellogenin-3	4.555119	1.28E-05
VTG4	Vitellogenin-4	5.001526	3.39E-06
VTG5	Vitellogenin-5	4.890923	5.49E-06
VTG6	Vitellogenin-6	4.803922	5.85E-06
VTG7	Vitellogenin-7	5.008955	1.46E-06
ZP2.1	zona pellucida glycoprotein 2, tandem duplicate 1	5.050344	3.27E-06
ZP2.3	zona pellucida glycoprotein 2, tandem duplicate 3	5.631561	1.31E-08
ZP2.5	zona pellucida glycoprotein 2, tandem duplicate 5	5.384728	4.42E-07
ZP2.6	zona pellucida glycoprotein 2, tandem duplicate 6	5.126355	4.21E-07
ZP2L1	zona pellucida glycoprotein 2, like 1	5.301779	8.62E-08
ZP2L2	zona pellucida glycoprotein 2, like 2	5.719074	5.03E-07
ZP3.2	zona pellucida glycoprotein 3, tandem duplicate 2	5.360202	7.07E-08
ZP3A.1	zona pellucida glycoprotein 3a, tandem duplicate 1	5.435937	7.88E-08
ZP3A.2	zona pellucida glycoprotein 3a, tandem duplicate 2	5.51911	4.13E-08
ZP3B	zona pellucida glycoprotein 3b	5.496	4.04E-08
ZP3C	zona pellucida glycoprotein 3c	5.240709	2.33E-07
ZPCX	zona pellucida protein C	5.13068	2.07E-07

Supplemental Table S4: Advaita-iPathwayGuide analysis – Pathways. Top 40 pathways.

PATHWAYS

Name	q-value FDR
Oxidative phosphorylation	2.66E-08
Ribosome	2.66E-08
Alzheimer's disease	3.97E-05
Non-alcoholic fatty liver disease (NAFLD)	1.32E-04
Parkinson's disease	3.39E-04
Huntington's disease	5.14E-04
Cell cycle	1.54E-03
Oocyte meiosis	1.83E-03
Fanconi anemia pathway	1.83E-03
Pancreatic secretion	2.52E-03
Homologous recombination	3.06E-03
Cardiac muscle contraction	3.91E-03
Adherens junction	2.64E-02
Salivary secretion	1.28E-01
Progesterone-mediated oocyte maturation	1.33E-01
Linoleic acid metabolism	1.57E-01
p53 signaling pathway	4.00E-01
Glutathione metabolism	4.00E-01
Salmonella infection	4.00E-01
MicroRNAs in cancer	4.89E-01
Dorso-ventral axis formation	6.62E-01
Lysine degradation	6.62E-01
Metabolism of xenobiotics by cytochrome P450	6.62E-01
African trypanosomiasis	6.62E-01
Gastric acid secretion	6.65E-01
Inflammatory mediator regulation of TRP channels	6.82E-01
Drug metabolism - cytochrome P450	6.82E-01
Chemical carcinogenesis	8.58E-01
Retinol metabolism	9.66E-01
Renin-angiotensin system	9.66E-01
Systemic lupus erythematosus	9.66E-01
Non-small cell lung cancer	9.66E-01
Proteasome	9.66E-01
Vibrio cholerae infection	9.66E-01
Retrograde endocannabinoid signaling	9.66E-01
Pathogenic Escherichia coli infection	9.66E-01
Synaptic vesicle cycle	9.66E-01
Autophagy	9.66E-01
Synthesis and degradation of ketone bodies	9.66E-01
Signaling pathways regulating pluripotency of stem cells	9.66E-01

Supplemental Table S5: Advaita-iPathwayGuide analysis – Biological Process. Top 40 terms.

BIOLOGICAL PROCESS

Name	q-value FDR
cell cycle	5.33E-20
cell cycle process	1.28E-19
mitotic cell cycle process	2.31E-14
nuclear division	3.46E-14
mitotic cell cycle	4.26E-14
chromosome organization	1.03E-11
organelle fission	1.05E-11
protein targeting to ER	5.33E-11
SRP-dependent cotranslational protein targeting to membrane	6.15E-11
chromosome segregation	1.38E-10
viral transcription	2.23E-10
nucleobase-containing compound metabolic process	3.64E-10
establishment of protein localization to endoplasmic reticulum	5.00E-10
mitotic nuclear division	6.92E-10
meiotic cell cycle	9.23E-10
nuclear-transcribed mRNA catabolic process, nonsense-mediated decay	9.99E-10
cotranslational protein targeting to membrane	1.48E-09
heterocycle metabolic process	1.48E-09
cellular aromatic compound metabolic process	1.85E-09
organic cyclic compound metabolic process	2.88E-09
nuclear chromosome segregation	4.26E-09
multi-organism metabolic process	5.33E-09
ATP synthesis coupled electron transport	5.56E-09
meiotic cell cycle process	8.88E-09
mitochondrial ATP synthesis coupled electron transport	1.07E-08
protein localization to endoplasmic reticulum	1.11E-08
viral gene expression	1.42E-08
nucleic acid metabolic process	2.05E-08
cell division	3.67E-08
sister chromatid segregation	6.75E-08
chromatin organization	8.59E-08
meiotic nuclear division	1.23E-07
oxidative phosphorylation	1.74E-07
DNA metabolic process	2.76E-07
respiratory electron transport chain	2.89E-07
mitotic cell cycle phase transition	3.25E-07
double-strand break repair	5.46E-07
sister chromatid cohesion	5.46E-07
electron transport chain	5.46E-07
cell cycle phase transition	8.25E-07

Supplemental Table S6: Advaita-iPathwayGuide analysis – Molecular Function. Top 40 terms.

MOLECULAR FUNCTION

Name	q-value FDR
nucleic acid binding	4.59E-07
poly(A) RNA binding	2.45E-05
NADH dehydrogenase activity	2.45E-05
NADH dehydrogenase (ubiquinone) activity	2.45E-05
NADH dehydrogenase (quinone) activity	2.45E-05
oxidoreductase activity, acting on NAD(P)H, quinone or similar compound as acceptor	3.10E-04
helicase activity	3.20E-04
DNA binding	4.46E-04
RNA binding	1.91E-03
DNA helicase activity	1.91E-03
cytoskeletal protein binding	6.49E-03
microtubule binding	7.64E-03
hydrogen ion transmembrane transporter activity	8.82E-03
histone binding	1.49E-02
DNA-dependent ATPase activity	1.70E-02
ATPase activity	1.75E-02
tubulin binding	1.80E-02
mRNA 5'-UTR binding	1.84E-02
organic cyclic compound binding	2.41E-02
oxidoreductase activity, acting on NAD(P)H	3.19E-02
heterocyclic compound binding	3.21E-02
chromatin binding	3.21E-02
nuclear localization sequence binding	3.21E-02
flap endonuclease activity	3.72E-02
cadherin binding	3.82E-02
Ran GTPase binding	3.82E-02
protein binding involved in cell-cell adhesion	6.04E-02
cadherin binding involved in cell-cell adhesion	6.10E-02
structural constituent of ribosome	7.07E-02
endodeoxyribonuclease activity, producing 5'-phosphomonoesters	7.07E-02
microtubule plus-end binding	7.07E-02
protein binding involved in cell adhesion	8.84E-02
cytochrome-c oxidase activity	9.10E-02
heme-copper terminal oxidase activity	9.10E-02
oxidoreductase activity, acting on a heme group of donors, oxygen as acceptor	9.10E-02
histone methyltransferase activity	9.41E-02
ATP-dependent helicase activity	9.92E-02
purine NTP-dependent helicase activity	9.92E-02
glutathione peroxidase activity	1.18E-01
ubiquitin-like protein-specific protease activity	1.18E-01

Supplemental Table S7: Advaita-iPathwayGuide analysis – Cellular Component. Top 40 terms.

CELLULAR COMPONENT

Name	q-value FDR
nuclear part	8.72E-19
nuclear lumen	3.60E-18
non-membrane-bounded organelle	3.46E-16
intracellular non-membrane-bounded organelle	3.46E-16
nucleus	6.23E-16
nucleoplasm	6.23E-16
chromosome	4.35E-14
cytosolic ribosome	2.42E-13
chromosomal part	6.46E-13
cytosolic large ribosomal subunit	4.43E-09
respiratory chain complex	5.65E-09
mitochondrial respiratory chain	5.65E-09
respiratory chain	8.30E-09
membrane-enclosed lumen	1.30E-08
organelle lumen	1.30E-08
intracellular organelle lumen	1.30E-08
chromosome, centromeric region	2.93E-07
intracellular organelle part	4.08E-07
chromosomal region	4.08E-07
mitochondrial respiratory chain complex I	5.79E-07
NADH dehydrogenase complex	5.79E-07
respiratory chain complex I	5.79E-07
nuclear chromosome	1.20E-06
organelle part	1.67E-06
cytoskeletal part	2.21E-06
inner mitochondrial membrane protein complex	2.87E-06
cytoskeleton	2.92E-06
spindle	3.95E-06
chromatin	4.61E-06
macromolecular complex	4.61E-06
ribosomal subunit	8.48E-06
cytosolic part	9.08E-06
microtubule cytoskeleton	1.01E-05
nuclear chromosome part	1.42E-05
cytosolic small ribosomal subunit	5.54E-05
centrosome	7.69E-05
oxidoreductase complex	9.73E-05
nucleoplasm part	1.20E-04
condensed chromosome	1.56E-04
mitochondrial membrane part	2.01E-04

Supplemental Table S8: ToppFun functional enrichment analysis of the mRNAs that are predicted targets of miRNAs of interest – Pathways. A total of 15 pathways were enriched.

PATHWAYS

Name	q-value FDR
Non-alcoholic fatty liver disease (NAFLD)	1.17E-02
Oxidative phosphorylation	1.21E-02
Metabolic pathways	1.21E-02
Neutrophil degranulation	1.21E-02
oxidative phosphorylation	1.21E-02
Steroid Biosynthesis	1.28E-02
Glutathione metabolism	1.28E-02
AMPK signaling pathway	1.28E-02
The citric acid (TCA) cycle and respiratory electron transport	1.58E-02
superpathway of cholesterol biosynthesis	3.48E-02
Parkinson's disease	3.56E-02
Respiratory electron transport, ATP synthesis by chemiosmotic coupling, and heat production by uncoupling proteins.	4.24E-02
Adherens junction	4.24E-02
Signal attenuation	4.24E-02
Insulin signaling pathway	4.70E-02

Supplemental Table S9: ToppFun functional enrichment analysis of the mRNAs that are predicted targets of miRNAs of interest – Biological Process. Top 20 terms.

BIOLOGICAL PROCESSES

Name	q-value FDR
nucleoside metabolic process	2.58E-06
glycosyl compound metabolic process	2.58E-06
purine ribonucleoside metabolic process	3.37E-06
purine nucleoside metabolic process	3.43E-06
ribonucleoside metabolic process	5.34E-06
purine ribonucleoside monophosphate metabolic process	7.57E-06
purine nucleoside monophosphate metabolic process	7.57E-06
ribonucleoside monophosphate metabolic process	7.57E-06
nucleobase-containing small molecule metabolic process	7.57E-06
nucleoside monophosphate metabolic process	2.55E-05
purine-containing compound metabolic process	2.62E-05
ATP metabolic process	4.24E-05
oxidation-reduction process	9.88E-05
cellular respiration	1.56E-04
energy derivation by oxidation of organic compounds	1.69E-04
nucleoside phosphate metabolic process	2.25E-04
purine nucleoside triphosphate metabolic process	2.25E-04
nucleotide metabolic process	2.33E-04
purine ribonucleoside triphosphate metabolic process	2.47E-04
nucleoside triphosphate metabolic process	2.69E-04

Supplemental Table S10: ToppFun functional enrichment analysis of the mRNAs that are predicted targets of miRNAs of interest – Molecular Function. A total of 2 molecular functions were enriched.

MOLECULAR FUNCTIONS

Name	q-value FDR
glutathione peroxidase activity	1.95E-02
oxidoreductase activity	1.95E-02

Supplemental Table S11: ToppFun functional enrichment analysis of the mRNAs that are predicted targets of miRNAs of interest – Cellular Component. Top 20 terms.

CELLULAR COMPONENTS

Name	q-value FDR
adherens junction	2.82E-04
mitochondrial respiratory chain	2.82E-04
anchoring junction	4.06E-04
respiratory chain	5.61E-04
focal adhesion	6.53E-04
cell-substrate adherens junction	7.74E-04
cell-substrate junction	9.36E-04
oxidoreductase complex	1.05E-03
respiratory chain complex	1.58E-03
inner mitochondrial membrane protein complex	1.85E-03
catalytic complex	1.85E-03
mitochondrion	3.65E-03
myelin sheath	5.54E-03
mitochondrial membrane part	8.73E-03
cell junction	9.31E-03
mitochondrial protein complex	9.56E-03
organelle envelope	1.07E-02
cytosolic large ribosomal subunit	1.12E-02
envelope	1.12E-02
cell-cell junction	2.77E-02

Supplemental Table S12: Advaita-iPathwayGuide analysis of all DE genes that are predicted targets of miRNAs of interest – Pathways. Top 10 pathways.

PATHWAYS

Name	q-value FDR
Adherens junction	0.070968
Oxidative phosphorylation	0.070968
Chemical carcinogenesis	0.377428
Parkinson's disease	0.377428
Drug metabolism - cytochrome P450	0.377428
Ribosome	0.435353
Alzheimer's disease	0.662809
Metabolism of xenobiotics by cytochrome P450	0.743272
Non-alcoholic fatty liver disease (NAFLD)	0.743272
Axon guidance	0.999317

Supplemental Table S13: Advaita-iPathwayGuide analysis of all DE genes that are predicted targets of miRNAs of interest – Cellular Component. Top 10 terms.

CELLULAR COMPONENT

Name	q-value FDR
respiratory chain	0.07361
mitochondrial respiratory chain	0.07361
cytosolic large ribosomal subunit	0.145488
inner mitochondrial membrane protein complex	0.145488
nuclear lumen	0.145488
mitochondrial membrane part	0.14722
cytosolic ribosome	0.14722
respiratory chain complex	0.14722
cytosol	0.187922
cell-cell junction	0.187922
